# Unveiling interactions between intervertebral disc morphologies and mechanical behavior through personalized finite element modeling

**DOI:** 10.3389/fbioe.2024.1384599

**Published:** 2024-06-10

**Authors:** Estefano Muñoz-Moya, Morteza Rasouligandomani, Carlos Ruiz Wills, Francis Kiptengwer Chemorion, Gemma Piella, Jérôme Noailly

**Affiliations:** ^1^ BCN MedTech, Department of Engineering, Universitat Pompeu Fabra, Barcelona, Spain; ^2^ Department of Information Technology, InSilicoTrials Technologies, Trieste, Italy

**Keywords:** patient-specific, intervertebral disc, morphing algorithm, finite element method, machine learning, model repository, patient-personalized, morphological analysis

## Abstract

**Introduction:** Intervertebral Disc (IVD) Degeneration (IDD) is a significant health concern, potentially influenced by mechanotransduction. However, the relationship between the IVD phenotypes and mechanical behavior has not been thoroughly explored in local morphologies where IDD originates. This work unveils the interplays among morphological and mechanical features potentially relevant to IDD through Abaqus UMAT simulations.

**Methods:** A groundbreaking automated method is introduced to transform a calibrated, structured IVD finite element (FE) model into 169 patient-personalized (PP) models through a mesh morphing process. Our approach accurately replicates the real shapes of the patient's Annulus Fibrosus (AF) and Nucleus Pulposus (NP) while maintaining the same topology for all models. Using segmented magnetic resonance images from the former project *MySpine*, 169 models with structured hexahedral meshes were created employing the Bayesian Coherent Point Drift++ technique, generating a unique cohort of PP FE models under the *Disc4All* initiative. Machine learning methods, including Linear Regression, Support Vector Regression, and eXtreme Gradient Boosting Regression, were used to explore correlations between IVD morphology and mechanics.

**Results:** We achieved PP models with AF and NP similarity scores of 92.06\% and 92.10\% compared to the segmented images. The models maintained good quality and integrity of the mesh. The cartilage endplate (CEP) shape was represented at the IVD-vertebra interfaces, ensuring personalized meshes. Validation of the constitutive model against literature data showed a minor relative error of 5.20%.

**Discussion:** Analysis revealed the influential impact of local morphologies on indirect mechanotransduction responses, highlighting the roles of heights, sagittal areas, and volumes. While the maximum principal stress was influenced by morphologies such as heights, the disc's ellipticity influenced the minimum principal stress. Results suggest the CEPs are not influenced by their local morphologies but by those of the AF and NP. The generated free-access repository of individual disc characteristics is anticipated to be a valuable resource for the scientific community with a broad application spectrum.

## 1 Introduction

Lumbar degenerative spine diseases are the primary cause of low back pain (LBP), standing as a leading global health burden. They affect an estimated 266 million individuals worldwide annually and are a primary cause of work absenteeism, as highlighted by the meta-analysis of Ravindra et al. (2018). Intervertebral disc (IVD) degeneration (IDD) is identified as the primary cause of LBP. It results from multiple factors, including heredity, aging, inadequate metabolite transport, and mechanical loading. These factors can collectively compromise the disc’s integrity and increase the risk of physical disruption under physiological mechanical loads (Adams and Roughley, 2006). Despite the broad and profound impact of IDD, there is still a lack of effective early detection of risk factors and therapies.

The IVD, the largest avascular tissue in the human body, provides the spine with load support and flexibility while finely articulating the anterior vertebral column. This function results from fully functional interactions among the highly specialized disc tissues. The disc tissues define three main anatomic regions: (i) the center, with the gelatinous nucleus pulposus (NP), populated by chondrocyte-like cells with a density of around 5,000 cells/mm^3^ (Kodama et al., 2023). It predominantly contains proteoglycans reinforced with type-II collagen fibers for structural integrity that promote tissue hydration and hydrostatic pressurization through osmotic swelling (Urban et al., 2000); (ii) the circumference, with the annulus fibrosus (AF), a fiber-reinforced lamellar ring structure with concentric layers of type-I collagen aligned according to a criss-cross pattern that alternates in angles from 28° to 44° concerning the transverse plane of the disc (Natarajan et al., 2004; Nerurkar et al., 2007; Pezowicz, 2010; Raza and Michalek, 2021), laterally confines the NP and contributes to tensile strength when the periphery of the disc is directly stretched, undergoes shear deformations, or bulges under the effect of the intradiscal pressure (Kiani et al., 2002; Bhattacharjee and Ghosh, 2014); and (iii) the cranial and caudal ends, with the top and bottom cartilage endplates (CEP), a 500–1,000 *μ*m-thick layer of hyaline-like cartilage that consists mainly of type-II collagen, proteoglycans, and water (Roberts et al., 1989; Urban et al., 2004; Moon et al., 2013). It covers the NP’s cranial and caudal ends and the AF’s inner part adjacent to the vertebral subchondral bone, i.e., the bony endplate (BEP).

The risk factors for IDD encompass a range of physiological and genetic contributors. These include excessive movements and loads (Paul et al., 2013), genetic predispositions (Mayer et al., 2013), previous spinal surgeries (Hashimoto et al., 2018), anomalies in the vertebral endplates (Bonnheim et al., 2022), and a reduction in proteoglycans accompanied by decreased water content in the discs (Knudson and Knudson, 2001). Even recent research has underscored the significant role of disc morphology in IDD, mainly focusing on the disc’s height and associated stiffness (Tavana et al., 2024).

Significant advancements have been made in understanding the biological interactions relevant to IDD through *in silico* modeling techniques (Baumgartner et al., 2021). These advancements address challenges such as the complex nonlinear behavior of IVD components, the invasive nature of studying human internal structures, and the difficulties in replicating physiological conditions like pressure variations and nutrient supply in *in vivo* and *in vitro* environments (Sato et al., 1999).

Computational modeling of the IVD predominantly employs finite element (FE) analysis, agent-based models, and network models (Bermudez-Lekerika et al., 2022). Furthermore, experimental *in vivo* measurements, such as those by Wilke et al. (1999), have provided valuable data on disc pressures during varied activities, offering unique physiological IVD mechanical loads for FE analyses. These simulations have explored early IDD aspects, including cell nutritional stress (Ruiz et al., 2018), cell viability (Ruiz et al., 2016), the effects of sustained compression (Malandrino et al., 2011), and the impact of nutrient supply (Malandrino et al., 2014).

Despite these advances, integrating these insights into FE models to create patient-personalized (PP) IVD models to study IDD remains challenging. One major hurdle is the presence of oversimplified geometrical representations (Meijer et al., 2011). A recent study by Fleps et al. (2024) aimed to bridge this gap by investigating the influence of IVD morphology on its mechanical behaviors through FE analysis. However, the study primarily focused on the AF external surface during the morphing process, while (Du et al., 2021) emphasized the importance of accurately modeling the IVD’s internal structures, such as the nucleus, for improved precisions of the simulated mechanical responses. Early FE explorations have suggested that the specific configuration of these internal components impacts the prediction of local mechanical fields while not significantly affecting the overall flexibility of the organ (Noailly et al., 2007). Consequently, the literature may not fully grasp the importance of personalizing internal components such as the NP. Likewise, the possible importance of the multiplicity of local morphological features might have been neglected, as overall organ measurements like mid-height or coronal and sagittal distances have been traditionally explored (Urquhart et al., 2014; Teichtahl et al., 2015; Bach et al., 2019; Kizilgöz and Ulusoy, 2019), potentially neglecting the non-linear interactions between IVD phenotypes and mechanics.

Another significant impediment is the variation in tissue-level FE meshes across different studies. These discrepancies in mesh topologies often hinder the automation of simulations, large-scale result production, replication of results, clinical applicability, and multi-scale strategies using PP models. Ruiz et al. (2013) attempted to standardize an IVD mesh for the L4-L5 spinal segment to minimize result fluctuations, especially in the transition zone (TZ) between the NP and AF. However, this approach falls short in evaluating CEP diffusion distances due to using a simplified generic model.

This research aims to discern the influence of disc local morphologies on the biomechanical responses of the IVD and to provide a comprehensive free-access repository of PP IVD model geometries, underscoring our dedication to standardization and furthering the scientific understanding of IDD. We employed an advanced mesh morphing technique to adapt a previously calibrated, validated FE mesh (Ruiz et al., 2013) to PP IVD models, addressing the challenge of standardizing IVD meshes. These adaptations enable the execution of FE simulations under average daily physiological loads (Wilke et al., 1999), evaluating the effects of morphological factors on the multiphysics response. Our methodology incorporates a biphasic swelling model implemented in a User MATerial (UMAT) subroutine in ABAQUS 2020, allowing exploring the porous media of the IVD (Ruiz et al., 2018) and maintaining a consistent topology. Machine learning regression algorithms were used to refine our understanding of the relationship between IVD morphology and mechanical responses within targeted regions of interest.

### 1.1 Contributions of the work

• An automatic mesh morphing procedure was established to transform a structured IVD FE mesh, previously calibrated (Ruiz et al., 2013), into IVD PP models based on segmented medical images.
• The model was validated using experimental data from the literature.
• The interplay between morphology and mechanical responses was revealed through tissue-level simulations and machine-learning algorithms.
• A repository of 169 PP FE models of the IVD was created for the scientific community. Available for free use at the Zenodo open repository (Muñoz-Moya et al., 2023), accessible through our online user interface (https://ivd.spineview.upf.edu/).

## 2 Materials and methods

### 2.1 Overview

A cohort of 169 geometrical models of the lumbar spine IVD (Pfirrman scale range from 1 to 4) was acquired through T2-weighted magnetic resonance imaging (MRI) from the former project *MySpine* (FP7-269909) to generate the morphed FE meshes. These models were developed following the innovative approach introduced by Castro-Mateos et al. (2014), which enables accurate 3D segmentation of IVD annulus and nucleus, with increased resolution as axial and transversal MRI slices (isotropic voxels of 0.68 × 0.68 × 0.68 mm^3^) are combined. This method is effective for all degrees of IDD, including discs with protrusions or herniations. The segmentation algorithm, employing a feature selector, iteratively deforms an initial shape, which is projected into a statistical shape model space at first and then into a B-Spline space to improve accuracy. Expert clinicians validated the initial manual segmentation and the generated 3D morphologies.

**FIGURE 1:**
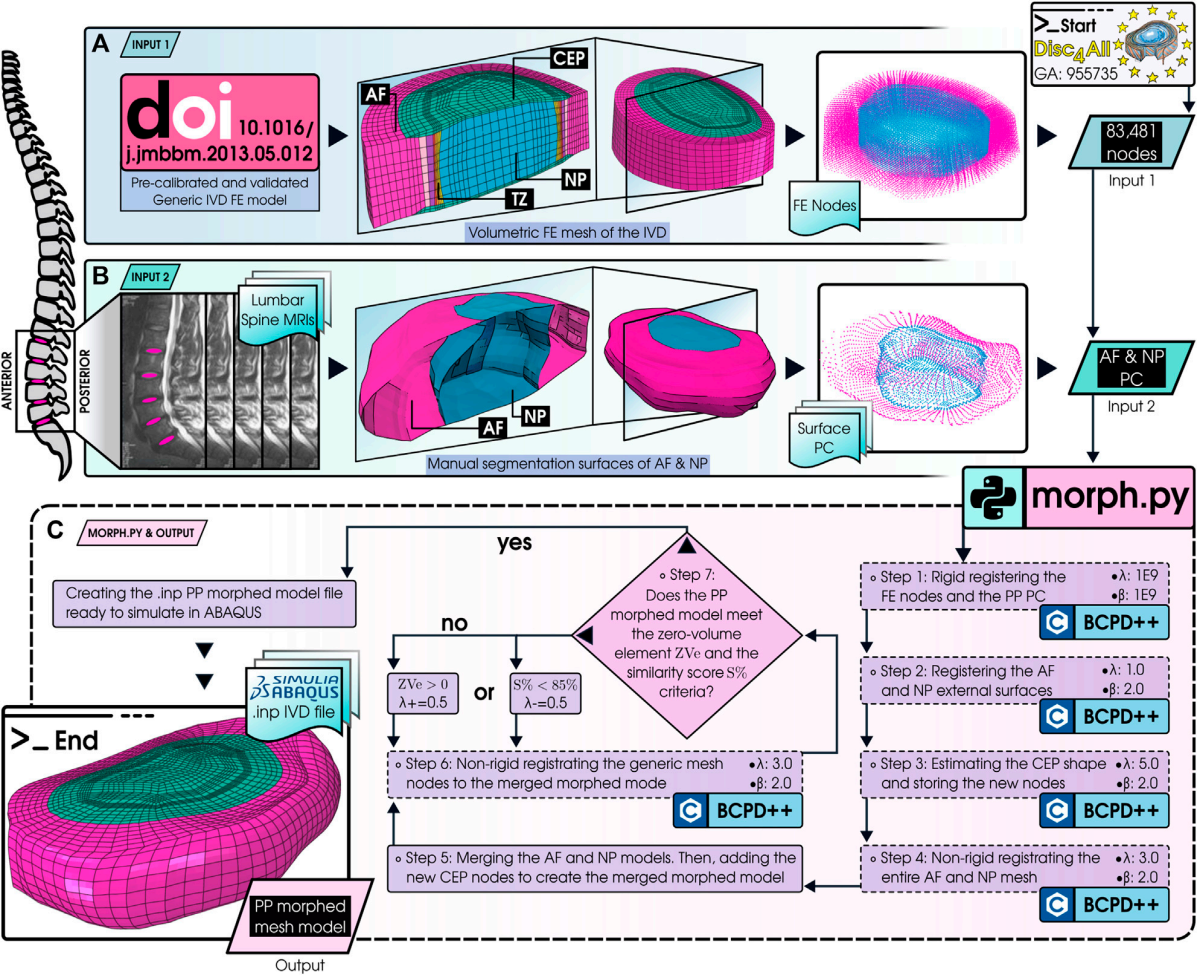
Schematic representation of the morphing process. **(A)** INPUT 1: The generic FE mesh of the IVD from Ruiz et al. (2013). Nodes from this mesh serve as the primary input for the morphing. **(B)** INPUT 2: A collection of 169 PCs showcasing the outer surfaces of the AF and NP. These were derived from PP MRIs and are incorporated into the morphing process (from the MySpine project). **(C)** MORPH.PY & OUTPUT: A detailed seven-step procedure generates the ABAQUS input file via the morph.py code and the BCPD++ algorithm (Hirose, 2021a). AF, Annulus Fibrosus; NP, Nucleus Pulposus; CEP, Cartilage Endplate; TZ, Transition Zone; FE, Finite Element; PC, Point Cloud; PP, Patient-Personalized; Zve, Zero-Volume Element; S%, Similarity Score. Parameters λ and β dictate the displacement vector’s expected length and directional correlation, respectively.

The point cloud (PC) of the AF and NP external surfaces could be extracted from the MRIs, but the segmented models did not incorporate the CEP (Figure 1B). To overcome this limitation, our morphing process estimates the shape of the CEP (presented in Figure 1C). This algorithm tailors an IVD FE generic mesh (previously calibrated through a comprehensive mesh convergence analysis (Ruiz et al., 2013), and originally developed by Noailly et al. (2010), presented in Figure 1A) to the PC of the PP models while preserving the relative dimensions of the elements and the mesh structure at material discontinuities. The entire mesh of the IVD contains 83,481 nodes, and the disc tissues—namely AF, NP, and CEP—were discretized with 19,392 second-order hexahedral elements (20 nodes).

The collagen fibers of the AF were modeled with 7,680 second-order quadrilateral rebar elements (8 nodes) embedded in the hexahedral elements of the annulus to model the fiber-reinforcement of the tissue. These rebars follow the concentric mesh structure of the annulus hexahedral elements to represent the functional mechanical behavior of the annulus structure within the IVD (Noailly et al., 2010).

The primary purpose of the pipeline presented in Figure 1 is to create a FE PP mesh file (.inp) compatible with the ABAQUS mechanical solver, with a mesh structure able to preserve simulation convergence and minimize poro-mechanical instabilities under physiological loading conditions (Ruiz et al., 2013). Furthermore, our algorithm guarantees the non-convexity of the elements, contributing to the simulations’ accuracy.

The simulations adopt the same boundary conditions, material properties, and physiological loads used by Ruiz et al. (2018) for healthy discs, based on *in vivo* intradiscal pressure measurements by Wilke et al. (1999). Each PP FE model has the same number of elements and nodes and the same connectivity. The sole difference among the PP models lies in the coordinates of the nodes within the FE mesh.

To validate our model’s capacity to represent the mechanical behavior of a normal healthy disc, we compared our simulations with the *in vitro* vertical creep displacement tests by Malandrino et al. (2015b). We employ the morphing algorithm to obtain the morphology of the IVD used in these experiments and consider its effects on the validation simulation.

Thanks to the consistent topology across the 169 PP morphed models, extracting mechanical responses from targeted zones, such as the NP’s center, the anterior and posterior TZs (regions prone to early degeneration, observed by (Smith et al., 2011) through clinical images), and the CEP—was straightforward and systematic for all models. Accordingly, local mechanical predictions could consistently correlate with various morphological factors, such as heights, areas, and volumes. The relevance of each factor was then ranked by leveraging SHAP (SHapley Additive exPlanations) values (Ning et al., 2022).

### 2.2 Morphing process

The Bayesian Coherent Point Drift ++ (BCPD++) (Hirose, 2021a; Hirose, 2021b) was employed to adapt the structured generic IVD FE mesh (Ruiz et al., 2013) to the PP point cloud (AF and NP). This Bayesian framework allows the inclusion of prior knowledge about the distribution of potential transformations, such as degrees of translation, rotation, and scaling among the points. Overall, the BCPD++ algorithm was chosen to facilitate the automation of transforming FE meshes.

• Algorithm convergence is guaranteed by variational Bayesian inference while introducing motion coherence using a prior distribution of displacement vectors.
• Rigid and non-rigid registration can be executed within a single algorithm.
• The algorithm works with both structured and unstructured shapes.
• Point-to-point correspondences are not assumed to be one-to-one.

To adapt the mesh to the PP models and estimate the CEP shape, the BCPD++ algorithm was regulated through our in-house Python script named morph.py (Figure 1C). Additionally, the script substitutes the original hexahedral elements of the BEP (Noailly et al., 2007; Malandrino et al., 2011; Ruiz et al., 2013) from the generic mesh with 3,552 eight-node shell elements in the top and bottom regions of the disc, consequently reducing the computation time of both the morphing and the FE simulations.

In brief, the algorithm has two main parameters that were controlled hereby: *λ*, which controls the expected length of the displacement vectors, and *β*, which controls the directional correlation among the displacement vectors. *λ* and *β* facilitate the balance of rigid and non-rigid registrations by using large or small values of each parameter, which enables the control of the distortion of the deformation field.

To accurately estimate the CEP, it is crucial to set a specific target thickness range to ensure that modeled healthy discs do not fall below the expected thickness values. Moon et al. (2013) found, via MRI observations, that the central thickness of a healthy disc—considered the minimum thickness across the entire CEP—measured in the sagittal plane averages 0.54 ± 0.12 mm across all lumbar levels. This finding is consistent with our generic model, which specifies a CEP thickness at the center of the disc of 0.545 mm, initially based on histological measurements (Noailly et al., 2007). Consequently, ensuring that the average CEP thickness of the discs generated by the morphing process falls within this range becomes a key objective, particularly for discs not in advanced degeneration stages. Additionally, focusing on the central thickness allows for the creation of CEPs with variable thickness across the tissue, adapting its shape to match the morphology of the AF and NP, thereby introducing a more realistic and nuanced approach to modeling healthy and degenerated discs.

The process of creating the PP models is divided into seven key steps, presented in Figure 1C.

• **Step 1:** Initial Alignment—The IVD generic FE mesh and the PP point cloud are aligned using a rigid registration process. This step employs a theoretically infinitely large value for both *λ* and *β* to achieve a perfect initial alignment without any deformation of the models. Here, *λ* = 1E9 and *β* = 1E9 are suggested by Hirose (2021b) to represent the infinity.
• **Step 2:** Surface Adaptation—A Non-rigid registration of the external surface nodes of the generic FE mesh (source) to the external surface point clouds of the PP geometrical model (target) is conducted to replicate the external surfaces of both the AF and NP. This ensures that the PP model surfaces include the structured topology of the FE mesh. Here, *λ* = 1.0 and *β* = 2.0 are used.
• **Step 3:** CEP Thickness Control—This step repeats the non-rigid registration process of Step 2, but this time, the source includes the volumetric nodes of the CEPs within the generic mesh (top and bottom CEP of the disc). The registration targets the points of the PP geometrical model’s AF and NP external surfaces. A higher *λ* than in Step 2 prevents the distortion of the CEP node cloud: it keeps the NP interfacing nodes of the CEP on the same plane as the external surface of the NP while simultaneously aligning the CEP outer nodes with the neighboring AF outer surface, thus avoiding the creation of zero-volume elements. In this way, the CEP was generated between the NP and AF, allowing us to estimate the real position of CEP, even without direct tissue segmentation. The new nodal coordinates of the CEP are retained for later use in Step 5. The values used are *λ* = 5.0 and *β* = 2.0. Notably, the thickness of the CEP can be dynamically adjusted by varying *λ*: increasing *λ* enhances the thickness, whereas decreasing it reduces the thickness. However, this causes part of the NP volume to be used to increase the thickness of the CEP.
• **Step 4:** Volumetric Mesh Morphing—The volumetric AF and the NP generic meshes are non-rigidly registered as sources to the morphed outer surface (obtained in step 2) as a target. This morphing is performed without the CEP nodes (estimated in step 3). This allows the volumetric mesh of the AF and NP to be obtained with the external shape provided by the PP model. Here, *λ* = 3.0 and *β* = 2.0 are used.
• **Step 5:** Model Merging—The AF and NP volumetric meshes are integrated into a single merged morphed model. The transition zone nodes (at the AF and NP boundary) maintain the NP shape. The CEP nodes (estimated in step 3) are then added. However, since the registration processes are carried out separately, the model may have overlapping nodes, which is addressed in step 6.
• **Step 6:** Final Mesh Registration—All nodes of the FE mesh (including AF, NP, CEP, and TZ) are non-rigidly registered to the merged morphed model (obtained in Step 5). Since the source and the target now have the same number of points, locating their corresponding nodes becomes straightforward. Moreover, BCPD++ maintains the proportion of the relative distances of the source nodes to be deformed and adapted to the PP model, aiding in maintaining the original FE model’s mesh quality. Finally, the morph.py script creates an ABAQUS.inp file with the same boundary conditions, material properties, and physiological loads as used by Ruiz et al. (2018). The values used are *λ* = 3.0 and *β* = 2.0.
• **Step 7:** Quality Evaluation—Two criteria were employed to evaluate the procedure’s quality:
  1. Zero-Volume Element Check (ZVe): The initial step involves inspecting the ABAQUS.inp file for zero-volume elements. If such elements are detected, the process iterates Step 6 again with an increased (+0.5) *λ* value. This adjustment aims to minimize deformation in the relative distances between nodes, enhancing the mesh integrity.
  2. Similarity Score (*S%*): The absence of zero-volume elements leads to the second evaluation criterion, which utilizes the Hausdorff distance (HD) to measure the similarity between the original model and the morphed model. The process begins by establishing a Hausdorff distance reference value of error. This reference value is calculated between the original model and a version of the model enlarged by 10% (achieved by scaling each coordinate by 1.1), which is assumed to represent an error percentage (*e%*) of 10% to the original. Next, the Hausdorff distance between the original model and the morphed version thereof is determined, and the corresponding error is calculated through a cross-multiplication, assuming direct proportionality with the 10% of error as the baseline. Finally, the similarity (*S%*) is calculated by subtracting the error to 100%. The target is to achieve at least an 85% similarity score. If this target is not met, the *λ* value used in Step 6 is lowered (−0.5) to achieve a closer match in subsequent iterations. The Hausdorff distance is sensitive to outliers: as it focuses on the maximum distance between the respective points of the two sets to be compared, a single point can disproportionately affect the similarity score. Therefore, the median is used for every comparison to reduce the effect of any extreme discrepancies that might exist only at a few points in the models. Thus, the similarity score *S%* is formulated as:

$$\begin{array}{cc} e\% & =\frac{{\text{HD}}_{\text{median}}\left(\text{original},\text{morphed}\right)}{{\text{HD}}_{\text{median}}\left(\text{original},\text{enlarged}\right)}\times 10\%\\ S\% & =100\%-e\% \end{array}$$ (1)

Where HD_median_ (original, enlarged) represent the median Hausdorff distance between the original model and its version enlarged by 10%. HD_median_ (original, morphed) denote the median Hausdorff distance between the original model and the morphed model.

3. Exception: If there is any morphed model that presents at the same time zero-volume elements and a similarity score lower than 85% (ZVe >0 & S% < 85%), then the model is discarded.

Once all the IVD morphed models were prepared, the mesh quality was contrasted with the generic FE mesh of Ruiz et al. (2013). This allowed us to assess whether the BCPD++ algorithm could maintain the proportionality of the nodes’ relative distances without inducing excessive deformation.

Each BCPD++ process was accelerated inside and outside the variational Bayes inference using Nystrom’s method alongside KD-tree search. Furthermore, a downsampling strategy was implemented to manage the number of model points, standardizing on a voxel size of 0.1 across all instances. This approach to acceleration and downsampling adheres to the default parameters as suggested by Hirose (2021b).

### 2.3 Constitutive modeling of the IVD

The generic mesh of the IVD, including the morphed models (healthy and degenerated disc geometries), uses the same constitutive model, adapted by Ruiz et al. (2018) and Noailly et al. (2010). This model was implemented within an ABAQUS UMAT subroutine, allowing the biomechanical parameters to accurately reflect the properties of healthy disc tissues (Ruiz et al., 2016; Ruiz et al., 2018). This model primarily aims to isolate and examine the effects of disc morphology on biomechanical behavior, ensuring that the constitutive properties remain consistent across healthy and degenerated disc geometries.

The IVD material model considers 1) a solid phase comprising structural macromolecules such as collagen, elastin, and proteoglycans, alongside cells and 2) a fluid phase consisting of water and solutes (Malandrino et al., 2015a). The biphasic-swelling (BS) theory, as detailed by Mow et al. (1980), Mow et al. (1989), delineates both the equilibrium and transient mechanics of charged soft tissues in IVDs. This theory presents each tissue as a composite material featuring a charged solid porous phase saturated by interstitial fluid, thereby enabling the simulation of fluid pressurization and movement within the disc.

This study characterizes the behavior of the entire disc through an osmo-poro-hyper-viscoelastic model. This comprehensive model integrates the constitutive tissue of the bony endplate, treated as a linear poroelastic material (Malandrino et al., 2011) through shell elements. Furthermore, the annulus, nucleus, cartilage, and transition zone, represented with second-order hexahedral elements, employ the BS model used to simulate poromechanical interactions within a poro-hyperelastic matrix saturated with intra- and extra-fibrillar fluid (Wilson et al., 2005b), including the Donnan osmotic pressure gradient effects (Urban and Maroudas, 1981). In addition, the model considers viscoelastic collagen fibers present only in the AF Wilson et al. (2006a) as rebar elements.

The total stress tensor ***σ***_tot_ is expressed as the superimposition of the effective stress ***σ***_eff_ (defined in Section 2.3.1) of the solid skeleton within the pores, a fluid pore pressure component *p*, and Darcy’s law:

$$\sigma_{\text{tot}}=\sigma_{\text{eff}}-pI$$ (2)

q=κ∇p (3)

Where ***I*** is the identity tensor, ***q*** is the fluid mass flow to the spatial gradient of pore pressure ∇*p*, and ***κ*** is the hydraulic permeability tensor of the tissue. Also, the fluid flow can be expressed by:

$$q=u_{f}n_{f}$$ (4)

Where ***u***_*f*_ is the pore fluid velocity, and *n*_*f*_ represents the total water fraction, i.e., the porosity of the medium.

Due to the fixed charges, the cation concentration inside the tissue is higher than in the surrounding body fluid (Wilson et al., 2005b). This excess of ion particles within the matrix creates the Donnan osmotic pressure, Δ*π*, which drives the fluid flow, causing the swelling of the tissue (Urban et al., 1979). Incorporating the osmotic pressure into Eq. 2, where Schroeder et al. (2007) adapted this equation to the IVD, the hydrostatic fluid pressure *p* is defined as:

$$p=u_{w}+\Delta \pi$$ (5)

*u*_*w*_ is the water chemical potential, linked with the pore pressure degree-of-freedom generated by the interstitial fluid permeation effects through the permeability (introduced in Section 2.3.5) by applying Darcy’s law to describe a relationship between fluid flow and the swelling pressure. Therefore, fluid flow between the different tissues of the model depends on the tissue-specific mappings of permeability. Δ*π* represents the osmotic pressure gradient generated by the difference between the internal and external salt concentrations (more details in Section 2.3.3).

#### 2.3.1 Solid matrix—non-fibrillar part

The macroscopic stress-strain response of the solid matrix is determined by the initial shear modulus, *G*_*m*_, the initial (in the unloaded and non-swollen state) volume fraction, *n*_*s*,0_, and the current deformations of the homogenized poroelastic continuum. This response follows the Cauchy stress of the non-fibrillar matrix to describe the material’s finite strain behavior, as Wilson et al. (2005a), Wilson et al. (2005b), Wilson et al. (2006a), Wilson et al. (2006b) detailed initially and then adapted by Schroeder et al. (2008) for the IVD:

$$\sigma_{\text{eff}}=-\frac{1}{6}\frac{\ln\left(J\right)}{J}G_{m}I_{1}\left[-1+\frac{3\left(J+n_{s,0}\right)}{\left(-J+n_{s,0}\right)}+\frac{3J\ln\left(J\right)n_{s,0}}{{\left(-J+n_{s,0}\right)}^{2}}\right]+\frac{G_{m}}{J}\left(B-J^{\frac{2}{3}}I_{1}\right)$$ (6)

Where *J* is the determinant of the deformation gradient tensor ***F***, and ***I***_1_ is the first invariant of the left Cauchy–Green strain tensor ***B*** = ***F*** ⋅ ***F*** ^⊺^.

#### 2.3.2 Annulus fibrosus collagen fibers—fibrillar part

In the AF, collagen fibers exhibit a unidirectional viscoelastic mechanical response. This behavior is modeled by incorporating finite strains in a Zener viscoelastic model with two non-linear springs. Assuming that the fibrils only resist tension, the Cauchy fibril stress tensor in a unit area for viscoelastic fibrils (Wilson et al., 2006a; Wilson et al., 2006b) can be expressed as:

$$\sigma_{\text{f}}=\frac{\psi }{J}P_{f}{\vec{e}}_{f}{\vec{e}}_{f}$$ (7)

Where *ψ* is the elongation of the fibril, *P*_*f*_ is the first Piola-Kirchhoff fibril stress, and ${\vec{e}}_{f}$ is the current fibril direction.

#### 2.3.3 Pressure component—osmotic swelling

The Donan osmotic potential describes swelling behavior (Malandrino et al., 2015a), assuming that electrolyte flux can be neglected in mechanical studies of charged materials. Accordingly, the internal and external osmotic pressures are represented by the classical Van’t Hoff equation (Huyghe and Janssen, 1997), and assuming that the osmotic components are instantaneously equilibrated with the external bath, the osmotic pressure gradient Δ*π* is given by (Wilson et al., 2005b):

$$\Delta \pi =\phi_{\text{int}}RT\left(\sqrt{c_{f,\text{exf}}^{2}+4{\left(\frac{\gamma_{\text{ext}}^{\pm }}{\gamma_{\text{int}}^{\pm }}\right)}^{2}c_{\text{ext}}^{2}}\right)-2\phi_{\text{ext}}RTc_{\text{ext}}$$ (8)

Where *R* is the gas constant, and *T* is the absolute temperature. The internal and external osmotic coefficients *ϕ*_int_ and *ϕ*_ext_ multiply the terms related to concentrations of mobile cations and anions, respectively. The average of the internal and external activity coefficients of the ions is represented by $\gamma_{\text{int}}^{\pm }$ and $\gamma_{\text{ext}}^{\pm }$ (assuming $\gamma^{\pm }=\sqrt{\gamma^{+}\gamma^{-}}$ ). These osmotic and activity coefficients were implemented as Huyghe and Janssen (1997); Huyghe et al. (2003) proposed and as other authors such as (Wilson et al., 2005b; Schroeder et al., 2007) and Galbusera et al. (2011) adopted in their respective studies. The external concentration of salt and the proteoglycan fixed charge density are denoted by *c*_ext_ and *c*_*f*,exf_, respectively.

#### 2.3.4 Tissue model parameters and relation to composition measurements

To elucidate the relationship between biphasic/poroelastic models and IVD deformations quantified by *J*, it’s essential to connect the variables in the equations for the non-fibrillar solid matrix’s effective stress (Eq. 6) and the osmotic potential (Eq. 8). The proteoglycan fixed charge density is determined by the ratio of the normal fixed charge density (*c*_*f*_) in milliequivalents per milliliter of total fluid to the extra-fibrillar water (*n*_*f*,exf_), as defined by Ruiz et al. (2016):

$$c_{f,\text{exf}}=\frac{n_{f}c_{f}}{n_{f,\text{exf}}}$$ (9)

Where *n*_*f*,exf_ is derived as:

$$n_{f,\text{exf}}=n_{f}-\varphi_{ci}\rho_{c,\text{tot}}$$ (10)

In this Equation, *φ*_*ci*_ indicates the intrafibrillar water content per unit mass of collagen, and *ρ*_*c*,tot_ signifies the total collagen content as a proportion of the tissue’s total wet weight (WW).

To ascertain water content, the initial step involves measuring the tissue sample’s wet weight (WW) (Huyghe et al., 2003; Malandrino et al., 2015a; Ruiz et al., 2016), followed by lyophilization to obtain the dry weight (DW). These measurements facilitate the calculation of the initial total water content (*n*_*f*,0_) and, subsequently, the initial solid fraction and the current fluid fraction:

$$n_{f,0}=\frac{\text{WW}-\text{DW}}{\text{WW}}$$ (11)

$$n_{s,0}=1-n_{f,0}$$ (12)

$$n_{f}=\frac{n_{f,0}-1+J}{J}$$ (13)

Thus, using *n*_*f*,0_ as a foundational value, the equations seamlessly connect *n*_*s*,0_ and *n*_*f*_ with the IVD deformations represented by *J*, establishing a coherent framework for relating IVD composition measurements to mechanical modeling parameters. Then, the total fluid volume ratio is calculated using the void volume in the medium (d*V*_*v*_) and the total volume of the medium (d*V*):

$$n_{w}=\frac{dV_{v}}{dV}$$ (14)

To estimate the proteoglycan and total collagen contents, previous works propose digesting the dried samples in a papain solution. The digesteds solutions were then used (i) to determine the content of sulfated glycosaminoglycans (sGAG) through a dimethyl methylene blue (DMMB) assay (Farndale et al., 1986) and (ii) to achieve a measure for collagen content according to hydroxyproline measurements through the chloramine-T assay (Huszar et al., 1980).

We calculated the initial fixed charge density (*c*_*f*,0_) per total hydrated tissue volume, from which *c*_*f*_ (Eq. 9) is derived to be dependent of *J*, using the expression (Narmoneva et al., 1999):

$$c_{f,0}=\frac{z_{cs}c_{cs}}{{\text{MW}}_{cs}}$$ (15)

$$c_{f}=c_{f,0}\frac{n_{f,0}}{n_{f,0}-1+J}$$ (16)

Here, *z*_*cs*_, MW_*cs*_, and *c*_*cs*_ are the valency (2 mEq/mmol), the molecular weight (513,000 *μ*g/mmol), and the concentration (in *μ*g/mL) of chondroitin sulfate, respectively. The sGAG content measured through the DMMB assay is assumed to be equivalent to the chondroitin sulfate content, i.e., *c*_*cs*_ is the amount of sGAG divided by the sample’s water content. To obtain *ρ*_*c*,*tot*_, in Eq. 17, the initial collagen content (*μ*g/mg DW) was estimated from hydroxyproline content by using 7.6 as the mass ratio of collagen to hydroxyproline (Sivan et al., 2006):

$$\rho_{c,tot}=\%\text{hydroxyproline}\cdot 7.6$$ (17)

#### 2.3.5 Permeability

The tissue’s AF and NP hydraulic permeability (*κ*) are strain-dependent according to the following expression as Wilson et al. (2006a) developed and then adapted by (Schroeder et al., 2007) for the IVD:

$$\kappa =\alpha {\left(1-n_{f,\text{exf}}\right)}^{-M}$$ (18)

Where *α* stands for the initial permeability at zero strain, and *M* is a positive constant that governs volumetric strain dependency, and *n*_*f*,exf_ is calculated in Eq. 10.

The CEP is also strain-dependent, following this equation (Mow et al., 1980; Argoubi and Shirazi-Adl, 1996):

$$\kappa =\alpha {\left[\frac{e{\left(1+e_{0}\right)}^{2}}{e_{0}\left(1+e\right)}\right]}^{2}\exp\left[M\left(\frac{1+e}{1+e_{0}}-1\right)\right]$$ (19)

Where *e* is the void ratio, which is the ratio of the current pore volume (i.e., fluid) to the current volume of the solid matrix, and *e*_0_ is the initial void ratio. The void ratio is related to the initial and current water content/porosity of the tissue, *n*_*f*,0_ and *n*_*f*_, according to the following expression:

$$e=\frac{n_{f}}{1-n_{f}}\;\text{and}\;e_{0}=\frac{n_{f,0}}{1-n_{f,0}}$$ (20)

All tissue compositions for both non-degenerated and degenerated IVD were considered for a Grade I IVD and were taken from the literature (Table 1). All mechanical behavior has been detailed in the Supplementary Section S1. See the works of Huyghe et al. (2003); Malandrino et al. (2015a); Ruiz et al. (2013), Ruiz et al. (2016), Ruiz et al. (2018) for more information on the evolution of the used model.

**TABLE 1:** Material properties used for all the different disc model morphologies.

Parameters		Tissue		
		AF	NP	CEP
*G*	MPa	0.84	1.0	1.0
*c*_*f*,0_* → *c*_*f*,0_	% mEq/mL	18 → 20	29 → 30	16.6 → 17
*n*_f_* → *n*_*f*,0_	% WW	70.4 → 75	74.5 → 80	58.9 → 66
*ρ*_*c*,tot_	% DW	65	15	24
*c*_ext_	mEq/mL	0.15	0.15	0.15
*α*	mm^4^ /Ns	1.6 × 10^−4^	1.6 × 10^−4^	1.7 × 10^−2^
*M*	—	1.2	1.2	1.2

*G*: Shear modulus, *c*_*f*,0_: Initial fixed charge density, *n*_*f*,0_: Initial fraction of water, *ρ*_*c*,tot_: Initial collagen content concerning the total dry weight, *c*_ext_: External concentration of salt, *α*: Initial permeability at zero strain, *M*: Positive constant that governs volumetric strain dependency, DW: Dry weight, and WW: Wet weight. (*): Values at the beginning of the swelling step. *G* of AF is from Ruiz et al. (2016), contributing the 84%, and 16% for fibers. *α* of CEP was back-calculated (Ruiz et al., 2018) and then validated against the experimental study of Accadbled et al. (2008). *G* of NP and CEP is for the non-fibrillar matrix. These values, with the rest of all tissue’s material compositions, are from the work of Ruiz et al. (2018).

### 2.4 Mechanical simulations

This simulation was conducted following a free swelling step of 17 h (as per the BS theory outlined in Section 2.3.3). No external loads were applied on the morphed IVD to simulate free swelling, which stood for an initialization of the expected equilibrium osmotic pressurization of the disc, according to the Eq. 8. The pre-swelling initial values for fixed charge density and water content, denoted by *c*_f*_ and *n*_f*_ in Table 1, were set to gradually reach the *c*_*f*,0_ and *n*_*f*,0_ values proposed in the literature by the end of the swelling process (Ruiz et al., 2018).

**FIGURE 2:**
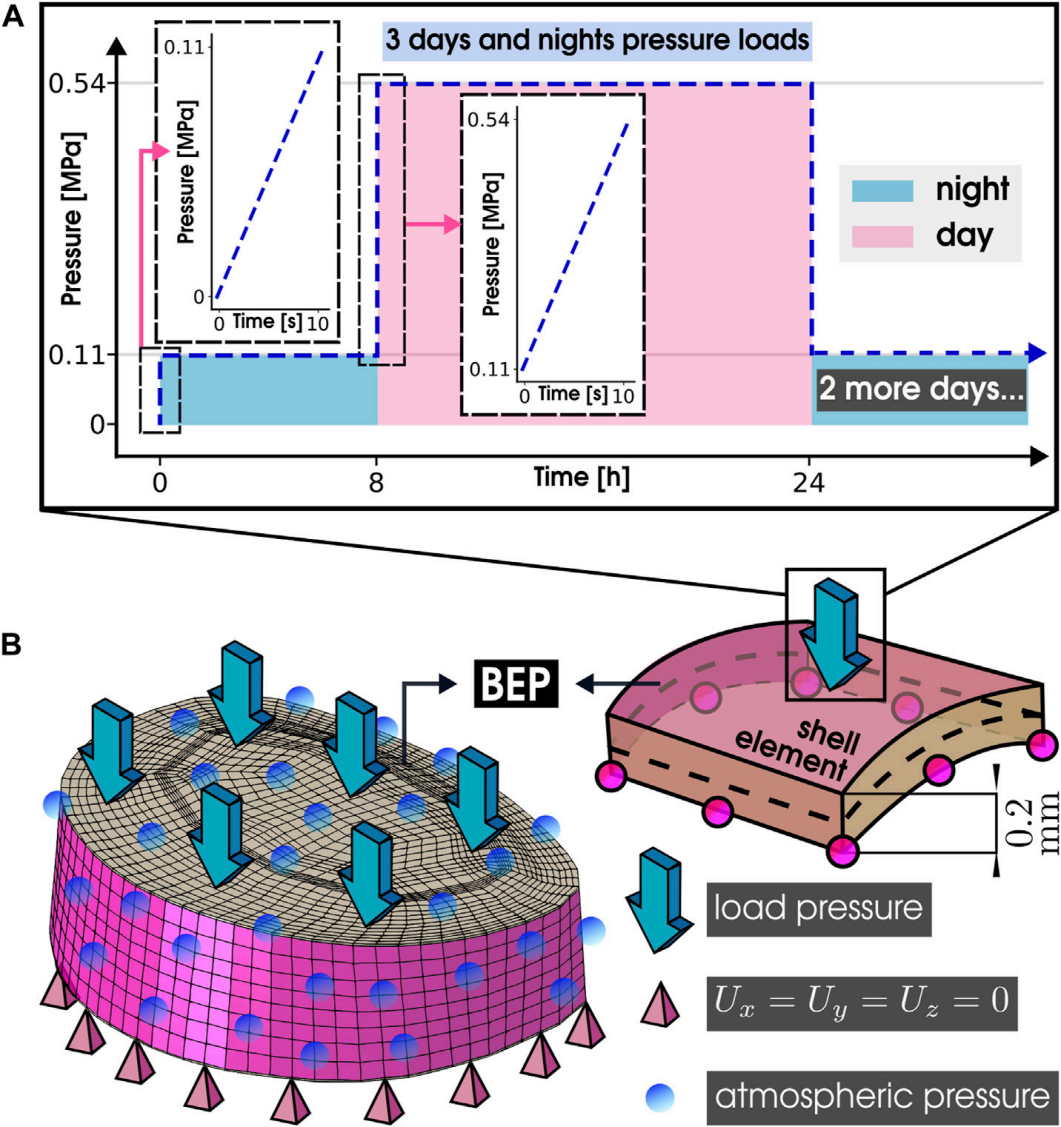
Simulation specifications. **(A)** Applied pressure on the IVD, Average activity and resting intradiscal pressures measured *in vivo* by Wilke et al. (1999). **(B)** Boundary conditions of the simulation: Load pressure, fixed displacement, and atmospheric pressure conditions on the IVD. BEP, Bony Endplate.

Three daily load cycles were then simulated to identify which morphological factors significantly affected the mechanical response during average human activities. Accordingly, each cycle started with an 8-h resting period (creep step) under 0.11 MPa compression (load step of 10 s), which simulated overnight best rest. It was followed by 16 h of average day activity (creep step) under a load of 0.54 MPa compression (load step of 10 s), as illustrated in Figure 2A. These load values were selected based on average activity and resting intradiscal pressures as measured *in vivo* by Wilke et al. (1999), as proposed by Ruiz et al. (2016). The compressive loads were applied to the BEP shell elements (as mentioned in Section 2.2) of the top, while the nodes of the caudal BEP remained fully constrained (*U*_*x*_ = *U*_*y*_ = *U*_*z*_ = 0). The simulation also accounted for atmospheric external pressure (Figure 2B).

Given the incorporation of swelling into our simulations, it is necessary to detect potential deviations between the real geometry from the MRI models and the geometry following the morphing process, which includes swelling. To address this, we selected three IVD models with varying mid-heights (MH) for detailed analysis (See Table 2). Post-swelling, these were used to re-evaluate the Similarity Score (refer to Section 2.2 for details).

**TABLE 2:** Morphed IVD Models used to check similarity score post-swelling. Mid-height: MH.

ID	Level	MH [mm]	SpineView link
MY0092	L4-L5	8.04	https://ivd.spineview.upf.edu/?filenamePrefix=MY0092_L4L5
MY0002	L5-S1	14.20	https://ivd.spineview.upf.edu/?filenamePrefix=MY0002_L5S1
MY0065	L3-L4	17.41	https://ivd.spineview.upf.edu/?filenamePrefix=MY0065_L3L4

## 3 Model validation

The constitutive model and the morphing process were validated using the experimental tests reported by Malandrino et al. (2015b), performed with a 500 N sustained compressive load, similar to a physiological upper body standing weight of 50 kg (Heuer et al., 2007; Ruiz et al., 2016; Hassan et al., 2020), which can produce fluid loss from the IVD and height reduction (Adams and Hutton, 1983). In his works, creep compression tests were briefly done on L3-L4 segments extracted from four lumbar spines. The IVDs were placed in a neutral position in the spine tester (WISI) of the Institute of Orthopedic Research and Biomechanics (Ulm, Germany) (Wilke et al., 1994), with their mid-transverse planes normal to the vertical direction. Only vertical, i.e., axial displacements, were permitted during the loading period. An initial compressive preload of 300 N was applied for 180 s and then withdrawn for 180 s. This cycle was repeated three times to precondition the IVD. Subsequently, the load was increased to 500 N in 10 s and sustained for 3 h, with creep responses recorded as displacements.

We selected an IVD exemplifying healthy disc characteristics to align with the mechanical properties defined in our model. This selection was followed by the morphing process detailed in Section 2.2, aimed at replicating the morphology reported in the study of Malandrino et al. (2015b) (height of 12.1 mm, sagittal distance of 37.6 mm, and coronal distance of 47.4 mm). We conducted a comparative analysis to ascertain our constitutive model’s accuracy and the morphing process’s effectiveness. This involved contrasting the displacement of the disc’s top zone as measured in both the experimental setup and the simulation outcomes of the referenced study against the results from our simulation.

## 4 Data mining

To explain the connection between morphological attributes and the early stages of disc degeneration across our 169 IVD PP FE model dataset, we analyzed the mechanical simulation outcomes as detailed in Section 2.4. This analysis was strategically focused on five distinct zones of interest, chosen for their critical relevance to the onset of disc degeneration. We analyzed morphological factors based on their significance in the qualitative assessment of Pfirrmann grading and their visibility in sagittal and coronal clinical images. Concurrently, we concentrated on mechanical variables that are pivotal for understanding stresses and the mechanisms of indirect mechanotransduction.

For the predictive analysis, we employed three regression models: Linear Regression (LR), Support Vector Machine Regression (SVR), and Extreme Gradient Boosting Regression (XGBoostR). Each model was trained according to the morphological factors of the morphed IVDs, and we explored the relationship between morphological measurements and mechanical responses by using the SHAP (SHapley Additive exPlanations) values that ranked the impact of each factor in each regression model. The models’ inputs were the morphological features, and the targets were the mechanical responses.

The selection process for the optimal model for each mechanical variable was based on predictive performance, utilizing the R-squared (*r* ^2^) metric to identify the model with superior predictive power. Furthermore, the Mean Squared Error (MSE) was used to assess whether the chosen model would exhibit high predictive accuracy and keep minimal prediction error.

### 4.1 Zones of interest for evaluation

To discern possible mechanical changes because of the morphological variability in regions likely relevant to IDD pathophysiology, we focused on particular regions susceptible to being altered in early IDD. On one hand, the center of the nucleus pulposus is a natural candidate region, according to Pfirrmann’s grading. On the other hand, our mesh structure contains a region, the transition zone, that is worth exploring. This zone, as defined in our FE meshes, emerged out of a need for computational stability to ensure the FE mesh convergence and cope with the negative effects of weak discontinuities between the nucleus pulposus and the annulus fibrosus elements (Ruiz et al., 2013). Interestingly, though, a transition between the nucleus and the annulus exists, as revealed by several quantitative MRI, synchrotron imaging, cell phenotypes, and structural and composition measurements through the IVD (Marchand and Ahmed, 1990; Bruehlmann et al., 2002; Disney et al., 2023; Kapoor et al., 2023). Hence, the definition of this region in the FEM results in a more realistic description of the IVD, in contrast to an abrupt change of material properties from the NP to the AF.

**FIGURE 3:**
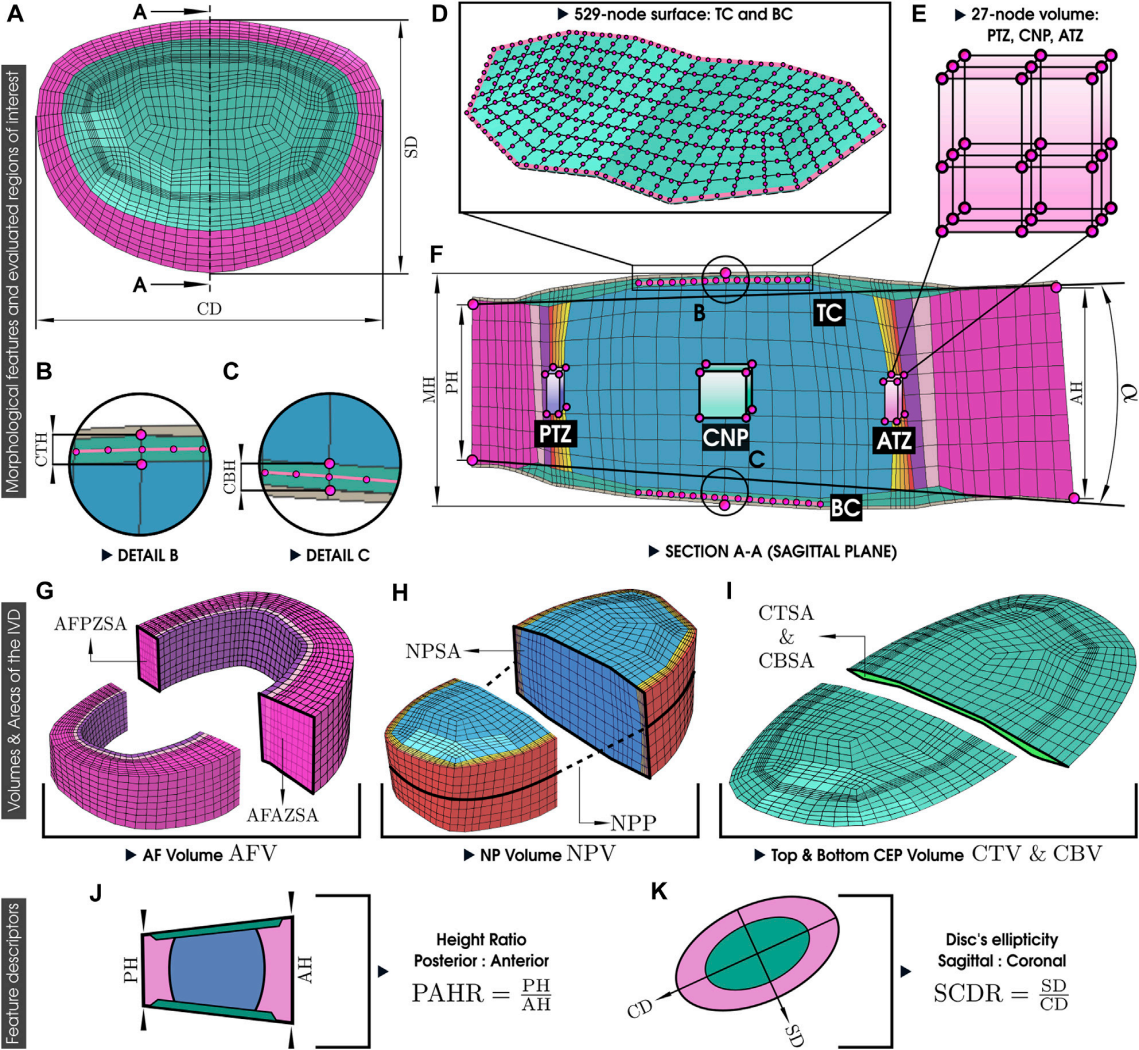
Morphological measurements of the disc. PH, Posterior Height; MH, Middle Height; AH, Anterior Height; CTH, Cartilage Endplate Top Height; CBH, Cartilage Endplate Bottom Height; PAHR, Posterior and Anterior Height Ratio; SD, Sagittal Distance; CD, Coronal Distance; SCDR, Sagittal and Coronal Distance Ratio; NPP, Nucleus Pulposus Perimeter; AFPZSA, and AFAZSA: Sagittal Area of both the Posterior Zone and Anterior Zone of Annulus Fibrosus; NPSA, Nucleus Pulposus Sagittal Area; CTSA, Cartilage Endplate Top Sagittal Area; CBSA, Cartilage Endplate Bottom Sagittal Area, AFV, NPV, CTV, and CBV, Volumes of Annulus Fibrosus, Nucleus Pulposus, and both Cartilage Endplates and *α*: Wedge Angle. The assessment of simulation results incorporated the regions of interest, the three 27-node volumes, and two 529-node surfaces. PTZ, Posterior Transition Zone; CNP, center of the Nucleus Pulposus; ATZ, Anterior Transition Zone; C, Top Cartilage Endplate; BT, Bottom Cartilage Endplate. **(A–K)** labels are explained in Table 3.

Therefore, our analysis focused on the finite element simulations of the local tissue mechanics in these corresponding volumes that show signs of alterations in early-degeneration stages positioned along the sagittal plane of the disc: the transition zones (Posterior Transition Zone, PTZ; Anterior Transition Zone, ATZ), and the Center of the Nucleus Pulposus (CNP). Furthermore, we analyzed the mechanical features calculated over the central regions of the top and bottom CEP surfaces (TC and BC, respectively) to underscore the potential significance of the CEP in different disc morphologies. For each volume (PTZ, CNP, ATZ), average mechanical responses were calculated over 27 nodes contained in the volume (Figure 3E). For each surface (TC and BC), average mechanical values were computed over 529 nodes belonging to the surface (Figure 3D).

The selection of 27 nodes within each volume is grounded on the definition of a minimal-size transition zone to avoid numerical instabilities in our generic model, as defined by Ruiz et al. (2013). The TZ, characterized by a composition of 5 second-order hexahedral elements in thickness, opts for an internal volume excluding the nodes at the nucleus or annulus interface. Given the second-order nature of the hexahedral elements, the thickness includes 11 nodes, with those at positions 3, 6, and 9 being specifically chosen. Adopting a 27-node volume for the CNP facilitates a more coherent comparison by leveraging the vertex nodes of the 8 central hexahedral elements within the nucleus. Meanwhile, the CEP surface selection aims to minimize the influence of the AF, focusing solely on its relation to the NP.

### 4.2 IVD model morphological features

Despite numerous studies using Pfirrmann’s classification to assess varying degrees of degeneration and morphological measurements, the specific morphological factors associated with IDD remain elusive. Previous studies, including observational and *ex vitro* research, have focused on the significance of disc height reduction at the posterior, middle, and anterior sections of IDD. These studies, however, have often presented inconclusive outcomes regarding the morphological attributes of IDD (Urquhart et al., 2014; Teichtahl et al., 2015; Bach et al., 2019; Kizilgöz and Ulusoy, 2019; Fleps et al., 2024; Tavana et al., 2024). Hence, it becomes necessary to explore the entire morphology of the discs, combining all the possible factors to understand the interplay between them.

As the morphological variations are reflected by different node coordinates in each model and the direct mechanical and tissue property environment of each region is kept consistent with the entire set of PP models, 20 morphological factors were extracted from finite element (FE) model meshes, with a consistent topology across all models. This enhances the precision of measuring heights and distances, both observable in the sagittal and coronal planes and measurable in clinical images, particularly in sagittal lumbar MRI. Further morphological features, such as the volumes, can only be calculated after 3D image segmentations but are expected to be relevant to the functional mechanics of the IVD, according to the known importance of volumetric mechanical deformations in highly hydrated materials. Therefore, we propose these features as possible novel biomarkers in the study of IDD.

**FIGURE 4:**
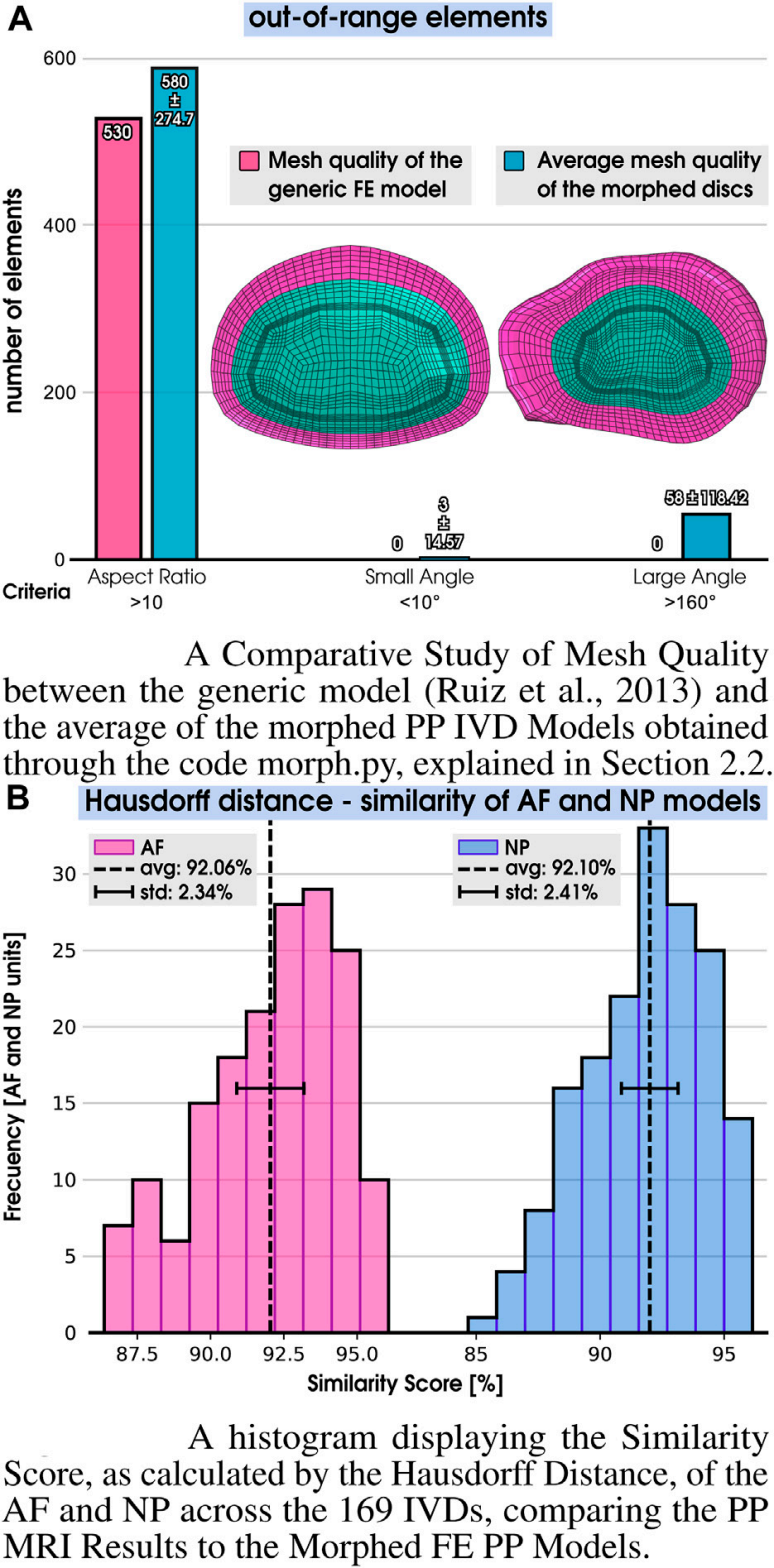
Outcomes from the morphing procedure. **(A)** Mesh quality. **(B)** Similarity score. **(A)** A Comparative Study of Mesh Quality between the generic model (Ruiz et al., 2013) and the average of the morphed PP IVD Models obtained through the code morph.py, explained in Section 2.2. **(B)** A histogram displaying the Similarity Score, as calculated by the Hausdorff Distance, of the AF and NP across the 169 IVDs, comparing the PP MRI Results to the Morphed FE PP Models.

Figure 4 provides an in-depth visual guide to these factors. More specifically, the explored morphological factors of the PP IVD models were: Posterior Height (PH); Middle Height (MH); Anterior Height (AH); Cartilage Endplate Top Height (CTH); Cartilage Endplate Bottom Height (CBH); Sagittal Distance (SD); Coronal Distance (CD); Nucleus Pulposus Perimeter (NPP); Sagittal Area of both the Anterior Zone and Posterior Zone of Annulus Fibrosus (AFAZSA and AFPZSA); Nucleus Pulposus Sagittal Area (NPSA); Cartilage Endplate Top Sagittal Area (CTSA); Cartilage Endplate Bottom Sagittal Area (CBSA); Volumes of Annulus Fibrosus Nucleus Pulposus, and both Cartilage Endplates (AFV, NPV, CTV, and CBV); the Wedge Angle (*α*), defined as the angle formed by the line connecting the upper heights of PH and AH with the line connecting the base of PH and AH. Two different ratios were evaluated as well, signifying the IVD’s intrinsic asymmetry when the sagittal and coronal plane are observed: the Posterior and Anterior Height Ratio (PAHR)—representing the ratio of PH to AH, Sagittal and Coronal Distance Ratio (SCDR)—indicating the disc’s ellipticity:

$$\text{PAHR}=\frac{\text{PH}}{\text{AH}}\;\text{and}\;\text{SCDR}=\frac{\text{SD}}{\text{CD}}$$ (21)

A brief description of the morphological features and their location in Figure 3 is detailed in Table 3.

**TABLE 3:** IVD model morphological features.

Morphological variables				Figure 3
Posterior height	PH	mm	Measured in the sagittal plane	F
Middle height	MH	mm	Measured in the sagittal plane	F
Anterior height	AH	mm	Measured in the sagittal plane	F
Cartilage top height	CTH	mm	Measured in the sagittal plane	B
Cartilage bottom height	CBH	mm	Measured in the sagittal plane	C
Posterior-anterior ratio	PAHR	—	PH/AH Eq. 21	J
Sagittal distance	SD	mm	Measured in the top plane	A
Coronal distance	CD	mm	Measured in the top plane	A
Sagittal-coronal ratio	SCDR	—	SD/CD Eq. 21	K
Nucleus pulposus perimeter	NPP	mm	Measured around the Nucleus	H
Posterior zone area	AFPZSA	mm ^2^	Measured in the sagittal plane	G
Anterior zone area	AFAZSA	mm ^2^	Measured in the sagittal plane	G
Nucleus pulposus area	NPSA	mm ^2^	Measured in the sagittal plane	H
Cartilage top area	CTSA	mm ^2^	Measured in the sagittal plane	I
Cartilage bottom area	CBSA	mm ^2^	Measured in the sagittal plane	I
Annulus fibrosus volume	AFV	mm ^3^	Tissue volume	G
Nucleus pulposus volume	NPV	mm ^3^	Tissue volume	H
Cartilage top volume	CTV	mm ^3^	Tissue volume	I
Cartilage bottom volume	CBV	mm ^3^	Tissue volume	I
Wedge angle	*α*	°	Angle between PH and AH	F

### 4.3 Target variables out of the FE simulations

To refine the selection of mechanical variables for analysis, our approach extends beyond principal stresses and hydrostatic pressure, elements previously examined Tavana et al. (2024); Fleps et al. (2024), to explore potential non-linear interplays with IVD morphology. Moreover, we have incorporated mechanical variables known to affect indirect mechanotransduction phenomena, such as the different strain-dependent or stress parameters related to fluid and proteoglycan contents possibly related to IDD through IVD nutritional aspects (Roberts et al., 1996; Magnier et al., 2009; Travascio et al., 2009; Shirazi-Adl et al., 2010; Malandrino et al., 2015a; Ruiz et al., 2016; De Geer, 2018).

In this context, we selected 11 key mechanical variables, modeled as the response of the 169 PP IVD models to the imposed mechanical loads as target variables. These variables were derived from averages across the three 27-node volumes (PTZ, CNP, and ATZ) and two 529-node surfaces (TC and BC), as introduced previously. They include the indirect mechanotransduction-related variables: Fluid Volume Ratio (*n*_*w*_); Pore Fluid Effective Velocity (‖ ***v***_*f*_‖); Hydrostatic Pressure (*p*); Principal Stress I (*σ*_I_); Principal Stress III (*σ*_III_); Water Content (*n*_*f*_); Fixed Charge Density (*c*_*f*_); Extrafibrillar Fixed Charge Density (*c*_*f*,exf_); Extrafibrillar Water Content (*n*_*f*,exf_); Swelling/Osmotic Pressure (Δ*π*); Void Ratio (*e*). A description of the variables related to the constitutive model through the equations is detailed in Table 4.

**TABLE 4:** Mechanical variables evaluated in each region of interest (Figures 3D, E).

Mechanical variables		Description	Equation
*n*_*w*_	%	Total fluid volume ratio	Eq. 14
‖ ***v***_*f*_‖	mm/s	Magnitude of velocity at which fluid moves through the porous	Eq. 4
*p*	MPa	The mechanical stress due to the fluid pressure within the IVD	Eq. 5
*σ*_I_	MPa	The maximum principal stress within the tissue	Eq. 2
*σ*_III_	MPa	The minimum principal stress within the tissue	Eq. 2
*n*_*f*_	%	Percentage of water by volume within the tissue	Eq. 13
*c*_*f*_	mEq/mL	Density of fixed charges within the tissue matrix	Eq. 16
*c*_*f*,exf_	mEq/mL	Density of fixed charges outside the collagen fibers	Eq. 9
*n*_*f*,exf_	%	Percentage of water by volume outside the collagen fibers	Eq. 10
Δ*π*	MPa	The tissue exerts pressure as it swells due to water uptake	Eq. 8
*e*	%	The ratio of the volume of voids to the volume of solid material	Eq. 20

FLUVR, Fluid Volume Ratio; FLVEL, Pore Fluid Effective Velocity; HidPre, Hydrostatic Pressure; SMax, Principal Stress I, SMin, Principal Stress III; WCont, Water Content; FCHD, Fixed Charge Density; EFCHD, Extrafibrillar Fixed Charge Density; EWCont, Extrafibrillar Water Content; SwePre, Swelling Pressure; VOIDR, Void Ratio.

### 4.4 Machine learning models

We adopted three machine-learning regression models, i.e., LR, SVR, and XGBoostR, each trained using the disc morphological features to predict the target simulated variables. We partitioned our IVD dataset, consisting of features (*X*) and targets (*y*), 60% for training (*X*_train_, *y*_train_), 20% for validation (*X*_val_, *y*_val_), and the remaining 20% for testing (*X*_test_, *y*_test_). We chose the model and the optimal hyperparameters by maximizing the highest R-squared (*r* ^2^) value while ensuring a low mean squared error (MSE).

The implementation relies on the sklearn library in Python. For each of the five regions of interest (PTZ, CNP, ATZ, TC, and BC), we employed the following steps.

#### 4.4.1 Hyperparameter optimization process

We used K-Fold cross-validation for the hyperparameter optimization for the SVR and XGBoostR models (the LR model has no hyperparameters). The training dataset (*X*_train_, *y*_train_) was split into 20 folds. We fixed the random state value at 42 during the shuffling process to maintain consistency and reproducibility. The ranges of the initial hyparameters for both models are shown in Table 5).

**TABLE 5:** Initial values for hyperparameter optimization of Grid Search, Randomized Search, and Bayesian optimization for SVR and XGBoostR models.

SVR params	Hyperparameter optimization model		
	Grid search	Randomized search	Bayesian optimization
*C*	0.1, 1, 10, 100	Log-scaled, 1e-3 to 1e3	Log-scaled, 1e-3 to 1e2
*ϵ*	0.0001, 0.001, 0.01, 0.1	Linear scale, 0.001 to 0.1	Log-scaled, 1e-3 to 1e1
*kernel*	Li, Po, RBF, Sig	Li, Po, RBF, Sig	Li, Po, RBF, Sig
*γ*	Scale, auto, −3 to 3	Scale, auto, −3 to 3	Scale, auto
*δ*_*Po*_	N/A	[2, 5]	[2, 5]

XGBoostR params	Hyperparameter optimization model		
	Grid search	Randomized search	Bayesian optimization
n_estimators_	100, 500, 1000	Integer range, 50 to 2000	Integer range, 50 to 2000
*η*	0.01, 0.1, 1	Uniform scale, 0.01 to 1	Log-scaled, 0.01 to 1
max_depth_	3, 5, 7	Integer range, 1 to 10	Integer range, 1 to 10
min_child weight_	1, 3, 5	Integer range, 1 to 10	Integer range, 1 to 10
Subsample	0.5, 0.7, 0.9	Uniform scale, 0.1 to 0.7	Log-scaled, 0.3 to 7
Colsample	0.5, 0.7, 0.9	Uniform scale, 0.1 to 0.7	Log-scaled, 0.3 to 7

The hyperparameters for SVR included the penalty parameter *C*, the epsilon (*ϵ*) tolerance for errors, the *kernel* function, and the coefficient gamma (*γ*), a parameter specific to certain kernel functions. The kernel functions used include *linear* (Li), *poly* (Po), *rbf* (RBF), and *sigmoid* (Sig), and the degree (*δ*_*Po*_) of the polynomial function was also considered. The hyperparameters for XGBoostR included the number of gradient-boosted trees (n_estimators_), the learning rate (*η*), the maximum depth of the trees (max_depth_), the minimum child weight (min_child weight_), the subsample ratio (subsample), and the column sample by the tree (colsample).

Our strategies for hyperparameter optimization included.

• **Grid Search:** This strategy conducts an exhaustive search through a predefined set of hyperparameters, creating a “grid” of parameter combinations to try. It then trains a model for each combination and evaluates the model’s performance using cross-validation.
• **Randomized Search:** Unlike Grid Search, Randomized Search does not exhaustively try all parameter settings. Instead, it samples a given number of candidates from a parameter space with a specified distribution. This method is more efficient, especially when dealing with many or continuous parameters. By randomly drawing a subset of parameter combinations, it can explore more unique sets of parameters than Grid Search, potentially leading to better results.
• **Bayesian optimization:** The Tree-structured Parzen Estimator (TPE), which is a Bayesian optimization algorithm, is used through the Optuna Study (a framework for hyperparameter optimization). It constructs a probabilistic model based on past trial results and uses this model to suggest the next set of hyperparameters.

For each regression type (SVR and XGBoostR), we chose the best-performing model from the optimized models obtained through grid search, randomized search, and Bayesian optimization on the validation set (*X*_val_, *y*_val_).

#### 4.4.2 Selecting the best machine-learning model

After identifying the optimal hyperparameters, we merged the training and validation sets to create a combined training-validation set (*X*_train, val_, *y*_train, val_). This was used to train the final SVR and XGBoostR models. Since LR does not require hyperparameter optimization, it was directly trained using the training-validation set.

We evaluated the performance of the final model using the testing set (*X*_test_, *y*_test_). Thus, the model (either LR, SVR, or XGBoostR) with the higher *r* ^2^ score was selected.

#### 4.4.3 Influential morphological factors on the mechanical responses

We employed SHAP values to determine each feature’s contribution towards the prediction. We used the Normalized Mean Absolute SHAP Value (Ning et al., 2022), scaled to a range from 0 to 1, as a reliable metric to rank the morphological factors by impact on each mechanical response.

#### 4.4.4 Morphological impact in terms of mechanical variation magnitude

The SHAP values reflect the influence of each morphological variable on each local mechanical prediction, but they do not measure the magnitude of the triggered variation of these mechanical predictions. Hence, to complement the information provided by the SHAP values, we defined a new metric, the PR% (Eq. 22). The PR% quantifies the capacity of a specific morphological feature to uniquely impact the magnitude of a particular mechanical response (PR) relative to the full range of variation of the same mechanical response over the 169 finite element simulations performed with the personalized models (SR), i.e.,:

$$\text{PR\%}=\frac{\text{PR}}{\text{SR}}\times 100\%$$ (22)

The calculation of PR was done with the best (i.e., with the highest *r* ^2^) trained correlation model (LR; SVR; XGBoostR) by calculating a possible range of mechanical response as a result of the sole variation of the considered morphological descriptor, determined over the entire cohort (*n* = 169). Non-varied morphological features were set to their respective average values for each PR calculation, determined over the entire cohort through the chosen regression model. The PR values are also referred to as the ranges of the regression-predicted variations of magnitude of the mechanical responses (specific to each morphological parameter). The SR values were also referred to as the range of the FE-simulated variations of the magnitude of the mechanical responses over the entire cohort of models.

For each PR%, a low %value indicates that a specific morphology cannot capture *per se* the entire range of variation of the magnitude of the mechanical response. In such a case, the range of mechanical response shall result from the combined variations of multiple morphological features. In contrast, a high PR% value indicates that a leading unique morphological feature can explain *per se* the variation of the magnitude of a specific mechanical response.

## 5 Results

### 5.1 Morphing process

A total of 169 PP IVD FE models were successfully created out of the 169 segmented volumes. Each FE model was devoid of zero-volume elements (ZVe = 0), and all models shared the same components and connectivity as the generic FE mesh model, described in Figure 1A. The mesh quality of the generic model and the average mesh quality of all morphed discs, in terms of element aspect ratio and angular distortions, were compared by employing the ABAQUS 2020 mesh quality check functions (Figure 4A). The average aspect ratio increased by 9.4% in the morphed models, with 580 out-of-range elements and a standard deviation (std) of 274.7, compared to 530 in the generic mesh, out of a total of 19,392 hexahedral elements in each model. Small and large angles escalated by 3 (std of 14.57) and 58 (std of 118.42), respectively, in the morphed models.

None of these increases led to error elements, convergence issues, or negative Jacobians during the simulations. Considering all the out-of-range elements of the three mesh quality categories within the created cohort, it represents only 3.3% of the 19,392 elements.

The morphological measurements of the resulting morphed models are listed in Table 6 and detailed in Section 4.2. Here, we can observe that the average CEP height at the disc’s center for the top (CTH) and bottom (CBH) zones were 0.534 ± 0.148 and 0.493 ± 0.151, respectively. This agrees with that observed by Moon et al. (2013), although the out-of-range minimum and maximum values are also shown among the generated models corresponding to discs in advanced stages of degeneration.

**TABLE 6:** Morphological features (Section 4.2) of the created 169 IVDs morphed models obtained by the morphing process (Section 2.2).

Morphological features		Measured values			
		MIN	MAX	avg ± std	GM
PH	mm	4.000	14.541	8.257 ± 1.548	10.508
MH	mm	5.269	18.254	12.307 ± 2.182	14.330
AH	mm	6.064	24.614	11.838 ± 2.822	13.694
CTH	mm	0.230	1.292	0.534 ± 0.148	0.545
CBH	mm	0.182	1.518	0.493 ± 0.151	0.545
PAHR	—	0.364	1.641	0.721 ± 0.171	0.767
SD	mm	30.395	46.604	37.742 ± 3.372	37.959
CD	mm	41.471	61.847	50.143 ± 4.036	49.463
SCDR	—	0.605	0.930	0.754 ± 0.050	0.767
NPP	mm	63.237	119.308	86.613 ± 11.718	94.734
AFPZSA	mm^2^	0.231	1.115	0.542 ± 0.170	0.525
AFAZSA	mm^2^	0.432	2.026	1.053 ± 0.306	1.316
NPSA	mm^2^	1.012	3.813	2.313 ± 0.513	2.878
CTSA	mm^2^	0.057	0.418	0.145 ± 0.044	0.178
CBSA	mm^2^	0.037	0.276	0.138 ± 0.045	0.193
AFV	mm^3^	4.274	18.097	9.637 ± 2.708	10.146
NPV	mm^3^	2.373	13.158	5.566 ± 1.673	8.134
CTV	mm^3^	0.184	1.399	0.432 ± 0.156	0.624
CBV	mm^3^	0.133	1.367	0.436 ± 0.184	0.667
*α*	°	0.081	23.692	5.981 ± 3.476	4.694

The minimum (MIN), maximum (MAX), and average (avg ± std) values of the entire cohort for each of the morphed models and the values of the generic model (GM) are presented. PH, Posterior Height; MH, Middle Height; AH, Anterior Height; CTH, Cartilage Endplate Top Height; CBH, Cartilage Endplate Bottom Height; PAHR, Posterior and Anterior Height Ratio; SD, Sagittal Distance; CD, Coronal Distance; SCDR, Sagittal and Coronal Distance Ratio; NPP, Nucleus Pulposus Perimeter; AFPZSA, and AFAZSA, Sagittal Area of both the Posterior Zone and Anterior Zone of Annulus Fibrosus; NPSA, Nucleus Pulposus Sagittal Area; CTSA, Cartilage Endplate Top Sagittal Area; CBSA, Cartilage Endplate Bottom Sagittal Area, AFV, NPV, CTV, and CBV, Volumes of Annulus Fibrosus, Nucleus Pulposus, and both Cartilage Endplates and *α*: Wedge Angle.

The similarity score (Figure 4B) between each morphed model and its AF and NP counterpart surfaces in the segmented geometrical models was computed using the Hausdorff distance (as detailed in Section 2.2), as seen in step 7 of Figure 1C). On average, the AF and NP yielded a similarity score of 92.06% and 92.10%, respectively. Moreover, the morphing algorithm consistently achieved a similarity score of at least 85%.

No model was discarded for further morphing or during the simulations since all of them met the quality criteria: ZVe = 0 & S% ≥ 85%.

### 5.2 Model validation

**FIGURE 5:**
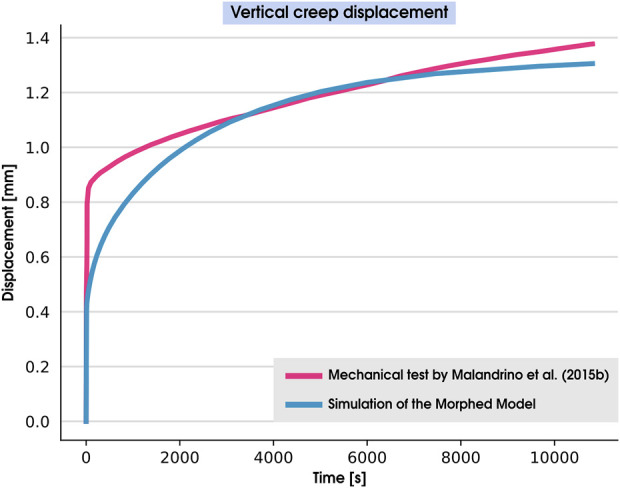
Comparisons between the vertical creep displacement measured during a mechanical test (Malandrino et al., 2015b) and simulated with a corresponding morphed model by using the current PP modeling pipeline.

Figure 5 presents the time history of the vertical displacements of the cranial BEP during the 3-h creep experiment reported in Malandrino et al. (2015b) alongside the simulations of the PP model performed in this work. The simulated creep response was similar to the experimentally measured one (Malandrino et al., 2015b), with a relative error at the end of the simulation of 5.20% between our simulation and the *in vitro* measurements.

### 5.3 Mechanical simulations

#### 5.3.1 Evaluation process and initial values after swelling

The mechanical response was evaluated during the day activity part of the simulated loading history. Over three simulated days (detailed in Section 2.4), the mechanical response for day activity reached equilibrium on the second day. Hence, this specific step was selected for further analysis.

The average values for the initial fixed charge densities and water contents in the AF and NP zones after the swelling step for each of the 169 morphed IVDs were calculated as *c*_*f*,0_ = 0.204 ± 0.003 and *n*_*f*_ = 75.056 ± 0.302% for AF, and *c*_*f*,0_ = 0.309 ± 0.003 and *n*_*f*_ = 79.692 ± 0.162% for NP. These evaluations helped to ensure that the simulations met the estimates detailed in Table 1.

There were no problems with the convergence or the quality of the elements during the simulation for any model, and the Similarity Score (S%) (Eq. 1) for the three models before and after the Swelling are reported in Table 7.

**TABLE 7:** Similarity scores (S%) pre- and post-swelling for IVD models.

ID	Pre-swelling		Post-swelling	
	S% (AF)	S% (NP)	S% (AF)	S% (NP)
MY0092	91.41	91.05	92.80	92.97
MY0002	93.78	91.72	92.53	89.60
MY0065	90.44	92.00	88.90	89.71

#### 5.3.2 Data mining of the mechanical simulations

##### 5.3.2.1 Magnitude of the mechanical responses

Table 8 presents the extreme values of the mechanical responses calculated across the cohort of 169 morphed IVD models (detailed in Table 4) on the three volumes (PTZ, CNP, ATZ) and surfaces (CEP: CT and CB), including the FE-simulated range of variation (SR) and the simulation variation percentage (SV%). The SV% quantifies the relative change in the mechanical response magnitudes, benchmarking the maximum value against the absolute minimum observed in the cohort. This metric is given by the formula:

$$\text{SV}\%=\frac{\left|\text{MAX}-\text{MIN}\right|}{\min\left(\left|\text{MAX}\right|,\left|\text{MIN}\right|\right)}\times 100\%$$ (23)

**TABLE 8:** Summary of Mechanical Variables from the Cohort of 169 Morphed IVDs: Detailing Minimum (MIN), Maximum (MAX) Values, the Simulation Range of Variation (SR), and the Simulation Variation Percentage (SV%) for mechanical response.

Posterior transition zone (PTZ)	*n*_*w*_ %	‖*v*_*f*_‖ mm/s	*p* MPa	*σ*_*I*_ MPa	*σ*_*III*_ MPa	*n*_*f*_ %	*c*_*f*_ mEq/mL	*c*_*f*,*exf*_ mEq/mL	*n*_*f*,*exf*_ %	Δ*π* MPa	*e* %
MIN	7.71e-01	1.63e-05	2.24e-01	−3.28e-01	−3.22e-01	7.66e-01	3.10e-01	3.28e-01	7.19e-01	−2.84e-01	3.36e+00
MAX	7.96e-01	1.58e-04	4.47e-01	−7.01e-02	−1.88e-01	7.97e-01	3.71e-01	3.95e-01	7.53e-01	−2.04e-01	3.89e+00
SR	2.48e-02	1.42e-04	2.22e-01	2.58e-01	1.33e-01	3.04e-02	6.06e-02	6.69e-02	3.41e-02	8.04e-02	5.30e-01
SV%	3.22%	868.58%	99.25%	368.20%	70.73%	3.97%	19.54%	20.42%	4.74%	39.51%	15.76%

Center of nucleus pulposus (CNP)	*n*_*w*_ %	‖*v*_*f*_‖ mm/s	*p* MPa	*σ*_*I*_ MPa	*σ*_*III*_ MPa	*n*_*f*_ %	*c*_*f*_ mEq/mL	*c*_*f*,*exf*_ mEq/mL	*n*_*f*,*exf*_ %	Δ*π* MPa	*e* %
MIN	8.03e-01	4.65e-06	3.40e-01	−3.18e-01	−2.44e-01	7.61e-01	3.41e-01	3.62e-01	7.14e-01	−2.99e-01	4.09e+00
MAX	8.08e-01	2.94e-05	4.71e-01	−2.37e-01	−1.78e-01	7.81e-01	3.81e-01	4.07e-01	7.36e-01	−2.44e-01	4.21e+00
SR	4.68e-03	2.47e-05	1.31e-01	8.09e-02	6.60e-02	1.98e-02	4.04e-02	4.48e-02	2.20e-02	5.45e-02	1.24e-01
SV%	0.58%	531.10%	38.46%	34.15%	37.02%	2.60%	11.84%	12.38%	3.09%	22.33%	3.04%

Anterior transition zone (ATZ)	*n*_*w*_ %	‖*v*_*f*_‖ mm/s	*p* MPa	*σ*_*I*_ MPa	*σ*_*III*_ MPa	*n*_*f*_ %	*c*_*f*_ mEq/mL	*c*_*f*,*exf*_ mEq/mL	*n*_*f*,*exf*_ %	Δ*π* MPa	*e* %
MIN	7.76e-01	1.20e-05	2.21e-01	−3.22e-01	−2.77e-01	7.68e-01	3.17e-01	3.36e-01	7.21e-01	−2.78e-01	3.46e+00
MAX	7.98e-01	9.85e-05	4.10e-01	−8.11e-02	−1.79e-01	7.93e-01	3.67e-01	3.91e-01	7.49e-01	−2.12e-01	3.95e+00
SR	2.21e-02	8.65e-05	1.89e-01	2.41e-01	9.85e-02	2.48e-02	4.95e-02	5.47e-02	2.77e-02	6.66e-02	4.89e-01
SV%	2.85%	719.72%	85.50%	296.94%	55.11%	3.23%	15.60%	16.29%	3.84%	31.48%	14.11%

Cartilage endplate top (CT)	*n*_*w*_ %	‖*v*_*f*_‖ mm/s	*p* MPa	*σ*_*I*_ MPa	*σ*_*III*_ MPa	*n*_*f*_ %	*c*_*f*_ mEq/mL	*c*_*f*,*exf*_ mEq/mL	*n*_*f*,*exf*_ %	Δ*π* MPa	*e* %
MIN	5.91e-01	5.60e-03	3.12e-01	−3.01e-01	−6.23e-01	6.14e-01	1.95e-01	2.59e-01	4.51e-01	−1.89e-01	1.58e+00
MAX	6.04e-01	2.18e-02	4.13e-01	−2.35e-01	−4.57e-01	6.37e-01	2.14e-01	2.92e-01	4.78e-01	−1.57e-01	1.66e+00
SR	1.25e-02	1.62e-02	1.01e-01	6.64e-02	1.66e-01	2.21e-02	1.93e-02	3.23e-02	2.71e-02	3.15e-02	8.11e-02
SV%	2.12%	289.31%	32.34%	28.25%	36.33%	3.60%	9.90%	12.46%	6.01%	20.05%	5.15%

Cartilage endplate bottom (BT)	*n*_*w*_ %	‖*v*_*f*_‖ mm/s	*p* MPa	*σ*_*I*_ MPa	*σ*_*III*_ MPa	*n*_*f*_ %	*c*_*f*_ mEq/mL	*c*_*f*,*exf*_ mEq/mL	*n*_*f*,*exf*_ %	Δ*π* MPa	*e* %
MIN	5.87e-01	4.54e-03	3.19e-01	−3.19e-01	−7.18e-01	6.07e-01	1.97e-01	2.62e-01	4.42e-01	−2.01e-01	1.55e+00
MAX	6.06e-01	1.81e-02	4.58e-01	−9.80e-02	−4.74e-01	6.34e-01	2.21e-01	3.03e-01	4.75e-01	−1.60e-01	1.67e+00
SR	1.90e-02	1.36e-02	1.39e-01	2.21e-01	2.44e-01	2.74e-02	2.43e-02	4.10e-02	3.32e-02	4.12e-02	1.23e-01
SV%	3.24%	298.47%	43.49%	225.60%	51.50%	4.51%	12.36%	15.62%	7.52%	25.75%	7.97%

The SV% values are highlighted to indicate their ranges: 
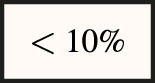
 
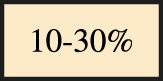
 
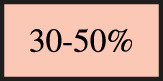
 
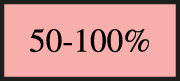
 
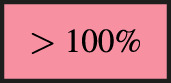
.

The variables that had percentage variations greater than 100% between maximum and minimum values of the entire cohort were the fluid velocity (‖ ***v***_*f*_‖) for all the evaluated zones and the maximum stress (*σ*_*I*_) in the posterior, anterior, and CEP bottom zones (PTZ, ATZ, and BT). Changes of 50%–100% were reflected by the hydrostatic pressure (*p*) in the PTZ and ATZ and the minimum stress (*σ*_*III*_) in the PTZ, ATZ, and BT. Changes between 30% and 50% were calculated for the osmotic pressures (Δ*π*) of the PTZ and ATZ and for the hydrostatic pressures and principal stresses of the CNP and of the CEPs (CT and BT). Values between 10% and 30% were calculated for the fixed charge densities (*c*_*f*_ and *c*_*f*,*exf*_) and void ratio (*e*) of all zones of interest. The most insignificant changes ( < 10%) were obtained for the fluid content-related variables (*n*_*w*_, *n*_*f*_ and *n*_*f*,*exf*_).

In general, the magnitudes of the mechanical responses most affected by the morphological changes were fluid velocity, hydrostatic pressure, and principal stresses. The most significant changes were in the posterior and anterior zones (PTZ and ATZ). In contrast, the magnitude variation in the CNP was smaller, especially for the principal stresses. The osmotic pressure and void ratio magnitude variations were also lower in the CNP compared to the PTZ and ATZ, although similar in percentage variation to the ones in the CEPs (CT and BT).

##### 5.3.2.2 Interpreting machine learning models

**FIGURE 6:**
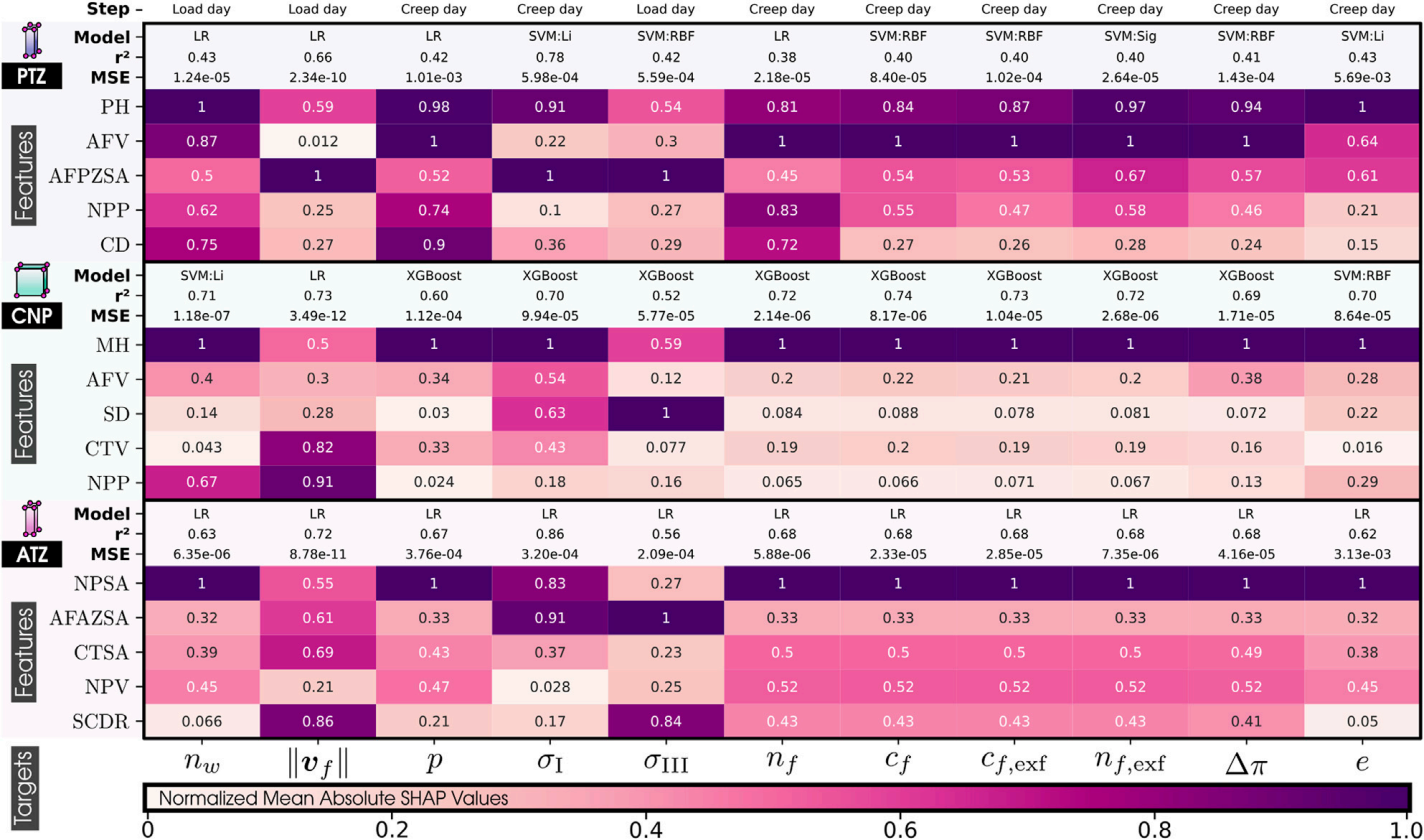
Normalized Mean Absolute SHAP Values on the morphological features (top 5) by their impact on each mechanical response (targets) of our 169 IVD PP models. 20 features are PH, Posterior Height; MH, Middle Height; AH, Anterior Height; CTH, Cartilage Endplate Top Height; CBH, Cartilage Endplate Bottom Height; PAHR, Posterior and Anterior Height Ratio; SD, Sagittal Distance; CD, Coronal Distance; SCDR, Sagittal and Coronal Distance Ratio; NPP, Nucleus Pulposus Perimeter; AFPZSA, and AFAZSA, Sagittal Area of both the Posterior Zone and Anterior Zone of Annulus Fibrosus; NPSA, Nucleus Pulposus Sagittal Area; CTSA, Cartilage Endplate Top Sagittal Area; CBSA, Cartilage Endplate Bottom Sagittal Area; AFV, NPV, CTV, and CBV: Volumes of Annulus Fibrosus, Nucleus Pulposus, and both Cartilage Endplates and *α*: Wedge Angle. 11 targets are *n*_*w*_: Fluid Volume Ratio, ‖ ***v***_*f*_‖: Maginitude of Pore Fluid Effective Velocity, *p*: Hydrostatic fluid pressure, *σ*_I_: Principal Stress I, *σ*_III_: Principal Stress III, *n*_*f*_: Water Content, *c*_*f*_: Fixed Charge Density, *c*_*f*,exf_: Extrafibrillar Fixed Charge Density, *n*_*f*,exf_: Extrafibrillar Water Content, Δ*π*: Swelling/Osmotic Pressure, *e*: Void Ratio. Regression models used, LR, Linear Regression; SVR, Support Vector Machine [*kernel* functions: *linear* (Li), *poly* (Po), *rbf* (RBF), and *sigmoid* (Sig)], and XGBoostR: ExtremeGradient Boosting. PTZ, Posterior Transition Zone; CNP, Center of the Nucleus Pulposus, and ATZ, Anterior Transition Zone.

**FIGURE 7:**
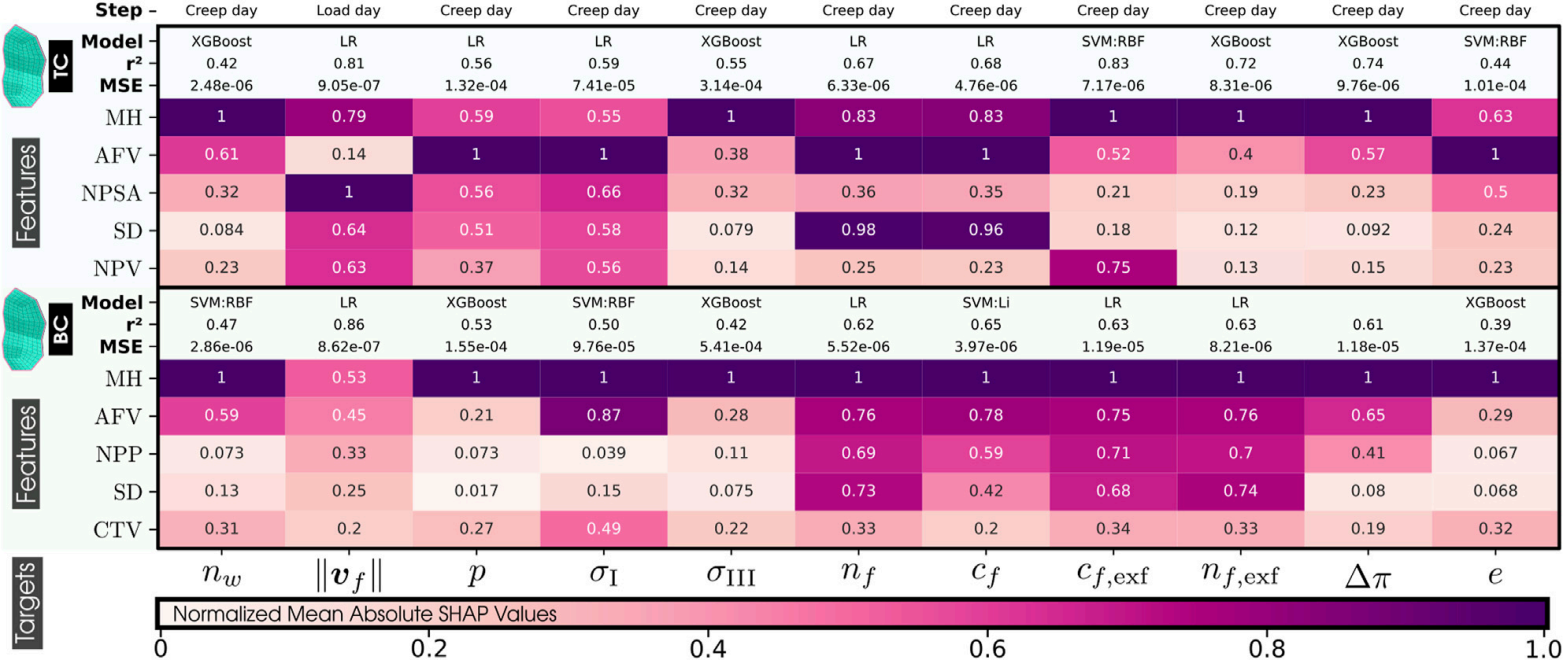
Normalized Mean Absolute SHAP Values on the morphological features (top 5) by their impact on each mechanical response (targets) of our 169 IVD PP models. 20 features are PH, Posterior Height; MH, Middle Height; AH, Anterior Height; CTH, Cartilage Endplate Top Height; CBH, Cartilage Endplate Bottom Height; PAHR, Posterior and Anterior Height Ratio; SD, Sagittal Distance; CD, Coronal Distance; SCDR, Sagittal and Coronal Distance Ratio; NPP, Nucleus Pulposus Perimeter; AFPZSA, and AFAZSA, Sagittal Area of both the Posterior Zone and Anterior Zone of Annulus Fibrosus; NPSA, Nucleus Pulposus Sagittal Area; CTSA, Cartilage Endplate Top Sagittal Area; CBSA, Cartilage Endplate Bottom Sagittal Area; AFV, NPV, CTV, and CBV: Volumes of Annulus Fibrosus, Nucleus Pulposus, and both Cartilage Endplates and *α*: Wedge Angle. 11 targets are *n*_*w*_: Fluid Volume Ratio, ‖ ***v***_*f*_‖: Maginitude of Pore Fluid Effective Velocity, *p*: Hydrostatic fluid pressure, *σ*_I_: Principal Stress I, *σ*_III_: Principal Stress III, *n*_*f*_: Water Content, *c*_*f*_: Fixed Charge Density, *c*_*f*,exf_: Extrafibrillar Fixed Charge Density, *n*_*f*,exf_: Extrafibrillar Water Content, Δ*π*: Swelling/Osmotic Pressure, *e*: Void Ratio. Regression models used, LR, Linear Regression; SVR, Support Vector Machine [*kernel* functions: *linear* (Li), *poly* (Po), *rbf* (RBF), and *sigmoid* (Sig)], and XGBoostR: ExtremeGradient Boosting. CT, Cartilage Endplate Top and CB, Cartilage Endplate Bottom.

Figure 6 shows the contribution of each morphological variable (rows) to the prediction of each mechanical response (columns) for the regression models in the three volumes (PTZ, CNP, and ATZ), while Figure 7 explains the interaction for the two CEP surfaces (TC and BT). The absolute normalized SHAP values reported there quantify the average contribution of a feature to the model predictions across the entire dataset, with a value of 1 representing a maximal contribution. Only the top 5 morphological features are reported in this text so that each mechanical variable does not unnecessarily saturate the figure with less influential features. All morphological features of each volume, PTZ, CNP, and ATZ, and surfaces, TC and BC, of interest are listed in (Supplementary Figures S1–S5, respectively). Specific variables, such as ‖ ***v***_*f*_‖, were assessed after the day’s loading, just before the creep step (see Figure 2), because they showed significant changes with deformations within a brief period.

##### 5.3.2.3 Influential morphological factors on zones of interest

The analysis of Figures 6, 7 indicates that MH is a pivotal morphological feature influencing the trained model of the mechanical behavior across the nucleus and cartilaginous regions (CNP, TC, and BC). Overall, this result highlights a strong correlation between the axial dimension of the disc and the regional biomechanical responses calculated across the organ. Notably, within the nucleus, MH’s influence was so significant that it diminished the relative importance of other morphological factors.

In the posterior zone (Figure 6), the local height, PH, and the posterior sagittal area, AFPZSA, emerged as the most influential features, pointing out the importance of local disc morphology in controlling the mechanical response of the respective region. The overall volume of the AF, AFV, is also a determinant factor in this zone.

In contrast, the ATZ (Figure 6) is predominantly influenced by the nearest sagittal areas, including those of the anterior and nucleus regions (AFPZSA and NPSA). This suggests that the biomechanics of the ATZ is intricately connected to the IVD’s anterior spatial arrangement, i.e., local morphological attributes. Interestingly, the ATZ’s mechanics are less dependent on its own height compared to other regions like the CNP and PTZ.

Even though the cartilaginous regions (TC and BC) analysis reveals a nuanced relationship where the central disc height (Figure 7)—a parameter composed of the heights of both CEPs and the nucleus—exerts a dominant influence, their own local morphology does not affect them. Instead, the mechanical dynamics are shaped by an intricate mix of morphologies beyond the immediate locality, including the sagittal area and perimeter of the nucleus (NPSA and NPP). Moreover, these zones also show sensitivity to the volume of the annulus (AFV), mirroring patterns observed in the PTZ and ATZ.

##### 5.3.2.4 Influential morphological factors on the mechanical responses

Critical variables for nutrient transport (Figure 6), such as those that measure water quantities (Malandrino et al., 2011; Ruiz et al., 2016), charge densities, and pressures, are highly affected by local morphological factors, such as the MH in the CNP and the sagittal area of the nucleus in the ATZ. Both zones, thought to be critical in the early disc degeneration stages, depend on NP-related morphology. This pattern is similarly observed in the cartilaginous regions, where the disc’s central height impacts indirect mechanotransduction variables. However, in the posterior region, they are predominantly affected by the PH, its associated sagittal area (AFPZSA), and the Annulus Fibrosus’s volume (AFV).

Conversely, when it comes to stress-related mechanical variables such as the principal stresses, *σ*_I_ and *σ*_III_, a divergence is observed in the influencing morphological factors. Within the nucleus, the maximum stress correlates mainly with the vertical dimension, MH, whereas the minimum stress is associated with the sagittal distance, SD. In the ATZ, maximum stress linkage is seen with the NP’s and AF’s sagittal areas (NPSA and AFAZSA). In contrast, the minimum stress is influenced solely by the sagittal area of the annulus (AFAZSA). For the posterior region, PTZ, something similar to the previous one is observed; the maximum stress is more about vertical morphologies, and the minimum stress is related to AF and SCDR.

Pore fluid velocity exhibits zone-specific dependencies. In the PTZ, it is primarily governed by its adjacent sagittal area, the AFPZSA. Within the nucleus, the perimeter of the nucleus and cartilage volume take precedence. For the ATZ, this variable is more sensitive to the sagittal-coronal distances ratio (SCDR), underscoring responsiveness to the disc’s general shape or ellipticity. This attribute parallels the sensitivity of the minimum principal stress, *σ*_III_.

Focusing on the CEP surfaces (Figure 7), both principal stresses are significantly conditioned by the MH. Notably, the maximum stress is additionally modulated by the volume of the Annulus Fibrosus, suggesting that both the height and the volumetric attributes of the IVD components collectively influence the mechanical stresses exerted within these regions.

##### 5.3.2.5 Morphological impact in terms of mechanical variation magnitude

**FIGURE 8:**
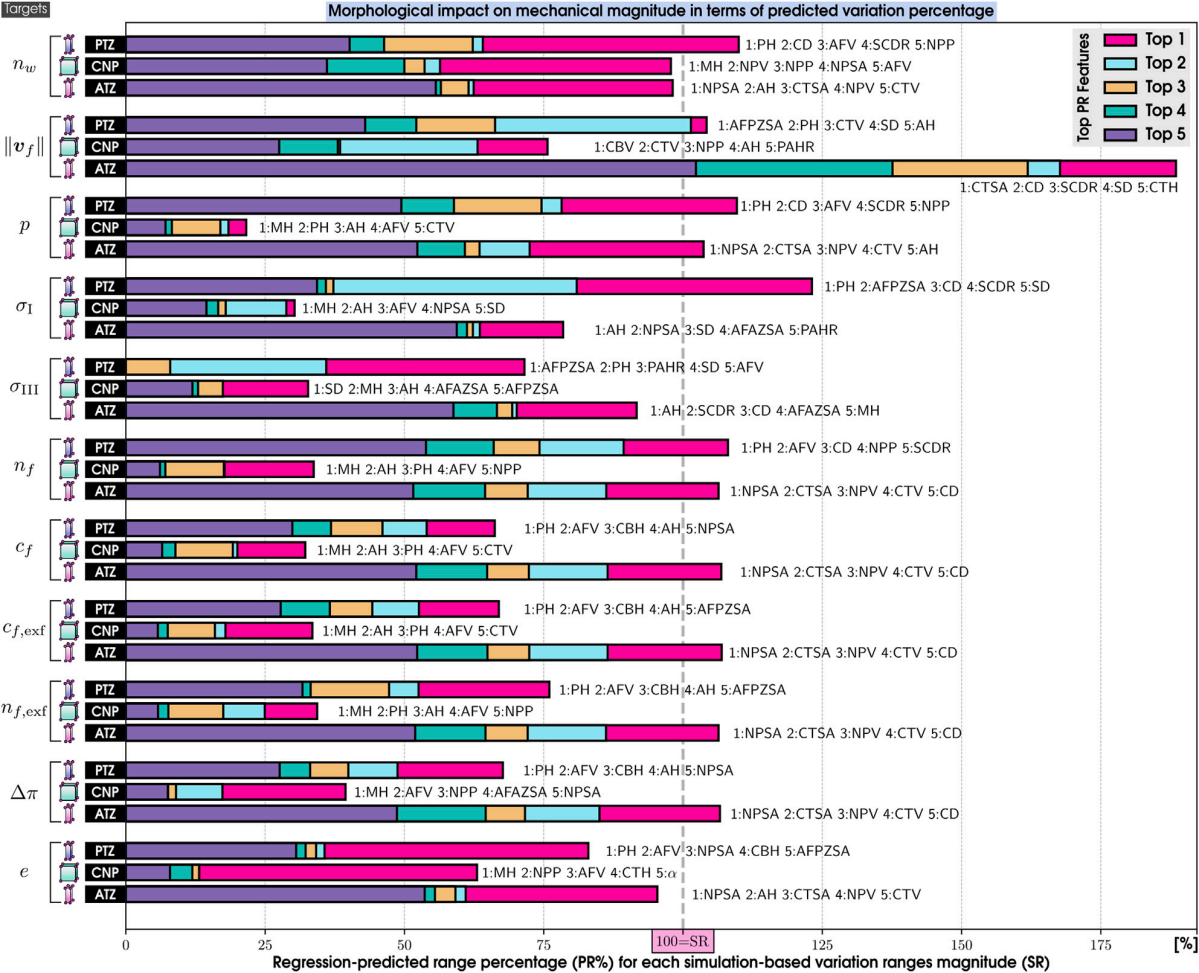
Overlapping bar chart, with the top 5 Morphological impacts (top 1 at the base and top 5 at the peak) on mechanical magnitudes (targets) in terms of predicted variation percentage (PR%) of our 169 IVD PP models. PR%, Regression-predicted range percentage; SR, Simulation-based variation ranges magnitude. The 20 features are PH, Posterior Height; MH, Middle Height; AH, Anterior Height; CTH, Cartilage Endplate Top Height; CBH, Cartilage Endplate Bottom Height; PAHR, Posterior and Anterior Height Ratio; SD, Sagittal Distance; CD, Coronal Distance; SCDR, Sagittal and Coronal Distance Ratio; NPP, Nucleus Pulposus Perimeter; AFPZSA, and AFAZSA, Sagittal Area of both the Posterior Zone and Anterior Zone of Annulus Fibrosus; NPSA, Nucleus Pulposus Sagittal Area; CTSA, Cartilage Endplate Top Sagittal Area; CBSA, Cartilage Endplate Bottom Sagittal Area; AFV, NPV, CTV, and CBV: Volumes of Annulus Fibrosus, Nucleus Pulposus, and both Cartilage Endplates and *α*: Wedge Angle. 11 targets are *n*_*w*_: Fluid Volume Ratio, ‖ ***v***_*f*_‖: Maginitude of Pore Fluid Effective Velocity, *p*: Hydrostatic fluid pressure, *σ*_I_: Principal Stress I, *σ*_III_: Principal Stress III, *n*_*f*_: Water Content, *c*_*f*_: Fixed Charge Density, *c*_*f*,exf_: Extrafibrillar Fixed Charge Density, *n*_*f*,exf_: Extrafibrillar Water Content, Δ*π*: Swelling/Osmotic Pressure, *e*: Void Ratio. PTZ, Posterior Transition Zone; CNP, Center of the Nucleus Pulposus, and ATZ, Anterior Transition Zone.

**FIGURE 9:**
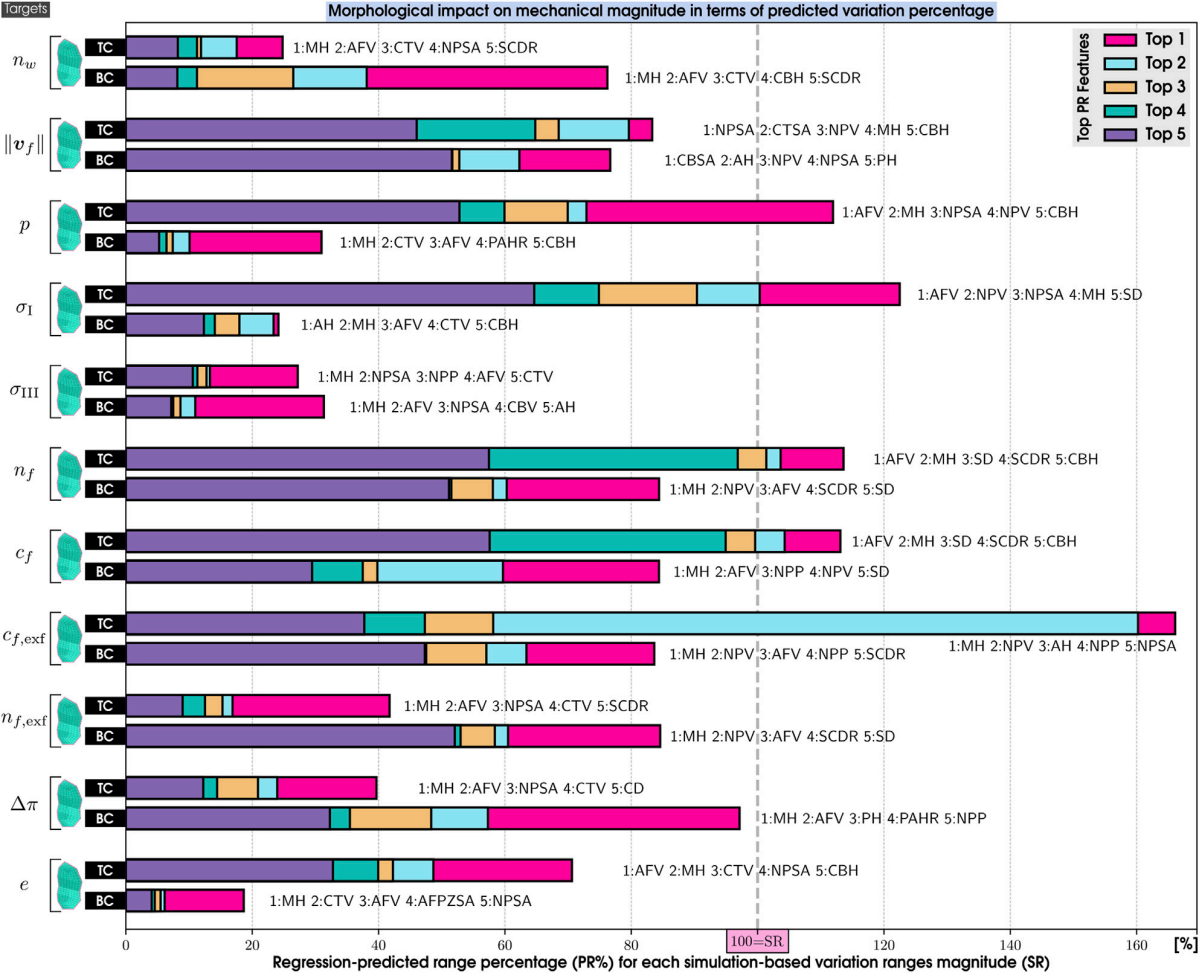
Overlapping bar chart, with the top 5 Morphological impacts (top 1 at the base and top 5 at the peak) on mechanical magnitudes (targets) in terms of predicted variation percentage (PR%) of our 169 IVD PP models. PR%, Regression-predicted range percentage; SR, Simulation-based variation ranges magnitude. The 20 features are PH, Posterior Height; MH, Middle Height; AH, Anterior Height; CTH, Cartilage Endplate Top Height; CBH, Cartilage Endplate Bottom Height; PAHR, Posterior and Anterior Height Ratio; SD, Sagittal Distance; CD, Coronal Distance; SCDR, Sagittal and Coronal Distance Ratio; NPP, Nucleus Pulposus Perimeter; AFPZSA, and AFAZSA, Sagittal Area of both the Posterior Zone and Anterior Zone of Annulus Fibrosus; NPSA, Nucleus Pulposus Sagittal Area; CTSA, Cartilage Endplate Top Sagittal Area; CBSA, Cartilage Endplate Bottom Sagittal Area; AFV, NPV, CTV, and CBV: Volumes of Annulus Fibrosus, Nucleus Pulposus, and both Cartilage Endplates and *α*: Wedge Angle. 11 targets are *n*_*w*_: Fluid Volume Ratio, ‖ ***v***_*f*_‖: Maginitude of Pore Fluid Effective Velocity, *p*: Hydrostatic fluid pressure, *σ*_I_: Principal Stress I, *σ*_III_: Principal Stress III, *n*_*f*_: Water Content, *c*_*f*_: Fixed Charge Density, *c*_*f*,exf_: Extrafibrillar Fixed Charge Density, *n*_*f*,exf_: Extrafibrillar Water Content, Δ*π*: Swelling/Osmotic Pressure, *e*: Void Ratio. CT, Cartilage Endplate Top and CB, Cartilage Endplate Bottom.

The top 5 PR% values (Eq. 22) were calculated for each mechanical variable by using the results of the morphing (Table 6) for the values of the morphological features in the calculation of the PR values. They are represented in Figures 8, 9 for the targeted volumes (PTZ, CNP, ATZ) and surfaces (CEP: CT and CB), respectively. The 100% represents the SR value of each mechanical response (detailed in Table 8).

The magnitudes with the highest simulation variation percentage in their magnitude (SV%), the fluid velocity (‖ ***v***_*f*_‖), was particularly sensitive to CEP morphologies (represented by the volumes CTV and CBV, and by the sagittal area CTSA), especially in the CNP and ATZ areas (Figure 8). The variability of another mechanical response with a high SV% is the maximum stress magnitude, which was mostly controlled by height-related measurements (PH, MH, and AH), while minimum stress (SV% > 50%) levels were influenced by AF dimensions and disc ellipticity (AFPZSA, SD, CD, SCDR). The rest of the mechanical magnitudes changed when the corresponding local morphologies varied, i.e., the CNP by the mid-height (MH) and the PTZ by the posterior height (PH). Remarkably, though, the magnitudes of the mechanical variables in the ATZ were first most affected by the sagittal area of the NP (NPSA) and then by its corresponding local IVD morphology, the anterior disc height (AH). The magnitudes of the mechanical variables in the CEP (Figure 9) were predominantly affected by AF and NP morphologies (mid-height, MH; AF volume, AFV; sagittal area and volume of the NP, NPV, and NPSA) and not by their own morphologies.

In the nucleus center (CNP), the hydrostatic pressure (*p*), the stresses (*σ*_I_ and *σ*_III_), the water contents (*n*_*f*_ and *n*_*f*,exf_), the fixed charge densities (*c*_*f*_ and *c*_*f*,exf_), and the osmotic pressure (Δ*π*) magnitude variation had low PR%, below of 50%. Surprisingly, the principal stress magnitudes (*σ*_I_ and *σ*_III_) in the CNP could not be affected by more than 30% of the SR by individual morphological features. This is also consistent with the low SV%, so the magnitude does not vary as much as in the PTZ and ATZ.

**FIGURE 10:**
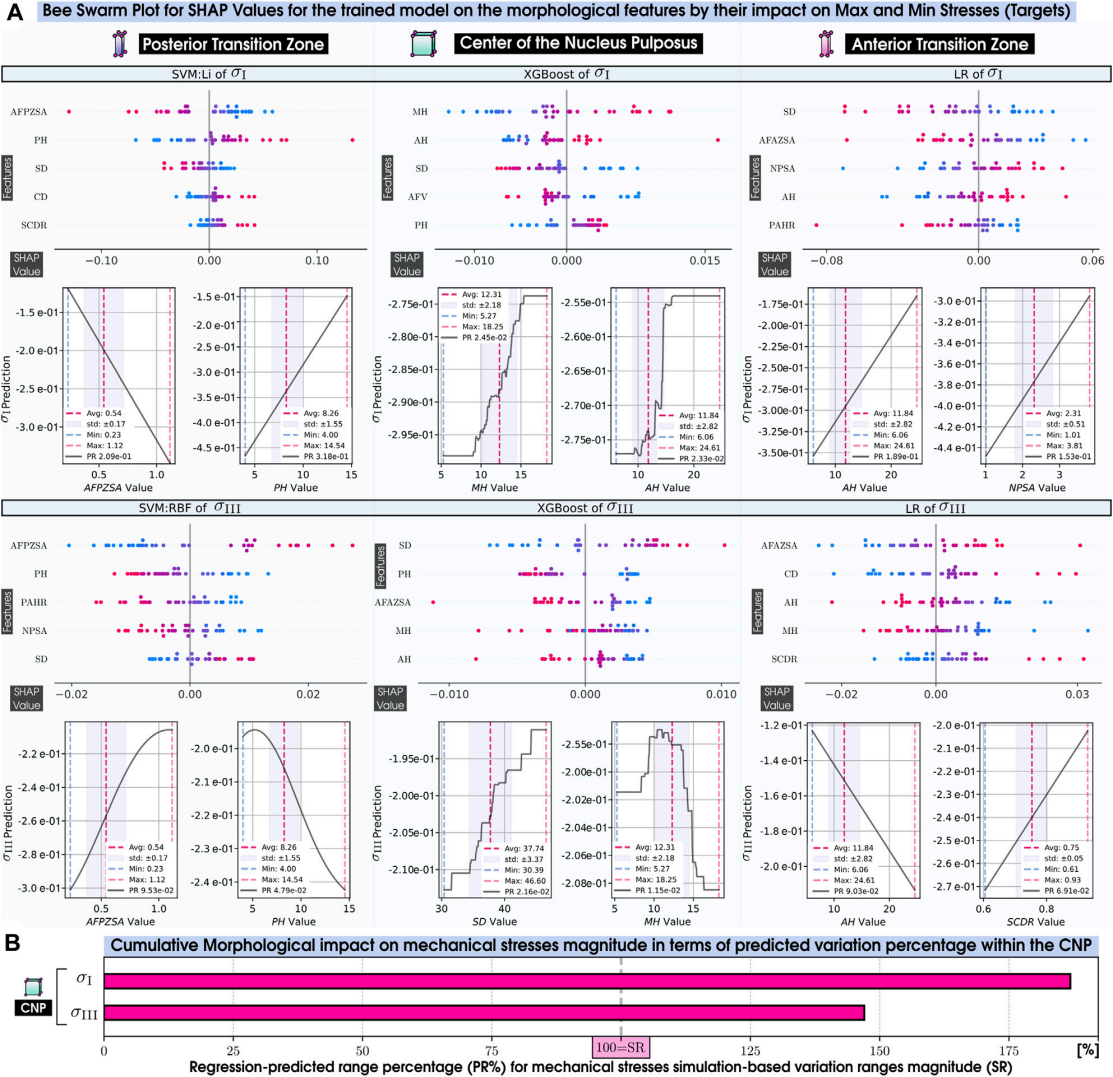
Morphological impact on mechanical stresses. **(A)** Bee Swarm Plot for SHAP Values for the trained model on the morphological features by their impact on Max and Min Stresses (Targets). **(B)** Cumulative morphological impact of the 20 features on the mechanical stresses within the CNP. PR, Regression-predicted variation ranges magnitude; SR, Simulation-based variation ranges magnitude. PTZ, Posterior Transition Zone; CNP, Center of the Nucleus Pulposus, and ATZ, Anterior Transition Zone. The description of the morphological features can be found in Table 3.

The SHAP values, detailed in the Bee Swarm Plot (Figure 10A), indicated how strongly a morphological feature variation increased or decreased the magnitude of the stress in each region of interest. The stress graphs show that the regression-predicted variations in stress magnitude in the CNP (PR = e−02) were one order of magnitude lower than those in the PTZ and ATZ (PR = e−01) regions. However, the non-linearity observed in the CNP for both stresses and in the PTZ for the minimum stress shows that the minimum and maximum magnitudes do not necessarily correspond to the minimum and maximum values of the specific evaluated morphology. In the CNP, the cumulative impact of all morphological features on both stress variations (Figure 10B) had to be considered to reach or exceed 100% of the SR.

For further insight into the interplay between morphology and mechanical magnitude across all five zones of interest (PTZ, CNP, ATZ, CT, and CB), please refer to the (Supplementary Figures S6 through S10).

## 6 Discussion

### 6.1 Morphing process, accuracy, and validation

Our morphing process successfully produced 169 PP IVD models, with the mesh quality remaining consistent with the generic FE mesh model, even if minor increases in out-of-range elements occurred. Notably, the increase in aspect ratio criterion and small and large angles did not affect the convergence or the simulations, demonstrating the overall quality of the generated mesh and the ability of the algorithm to restrict the excessive growth of hexahedral edges. Precise deformation control was reflected by the similarity scores for AF and NP, which were 92.06% and 92.10%, respectively (Figure 4B). The BCPD++ algorithm maintained the relative distances and positions between FE mesh nodes, which prevented any single edge of a hexahedral element from growing disproportionately, achieving a patient-personalized FE model of the IVD efficiently, based on segmented medical images, with full respect to important tissue-specific mesh structures (Ruiz et al., 2013). Arguably, this approach does not allow the modeling of structural tissue damage, such as fissures or annular tears. Yet, the effect of these damages can be captured with proper homogenized continuum models and parameters, as proposed by Malandrino et al. (2015b).

As mentioned in Section 4.2, most of the research related to morphology and degeneration focuses on the diversity of disc heights. For instance, mid-height (MH) discs ranging from 5 to 16 mm have been used to determine relationships between IDD and MH (Teichtahl et al., 2015). In the present study, we examined the MH, which ranged from 5 to 18 mm. Other studies have correlated the three heights of the IVD with lumbar disc herniation (Kizilgöz and Ulusoy, 2019), with corresponding values of posterior, middle, and anterior as PH: 2–13 mm, MH: 3–15 mm, and AH: 3–18 mm, respectively. Our cohort includes discs with values of PH: 4–14 mm, MH: 5–18 mm, and AH: 6–24 mm, thus representing a diverse array of shapes that cover an extensive range of morphologies in healthy and degenerated discs. This further validates the method’s capability to preserve vital geometric characteristics.

The average CEP height at the center of the disc for the top (CTH) and bottom (CBH) zones measured 0.534 ± 0.148 mm and 0.493 ± 0.151 mm, respectively. These averages align with the findings by Moon et al. (2013) of 0.54 ± 0.12 mm, showcasing the robustness of our modeling approach. Additionally, the range of values captured includes minimum and maximum measurements corresponding to the variation expected in discs exhibiting advanced degeneration, reflecting the diversity of IVD conditions in the population.

The importance of our pipeline and methodology becomes particularly evident when compared to other techniques, such as the one proposed by Fleps et al. (2024), which also used segmented medical images to achieve personalized IVD model geometries. However, these only captured the outer surface of the AF, neglecting to personalize the Nucleus Pulposus individually. Our findings highlight the critical role of the NP in influencing indirect mechanotransduction responses. Specifically, parameters crucial for nutrient transport, including charge densities, water content, and osmotic pressure (represented by *c*_*f*_, *n*_*f*_, *c*_*f*,exf_, *n*_*f*,exf_, and Δ*π*), as mentioned in Section 4, especially in the posterior and anterior zone (PH and AH) and both cartilaginous regions, top and bottom (TC and BC), cannot be accurately replicated with the same level of personalized precision using such external-surface-focused techniques. Furthermore, our morphing technique has been uniquely tested on a significantly larger sample of IVD morphologies [*n* = 169 vs. *n* = 22 in Fleps et al. (2024)].

One of the primary challenges we faced in this study was the inability to replicate the morphology of the CEP due to the inherent limitation of MRI scans in delineating the external surface of this thin tissue. To overcome this obstacle, we implemented a model customization strategy. Specifically, we adjusted the dimensions of the CEP to fill the volume between the NP’s cranial and caudal surfaces and the external AF. This adaptation involves manipulating the *λ* variable in the third step of our morphing process, as explained in Section 2.2. Since the AF/IVD and the NP external surface are directly provided by image segmentations, a certain level of personalization of the CEP is uniquely achieved for each set of segmented disc tissue surfaces, although direct verification or validation of the FE CEP morphology was not possible. Remarkably, the algorithm allows the parameterization of the CEP thickness, in that case, adjusting the cranial and caudal external surfaces of the NP coherently. Though this feature was not used in the current study, it paves the way to systematically explore the relative importance of the CEP through models and simulations. Such explorations might support simulation-based identifications of advanced early image biomarkers associated with the risk of IDD or targeting of CEP engineering-based new regenerative strategies.

Another limitation of our study pertains to the NP-AF boundary transition zone (TZ), which, despite its relevance introduced in the Methods and noted by Ruiz et al. (2013), is difficult to define precisely in terms of material properties and size. Yet, the delineation of this region enhances the realism of the IVD model by providing a gradual material property transition from the NP to the AF, as opposed to an abrupt shift, as suggested by measurements of gradual shifts in cell phenotypes (Bruehlmann et al., 2002), MRI signal (Kapoor et al., 2023), and fiber structure (Disney et al., 2023). Arguably, this region’s local properties and exact size are difficult to assess. Even so, the TZ is physically interesting, as it represents a singular volume of increased radial compression at the periphery of the inner IVD, where the NP material is pressed against the confining annulus material because of the lateral expansion of the nucleus under external mechanical loads. This mechanism potentially influences the transport of nutrients to the cells in the TZ, as suggested by FE models and simulations (Ruiz et al., 2016; Ruiz et al., 2018). Such regional particular tissue physics coincides with the reported local emergence of relatively early signs of tissue disorganization in IVD (Leung et al., 2006; Smith et al., 2011). Moreover, the used segmentations, refined through image fusion (Castro-Mateos et al., 2014), may not have fully captured the shape of the complex NP-AF boundary, even with an improved resolution of 0.68 mm isotropic voxels. Acknowledging this limitation might be crucial to accurately represent this intricate boundary in future models, though some segmentations did represent inward bulging of the AF in degenerated discs^1^.

Additionally, it is essential to recognize our models’ limitations related to the IVD-vertebrae interface. Arguably, the detailed mechanics of the BEP-CEP system cannot be fully captured with the current meshes, as these do not include full vertebrae.

Finally, the validation of the simulations against the research by Malandrino et al. (2015b), with a relative error of just 5.20%, demonstrated the ability to capture the real morphology with the morphing process, and the osmo-poro-hyper-viscoelastic model (referenced in Section 2.3) and the mechanical and tissue properties (listed in Table 1) in representing the time-dependent behavior of a healthy IVD under compression, as simulated hereby to represent average load cycles of daily life.

### 6.2 Mechanical simulations of daily loads

The mechanical simulation results stabilized by the second day. Examination of fixed charge densities and water content (*c*_*f*,0_ and *n*_*f*_) across the morphed PP models confirmed that second-day equilibrium was achieved regardless of the particularities of the modeled morphologies. This verification of the consistency of model initialization was crucial to comparatively analyze the mechanical behaviors of the different disc models after the simulated swelling when reaching the *c*_*f*,0_ and *n*_*f*,0_ proposed values in Table 1.

The analysis revealed that the post-swelling Similarity Scores of the models with mid heights ranging from 8 to 17 mm remained relatively stable, as detailed in Table 7. This stability suggests that despite its potential to modify the morphed shape, the swelling process does not significantly impact the geometry. This resilience in shape demonstrates the simulations’ effectiveness in capturing the true geometry of the IVD for both the AF and NP throughout the present PP modeling process.

A key finding of this research was the correlation between the mechanical responses within specific local volumes in the IVD (Posterior Transition Zone—PTZ, Center of the Nucleus Pulposus—CNP, and Anterior Transition Zone—ATZ) and the corresponding local morphological characteristics of the disc. Overall, results suggested that local disc shapes preponderantly altered local disc mechanics and shall be considered in personalized modeling. The mechanical variables in the CEP were significantly influenced by the middle height (MH) and the AF and NP volumes (AFV and NPV), except the fluid velocity (‖ ***v***_*f*_‖) that depended more on the CEP sagittal areas (CTSA and CBSA). This demonstrates the impact of the morphology of specific disc regions on biomechanical variables in other regions, probably caused by particular volume distributions, i.e., the mechanical variables in the reduced volume of the CEP are impacted by the deformation of larger volumes from other regions. Likewise, the ATZ, a region reported to be affected (Section 4.1) already in early stages of IDD (Leung et al., 2006; Smith et al., 2011), was more influenced by adjacent morphological features, such as the NP and CEP sagittal areas (NPSA and CTSA) than by its own height.

The local mechanical variables reported to impact indirect mechanotransduction phenomena, possibly involved in early IDD (see Section 4.3), are highly influenced by the corresponding local morphology. Such outcome suggests that indirect mechanotransduction is affected by disc height and local morphological factors in each evaluated zone, as we explain above. Meanwhile, maximum and minimum principal stresses (*σ*_I_ and *σ*_III_) are respectively impacted by different morphological features. On one hand, the MH, PH, and the nucleus sagittal area (NPSA) predominantly affected the maximum principal stress, *σ*_I_. On the other hand, the minimum principal stress, *σ*_III_, was more influenced by ellipticity-related variables (ellipticity, SCDR, and sagittal distance, SD) and the posterior and anterior Annulus Fibrosus sagittal areas (AFPZASA and AFAZSA). These insights suggest that in-depth evaluations of IDD require a nuanced analysis that considers local morphological features. Specifically, the assessment of tensile stresses demands a detailed consideration of local axial morphologies, and the compressive stresses call for an exploration of horizontal dimensions related to the ellipticity of the disc to fully understand the mechanical interplays that might contribute to IDD.

In General, the PR% for the volumes and surfaces (Figures 8, 9) captured less than 100% of the SR, indicating that regression-based ranges were included within the simulation-based-ranges. However, in the ATZ, the velocity was particularly sensitive to the cartilage sagittal area (CTSA) and to the ellipticity of the disc (SD, CD, SCDR), so much that every top 5 features exceeded 100% of the SR. This emphasizes the importance of the cartilage endplate in critical areas in the early stages of IDD, such as the anterior zone of the IVD.

Additionally, our influential morphology results (Figure 6) align with prior experimental findings that emphasize the central role of the mid-height (MH), influencing the center of the nucleus (CNP) behaviors (Teichtahl et al., 2015; Kizilgöz and Ulusoy, 2019). However, our analysis suggests that the influence is not necessarily reflected in the magnitude of the mechanical response. The MH highly influences the behavior of the CNP, but the magnitude (Figure 8) of the hydrostatic pressure, stresses, water contents, charged densities, and the osmotic pressure do not exceed 50% magnitude variation. Nevertheless, the cumulative impact of all morphological features on both stress variations (Figure 10B) exceeded 100%, implying the necessity for a holistic morphological approach to understanding CNP mechanics.

These calculations reveal the challenge of isolating a single morphological factor as a single leading controller of the mechanical response of the disc. First, we point out possible non-linear relationships between morphology and mechanics. For example, while mid-height (MH) may be a predominant factor, its increase does not necessarily correlate linearly with the disc mechanical behavior (Figure 10A). Second, the influence of morphology is not defined by a single factor (as observed in Figures 6, 7); adjacent morphologies can significantly alter the expected mechanical outcomes by their influence and magnitudes (Figures 8, 9), thereby complicating the ability to attribute mechanical behavior to a singular morphological aspect. This complexity accentuates the need for a comprehensive customization approach of the IVD 3D morphology, including the AF and the NP, in FE simulations for achieving more accurate and realistic simulations.

Some of our morphological features, while typically not assessed in radiographic evaluations, influenced significantly the predicted internal biomechanics of the IVD and could serve, thus, as novel and potentially crucial image-based biomarkers in a clinical context. For instance, the sagittal areas of the annulus and nucleus (AFPZSA, AFAZSA, and NPSA) are readily identifiable on MRIs. Other features, such as the volumes of the annulus, nucleus, and CEPs (AFV, NPV, and CTV and CBV), as well as the nucleus pulposus perimeter (NPP), can be quantified through 3D segmentation (refer to Figure 3 for morphological factors). Current progress in medical image analysis and processing let us envision the forthcoming availability of automated tool capable of generating 3D lumbar spine models from 2D images, e.g., as proposed for osteoporosis assessments (Lopez Picazo et al., 2018).

Although the present study uniquely revealed the intricate interactions among intervertebral disc morphology and internal biomechanics, it has some limitations. Our boundary conditions represent a normal subject with diverse physiological physical activity during the day and lying at night, as reflected by *in vivo* measurements of intradiscal pressures (Wilke et al., 1999). Although this should be studied following a person’s typical activities, the current approach serves as a standard loading scheme to evaluate a large number of PP models. Since the objective was to focus only on morphology, material properties specific to each degree of degeneration have not been considered. This limitation shall be addressed in future studies, in particular, seizing the opportunity to incorporate Pfirrmann grade-specific material properties (Malandrino et al., 2015b; Barthelemy et al., 2016; Ruiz et al., 2016). Performing morphing for both the AF and NP was challenging due to its difficulty. However, we have achieved it with acceptable levels of similarity to the real model without damaging the mesh, becoming one of the strengths of our work.

The tensile and the compressive stresses are, respectively, the maximum and the minimum principal stresses, which are the local eigenvalues of the stress tensor. Yet, we disregard the analysis of the orientations of these eigenvectors because regardless of the latter, the stress is, indeed, suffered by the materials. Looking at the local orientations of the eigenvectors would be interesting to analyze for future work.

Although the generated IVD files (.inp) are ABAQUS files, these are in ASCII format and can be converted so that models can be reused with alternative open-source software suites, such as FEBio (Maas et al., 2012), MoFEM (Kaczmarczyk et al., 2020), High-Performance Computing (HPC), among others.

## 7 Conclusion

This work provides the first FE model database to study IDD and thoroughly analyzes the intricate relationships between the morphological characteristics of the IVD and its indirect mechanotransduction behavior, employing sophisticated simulation, machine learning techniques, and the BCPD ++ algorithm. The generation and morphing of 169 IVD models revealed remarkable accuracy and adaptability in aligning the generic FE mesh model with MRI-derived PP models without compromising the mesh’s element integrity or the quality of their complicated and varied morphology.

The validation, which yielded acceptable relative errors, underscores the effectiveness and fidelity of our PP modeling approach to replicate healthy IVD behavior under compression. To the best of our knowledge, our comprehensive dataset of 169 simulations of PP FEM of the IVD is unique, as are our collection of mechanical results and data mining analyses. These offer the most detailed insights into data about how local morphologies intricately influence mechanical responses in specific zones of the IVD, including the ones that might be relevant in early IDD. Thanks to the current modeling pipeline, this cohort could be augmented to achieve even more precise results. These insights shall not only enrich our understanding of IVDs’ structural and functional behavior but shall also have significant implications for future research and simulation-based early prevention, diagnostics, and therapeutic interventions for IDD.

This study led to an open-access repository (Muñoz-Moya et al., 2023) -accessible through our online user interface (https://ivd.spineview.upf.edu/). We hope that our research can contribute to standardizing methods for promoting translational IVD and IDD research through *in silico* methods that can be coupled with medical data. This initiative aims to provide a strong foundation for innovation in IVD research, paving the way for further investigations into the multifaceted nature of IVD mechanics, with potential implications for IDD diagnosis, treatment, and prevention strategies.

## Data Availability

The generated/analyzed datasets of 169 IVD PS FE models can be found for free use at the open repository Zenodo Muñoz-Moya et al., 2023, accessible through our online user interface: https://zenodo.org/records/8325042.

## Footnotes

1 e.g., https://ivd.spineview.upf.edu/?filenamePrefix=MY0004_L1L2, https://ivd.spineview.upf.edu/?filenamePrefix=MY0073_L4L5
