# Supplementary material for: Unveiling interactions between intervertebral disc morphologies and mechanical behavior through personalized finite element modeling
Source: Front Bioeng Biotechnol. 2024 Jun 10;12:1384599. doi: 10.3389/fbioe.2024.1384599 (PMC11194671; doi:10.3389/fbioe.2024.1384599)
Supplement: Supplementary file 1 [file DataSheet1.pdf]

Supplementary Material:
Unveiling Interactions Between Intervertebral Disc Morphologies and Mechanical Behavior through Personalized Finite Element Modeling

1 CONSTITUTIVE MODELING OF THE IVD

The IVD material model considers (1) a solid phase comprising structural macromolecules such as collagen, elastin, and proteoglycans, alongside cells and (2) a fluid phase consisting of water and solutes (Malandrino et al., 2015). The biphasic-swelling (BS) theory, as detailed by Mow et al. (1980, 1989), delineates both the equilibrium and transient mechanics of charged soft tissues in IVDs. This theory presents each tissue as a composite material featuring a charged solid porous phase saturated by interstitial fluid, thereby enabling the simulation of fluid pressurization and movement within the disc.

This study characterizes the behavior of the entire disc through an osmo-poro-hyper-viscoelastic model. This comprehensive model integrates the constitutive tissue of the bony endplate, treated as a linear poroelastic material (Malandrino et al., 2011) through shell elements. Furthermore, the annulus, nucleus, cartilage, and transition zone, represented with second-order hexahedral elements, employ the BS model used to simulate poromechanical interactions within a poro-hyperelastic matrix saturated with intra- and extra-fibrillar fluid (Wilson et al., 2005b), including the Donnan osmotic pressure gradient effects (Urban and Maroudas, 1981), which, according to Darcy's law, governs the flow of interstitial fluids through the tissue's porous matrix (Wilson et al., 2005b). In addition, the model considers viscoelastic collagen fibers present only in the AF Wilson et al. (2006a) as rebar elements.

The total stress tensor $\boldsymbol{\sigma}_{\text{tot}}$ is expressed as the superimposition of the effective stress $\boldsymbol{\sigma}_{\text{eff}}$ (defined in 1.0.1) of the solid skeleton within the pores, a fluid pore pressure component p, and Darcy's law:

$$\boldsymbol{\sigma}_{\text{tot}} = \boldsymbol{\sigma}_{\text{eff}} - p\boldsymbol{I} \tag{S1}$$

$$\boldsymbol{q} = \boldsymbol{\kappa}\nabla p \tag{S2}$$

where $\boldsymbol{I}$ is the identity tensor, $\boldsymbol{q}$ is the fluid mass flow to the spatial gradient of pore pressure ∇p, and $\boldsymbol{\kappa}$ is the hydraulic permeability tensor of the tissue. Also, the fluid flow can be expressed by:

$$\boldsymbol{q} = \boldsymbol{u}_f n_f \tag{S3}$$

where $\boldsymbol{u}_f$ is the pore fluid velocity, and n_f represents the total water fraction, i.e., the porosity of the medium.

Due to the fixed charges, the cation concentration inside the tissue is higher than in the surrounding body fluid (Wilson et al., 2005b). This excess of ion particles within the matrix creates the Donnan osmotic pressure, $\Delta\pi$, which drives the fluid flow, causing the swelling of the tissue (Urban et al., 1979).

Incorporating the osmotic pressure into Equation S1, where Schroeder et al. (2007) adapted this equation to the IVD, the hydrostatic fluid pressure p is defined as:

$$p = u_w + \Delta\pi \tag{S4}$$

u_w is the water chemical potential, linked with the pore pressure degree-of-freedom generated by the interstitial fluid permeation effects through the permeability (introduced in 1.0.5) by applying Darcy's law to describe a relationship between fluid flow and the swelling pressure. Therefore, fluid flow between the different tissues of the model depends on the tissue-specific mappings of permeability. $\Delta\pi$ represents the osmotic pressure gradient generated by the difference between the internal and external salt concentrations (more details in Section 1.0.3).

1.0.1 Solid matrix - Non-fibrillar Part

While it is a foundational assumption that the organic solid matrix and the interstitial fluids are intrinsically incompressible, the overall solid matrix displays compressibility attributable to its porous structure (Mow et al., 1980). Addressing this, Wilson et al. (2005b) have introduced a formulation specifically designed for large deformations. This is particularly relevant as substantial pore collapses can generate incompatibilities between the hypotheses of solid matter incompressibility and porous solid compressibility. Consequently, they propose that the effective bulk modulus should be considered strain-dependent. To model the behavior of the non-fibrillar solid matrix within this framework, Wilson et al. (2005a) adopted the following compressible neo-Hookean model:

$$\boldsymbol{\sigma}_{\text{eff}} = K_m \frac{\ln(J)}{J} \boldsymbol{I}_1 + \frac{G_m}{J}\left(\boldsymbol{B} - J^{\frac{2}{3}} \boldsymbol{I}_1\right) \tag{S5}$$

where J is the determinant of the deformation gradient tensor $\boldsymbol{F}$, and $\boldsymbol{I}_1$ is the first invariant of the left Cauchy–Green strain tensor $\boldsymbol{B} = \boldsymbol{F} \cdot \boldsymbol{F}^{\mathsf{T}}$. The bulk, K_m, and the initial shear modulus, G_m, are defined as:

$$K_m = \frac{E_m}{3(1 - 2\nu_m)} \tag{S6}$$

$$G_m = \frac{E_m}{2(1 + \nu_m)} \tag{S7}$$

where E_m is the Young's Modulus and ν_m the Poisson's ratio.

For a solid fraction of 1, if the fluid is fully expelled, there are no pores, making the entire matrix incompressible. Conversely, as the solid fraction goes to zero, the volume fraction of the pores goes to 1, rendering the solid matrix fully compressible. To include this in the model, Wilson et al. (2005b,a) proposed the following expression for the Poisson's ratio:

$$\nu_m = 0.5 n_s = 0.5 \frac{n_{s,0}}{J} \tag{S8}$$

where $n_{s,0}$ and n_s are the initial (in the unloaded and non-swollen state) and current solid volume fraction.

Note that ν_m goes to 0.5 for a solid fraction of 1 and becomes zeros when the solid fraction goes to zero. This term of Equation ensures that tissue compressibility vanishes as J approaches $n_{s,0}$, i.e., the fluid is fully expelled. By substituting Equation S8 into Equation S6 and S7, and by eliminating E_m we get the following expression for K_m:

$$K_m = \frac{2}{3} G_m \frac{(1 + 0.5 n_{s,0}/J)}{(1 - n_{s,0}/J)} \tag{S9}$$

Because the second law of thermodynamics needs to be fulfilled, the constitutive relation for Km cannot be directly substituted in Equation S5. Therefore, it is first implemented into the energy function and derives the new Cauchy stress from the energy function:

$$\begin{aligned} W_{nf} =& \frac{1}{8} K_m \ln^2(\det(\boldsymbol{C})) + \frac{1}{2} G_m \left(\mathrm{tr}(\boldsymbol{C}) - 3\det(\boldsymbol{C})^{1/3} \right) \\ =& \frac{1}{12} G_m \left(\frac{(1 + 0.5 n_{s,0}/J)}{(1 - n_{s,0}/J)} \right) \ln^2(\det(\boldsymbol{C})) + \frac{1}{2} G_m \left(\mathrm{tr}(\boldsymbol{C}) - 3\det(\boldsymbol{C})^{1/3} \right) \end{aligned} \tag{S10}$$

where $\boldsymbol{C}$ is the Cauchy-Green deformation tensor.

Now, the macroscopic stress-strain response of the solid matrix is determined by G_m, $n_{s,0}$, and the current deformations of the homogenized poroelastic continuum. This response follows the Cauchy stress of the non-fibrillar matrix to describe the material's finite strain behavior, as Wilson et al. (2005b,a, 2006a,b) detailed initially and then adapted by Schroeder et al. (2008) for the IVD:

$$\boldsymbol{\sigma}_{\text{eff}} = -\frac{1}{6} \frac{\ln(J)}{J} G_m \boldsymbol{I}_1 \left[-1 + \frac{3(J + n_{s,0})}{(-J + n_{s,0})} + \frac{3J \ln(J) n_{s,0}}{(-J + n_{s,0})^2} \right] + \frac{G_m}{J} \left(\boldsymbol{B} - J^{\frac{2}{3}} \boldsymbol{I}_1 \right) \tag{S11}$$

1.0.2 Annulus Fibrosus Collagen Fibers - Fibrillar Part

In the AF, collagen fibers exhibit a unidirectional viscoelastic mechanical response. This behavior is modeled by incorporating finite strains in a Zener viscoelastic model with two non-linear springs. Assuming that the fibrils only resist tension, the Cauchy fibril stress tensor in a unit area for viscoelastic fibrils (Wilson et al., 2006a,b) can be expressed as:

$$\boldsymbol{\sigma}_{\text{f}} = \frac{\psi}{J} P_f \vec{e}_f \vec{e}_f \tag{S12}$$

where ψ is the elongation of the fibril, P_f is the first Piola-Kirchhoff fibril stress, and $\vec{e}_f$ is the current fibril direction.

The mechanical behavior is further refined by integrating an elastic component, represented by an exponential stress-strain relationship, with a viscoelastic component (Wilson et al., 2006a). The collagen fibrils' viscoelasticity is described as a strain-dependent (Charlebois et al., 2004) behavior with the following two-parameter exponential stress-strain relationships of the Zener model:

$$
\begin{aligned}
P_1 &= E_1(e^{k_1 \varepsilon_f} - 1) \quad && \text{for } \varepsilon_f > 0 \\
P_1 &= 0 && \text{for } \varepsilon_f \leq 0
\end{aligned}
\tag{S13}
$$

and

$$
\begin{aligned}
P_2 &= E_2(e^{k_2 \varepsilon_e} - 1) \quad && \text{for } \varepsilon_e > 0 \\
P_2 &= 0 && \text{for } \varepsilon_e \leq 0
\end{aligned}
\tag{S14}
$$

Here, E_1, E_2, k_1, and k_2 are positive material constants; ε_f represents the total fibril strain, while ε_e is the strain in the spring s_2 that interacts in series wit the dash-pot, η. The transient stress includes the dashpot effect that partially relaxes P_2. At equilibrium (i.e., full relaxation), P_2 vanishes completely.

The derivation of P_2 as a function of the fibril strain ε_f is given in Wilson et al. (2006a).

1.0.3 Pressure Component - Osmotic swelling

Characterizing the behavior of the fluid by Darcy's law alone is insufficient. Its response depends on tissue deformations and pressure gradients and is also affected by the chemical fixed charge densities that create swelling driving forces. In the BS model, under free swelling conditions, the volumetric expansion results in an increase in the Jacobian determinant.

The Donan osmotic potential describes swelling behavior (Malandrino et al., 2015), assuming that electrolyte flux can be neglected in mechanical studies of charged materials. Accordingly, the internal and external osmotic pressures are represented by the classical Van't Hoff equation (Huyghe and Janssen, 1997), and assuming that the osmotic components are instantaneously equilibrated with the external bath, the osmotic pressure gradient $\Delta\pi$ is given by (Wilson et al., 2005b):

$$
\Delta\pi = \phi_{\text{int}} RT \left(\sqrt{c_{f,\text{exf}}^2 + 4 \left(\frac{\gamma_{\text{ext}}^{\pm}}{\gamma_{\text{int}}^{\pm}} \right)^2 c_{\text{ext}}^2} \right) - 2\phi_{\text{ext}} RT c_{\text{ext}}
\tag{S15}
$$

where R is the gas constant, and T is the absolute temperature. The internal and external osmotic coefficients ϕ_{int} and ϕ_{ext} multiply the terms related to concentrations of mobile cations and anions, respectively. The average of the internal and external activity coefficients of the ions is represented by $\gamma_{\text{int}}^{\pm}$ and $\gamma_{\text{ext}}^{\pm}$ (assuming $\gamma^{\pm} = \sqrt{\gamma^{+}\gamma^{-}}$). These osmotic and activity coefficients were implemented as Huyghe and Janssen (1997); Huyghe et al. (2003) proposed and as other authors such as (Wilson et al., 2005b), (Schroeder et al., 2007) and Galbusera et al. (2011) adopted in their respective studies. The external concentration of salt and the proteoglycan fixed charge density are denoted by c_{ext} and $c_{f,\text{exf}}$, respectively.

1.0.4 Tissue model parameters and relation to composition measurements

To elucidate the relationship between biphasic/poroelastic models and IVD deformations quantified by J, it's essential to connect the variables in the equations for the non-fibrillar solid matrix's effective stress S11 and the osmotic potential S15. The proteoglycan fixed charge density is determined by the ratio of the normal fixed charge density (c_f) in milliequivalents per milliliter of total fluid to the extra-fibrillar water ($n_{f,\text{exf}}$), as defined by Ruiz et al. (2016):

$$c_{f,\text{exf}} = \frac{n_f c_f}{n_{f,\text{exf}}} \tag{S16}$$

where $n_{f,\text{exf}}$ is derived as:

$$n_{f,\text{exf}} = n_f - \varphi_{ci}\rho_{c,\text{tot}} \tag{S17}$$

In this Equation, φ_{ci} indicates the intrafibrillar water content per unit mass of collagen, and $\rho_{c,\text{tot}}$ signifies the total collagen content as a proportion of the tissue's total wet weight (WW).

To ascertain water content, the initial step involves measuring the tissue sample's wet weight (WW) (Huyghe et al., 2003; Malandrino et al., 2015; Ruiz et al., 2016), followed by lyophilization to obtain the dry weight (DW). These measurements facilitate the calculation of the initial total water content ($n_{f,0}$) and, subsequently, the initial solid fraction and the current fluid fraction:

$$n_{f,0} = \frac{\text{WW} - \text{DW}}{\text{WW}} \tag{S18}$$

$$n_{s,0} = 1 - n_{f,0} \tag{S19}$$

$$n_f = \frac{n_{f,0} - 1 + J}{J} \tag{S20}$$

Thus, using $n_{f,0}$ as a foundational value, the equations seamlessly connect $n_{s,0}$ and n_f with the IVD deformations represented by J, establishing a coherent framework for relating IVD composition measurements to mechanical modeling parameters. Then, the total fluid volume ratio is calculated using the void volume in the medium ($\mathrm{d}V_v$) and the total volume of the medium ($\mathrm{d}V$):

$$n_w = \frac{\mathrm{d}V_v}{\mathrm{d}V} \tag{S21}$$

To estimate the proteoglycan and total collagen contents, previous works propose digesting the dried samples in a papain solution. The digested solutions were then used (i) to determine the content of sulfated glycosaminoglycans (sGAG) through a dimethyl methylene blue (DMMB) assay (Farndale et al., 1986) and (ii) to achieve a measure for collagen content according to hydroxyproline measurements through the chloramine-T assay (Huszar et al., 1980).

We calculated the initial fixed charge density ($c_{f,0}$) per total hydrated tissue volume, from which c_f (Equation S16) is derived to be dependent of J, using the expression (Narmoneva et al., 1999):

$$c_{f,0} = \frac{z_{cs} c_{cs}}{\text{MW}_{cs}} \tag{S22}$$

$$c_f = c_{f,0} \frac{n_{f,0}}{n_{f,0} - 1 + J} \tag{S23}$$

Here, z_{cs}, MW$_{cs}$, and c_{cs} are the valency (2 mEq/mmol), the molecular weight (513,000 μg/mmol), and the concentration (in μg/mL) of chondroitin sulfate, respectively. The sGAG content measured through the DMMB assay is assumed to be equivalent to the chondroitin sulfate content, i.e., c_{cs} is the amount of sGAG divided by the sample's water content. To obtain $\rho_{c,tot}$, in Equation S24, the initial collagen content (μg/mg DW) was estimated from hydroxyproline content by using 7.6 as the mass ratio of collagen to hydroxyproline (Sivan et al., 2006):

$$\rho_{c,tot} = \%\text{hydroxyproline} \cdot 7.6 \tag{S24}$$

See the works of Huyghe et al. (2003); Malandrino et al. (2015); Ruiz et al. (2013, 2016, 2018) for more information on the evolution of the used model.

1.0.5 Permeability

The tissue's AF and NP hydraulic permeability (κ) are strain-dependent according to the following expression as Wilson et al. (2006a) developed and then adapted by (Schroeder et al., 2007) for the IVD:

$$\boldsymbol{\kappa} = \alpha \left(1 - n_{f,\text{exf}}\right)^{-M} \tag{S25}$$

where α stands for the initial permeability at zero strain, and M is a positive constant that governs volumetric strain dependency, and $n_{f,\text{exf}}$ is calculated in Equation S17.

The CEP is also strain-dependent, following this equation (Mow et al., 1980; Argoubi and Shirazi-Adl, 1996):

$$\boldsymbol{\kappa} = \alpha \left[\frac{e(1+e_0)^2}{e_0(1+e)}\right]^2 \exp\left[M\left(\frac{1+e}{1+e_0} - 1\right)\right] \tag{S26}$$

where e is the void ratio, which is the ratio of the current pore volume (i.e., fluid) to the current volume of the solid matrix, and e_0 is the initial void ratio. The void ratio is related to the initial and current water content/porosity of the tissue, $n_{f,0}$ and n_f, according to the following expression:

$$e = \frac{n_f}{1 - n_f} \quad \text{and} \quad e_0 = \frac{n_{f,0}}{1 - n_{f,0}} \tag{S27}$$

2 EVALUATION OF MORPHOLOGICAL FACTORS ON MECHANICAL RESPONSE

Figures corresponding to each of the volumes. Posterior Transition Zone (PTZ), CNP: Center of the Nucleus, Pulposus, and ATZ: Anterior Transition Zone, and surfaces CT: Cartilage Endplate Top, CB: Cartilage Endplate Bottom, used to evaluate the morphological factors on the mechanical variables.

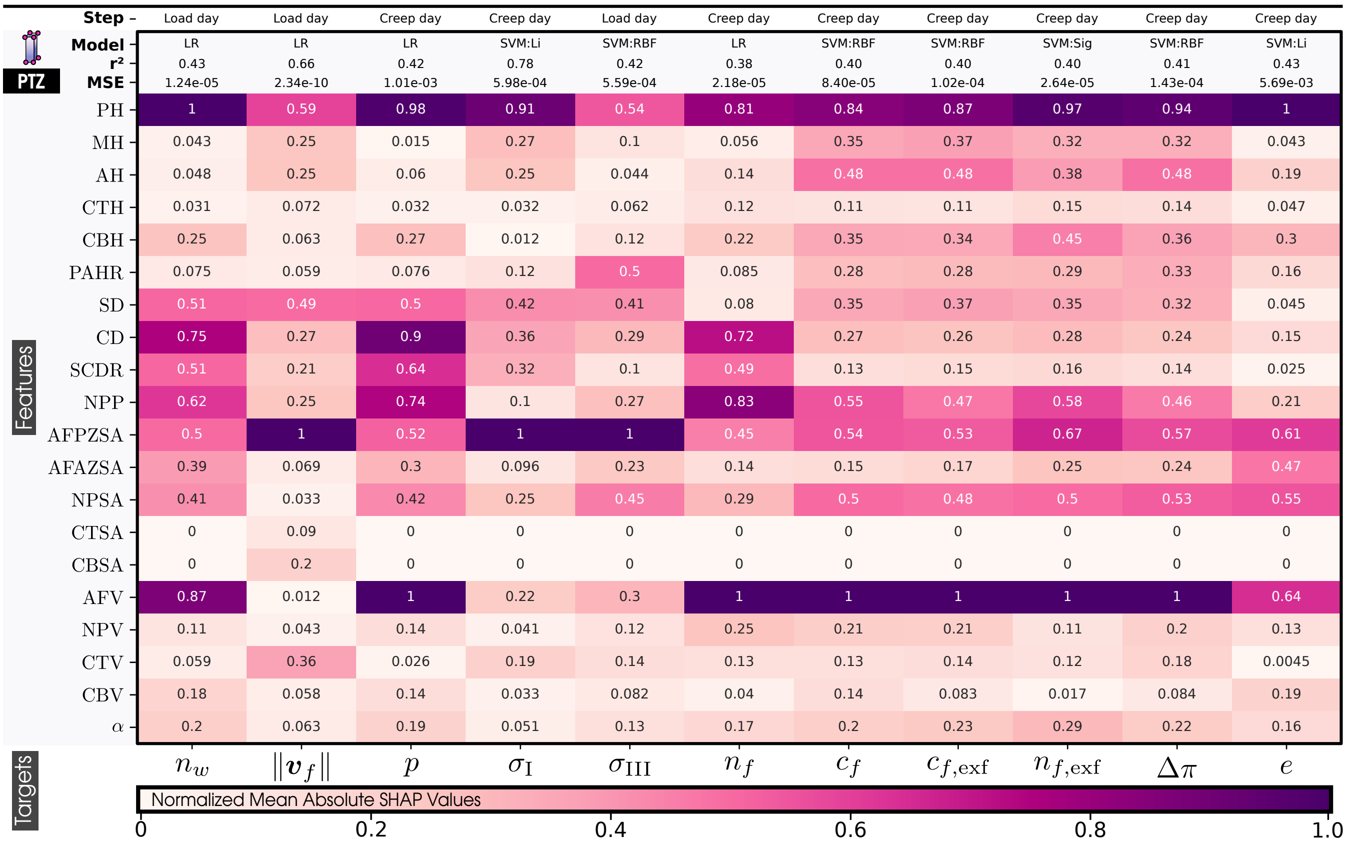

Figure S1. Normalized Mean Absolute SHAP Values on the morphological features (top 5) by their impact on each mechanical response (targets) of our 169 IVD PP models. 20 features are PH: Posterior Height, MH: Middle Height, AH: Anterior Height, CTH: Cartilage Endplate Top Height, CBH: Cartilage Endplate Bottom Height, PAHR: Posterior and Anterior Height Ratio, SD: Sagittal Distance, CD: Coronal Distance, SCDR Sagittal and Coronal Distance Ratio, NPP: Nucleus Pulposus Perimeter, AFPZSA, and AFAZSA: Sagittal Area of both the Posterior Zone and Anterior Zone of Annulus Fibrosus, NPSA: Nucleus Pulposus Sagittal Area, CTSA: Cartilage Endplate Top Sagittal Area, CBSA: Cartilage Endplate Bottom Sagittal Area, AFV, NPV, CTV, and CBV: Volumes of Annulus Fibrosus, Nucleus Pulposus, and both Cartilage Endplates and α: Wedge Angle. 11 targets are n_w: Fluid Volume Ratio, $\|\boldsymbol{v}_f\|$: Maginitude of Pore Fluid Effective Velocity, p: Hydrostatic fluid pressure, σ_{I}: Principal Stress I, σ_{III}: Principal Stress III, n_f: Water Content, c_f: Fixed Charge Density, $c_{f,\mathrm{exf}}$: Extrafibrillar Fixed Charge Density, $n_{f,\mathrm{exf}}$: Extrafibrillar Water Content, $\Delta\pi$: Swelling/Osmotic Pressure, e: Void Ratio. Regression models used, LR: Linear Regression, SVR: Support Vector Machine (*kernel* functions: *linear* (Li), *poly* (Po), *rbf* (RBF), and *sigmoid* (Sig)), and XGBoostR: ExtremeGradient Boosting. PTZ: Posterior Transition Zone.

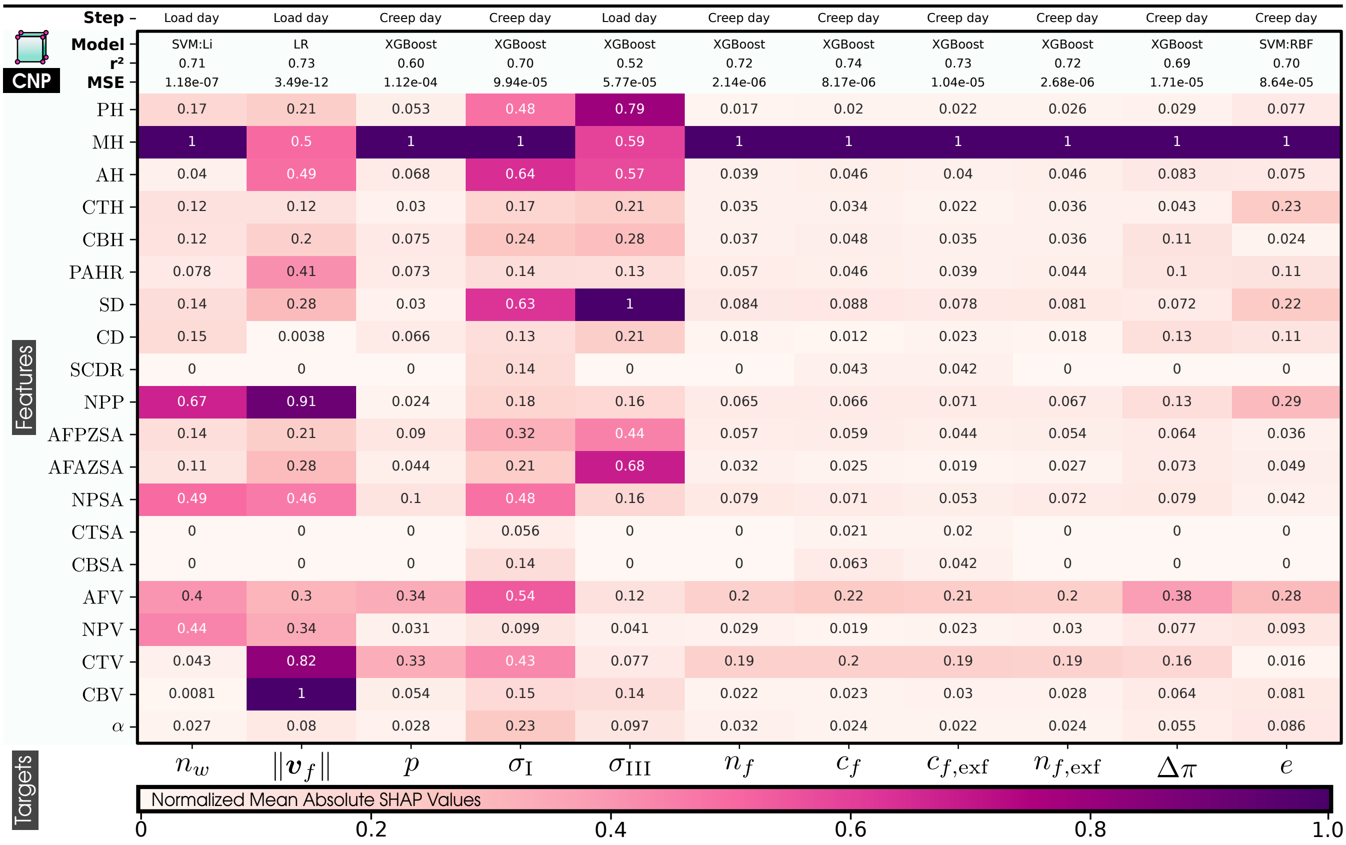

Figure S2. Normalized Mean Absolute SHAP Values on the morphological features (top 5) by their impact on each mechanical response (targets) of our 169 IVD PP models. 20 features are PH: Posterior Height, MH: Middle Height, AH: Anterior Height, CTH: Cartilage Endplate Top Height, CBH: Cartilage Endplate Bottom Height, PAHR: Posterior and Anterior Height Ratio, SD: Sagittal Distance, CD: Coronal Distance, SCDR Sagittal and Coronal Distance Ratio, NPP: Nucleus Pulposus Perimeter, AFPZSA, and AFAZSA: Sagittal Area of both the Posterior Zone and Anterior Zone of Annulus Fibrosus, NPSA: Nucleus Pulposus Sagittal Area, CTSA: Cartilage Endplate Top Sagittal Area, CBSA: Cartilage Endplate Bottom Sagittal Area, AFV, NPV, CTV, and CBV: Volumes of Annulus Fibrosus, Nucleus Pulposus, and both Cartilage Endplates and α: Wedge Angle. 11 targets are n_w: Fluid Volume Ratio, $\|\boldsymbol{v}_f\|$: Maginitude of Pore Fluid Effective Velocity, p: Hydrostatic fluid pressure, σ_{I}: Principal Stress I, σ_{III}: Principal Stress III, n_f: Water Content, c_f: Fixed Charge Density, $c_{f,\mathrm{exf}}$: Extrafibrillar Fixed Charge Density, $n_{f,\mathrm{exf}}$: Extrafibrillar Water Content, $\Delta\pi$: Swelling/Osmotic Pressure, e: Void Ratio. Regression models used, LR: Linear Regression, SVR: Support Vector Machine (*kernel* functions: *linear* (Li), *poly* (Po), *rbf* (RBF), and *sigmoid* (Sig)), and XGBoostR: ExtremeGradient Boosting. CNP: Center of the Nucleus Pulposus.

3 DETAIL IMPACT OF MORPHOLOGICAL FACTORS ON MECHANICAL RESPONSE

Figures corresponding to each of the volumes, Posterior Transition Zone (PTZ), CNP: Center of the Nucleus, Pulposus, and ATZ: Anterior Transition Zone, and surfaces CT: Cartilage Endplate Top, CB: Cartilage Endplate Bottom, used to evaluate how big is the impact of the morphological factors on the mechanical variables.

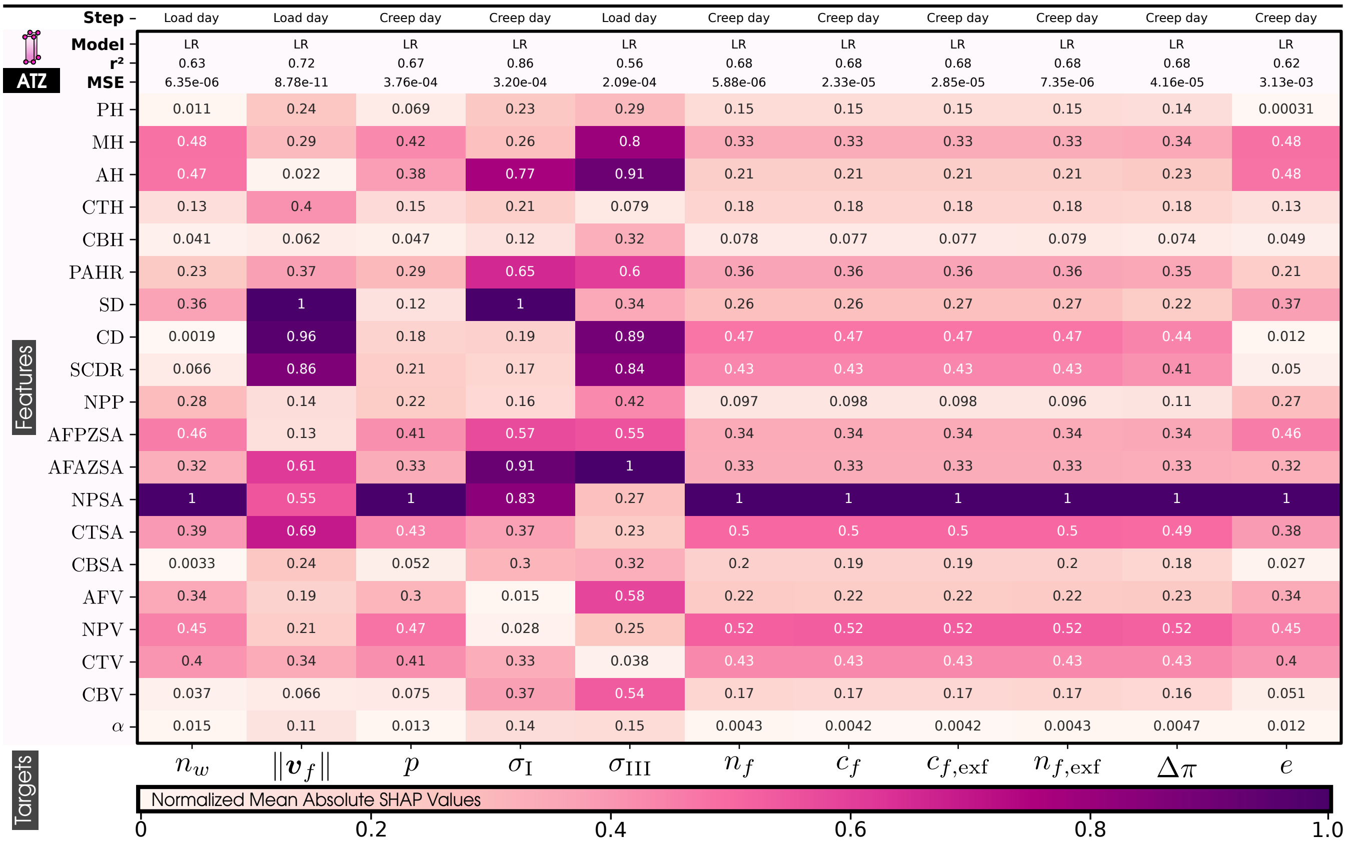

Figure S3. Normalized Mean Absolute SHAP Values on the morphological features (top 5) by their impact on each mechanical response (targets) of our 169 IVD PP models. 20 features are PH: Posterior Height, MH: Middle Height, AH: Anterior Height, CTH: Cartilage Endplate Top Height, CBH: Cartilage Endplate Bottom Height, PAHR: Posterior and Anterior Height Ratio, SD: Sagittal Distance, CD: Coronal Distance, SCDR Sagittal and Coronal Distance Ratio, NPP: Nucleus Pulposus Perimeter, AFPZSA, and AFAZSA: Sagittal Area of both the Posterior Zone and Anterior Zone of Annulus Fibrosus, NPSA: Nucleus Pulposus Sagittal Area, CTSA: Cartilage Endplate Top Sagittal Area, CBSA: Cartilage Endplate Bottom Sagittal Area, AFV, NPV, CTV, and CBV: Volumes of Annulus Fibrosus, Nucleus Pulposus, and both Cartilage Endplates and α: Wedge Angle. 11 targets are n_w: Fluid Volume Ratio, $\|\boldsymbol{v}_f\|$: Maginitude of Pore Fluid Effective Velocity, p: Hydrostatic fluid pressure, σ_{I}: Principal Stress I, σ_{III}: Principal Stress III, n_f: Water Content, c_f: Fixed Charge Density, $c_{f,\mathrm{exf}}$: Extrafibrillar Fixed Charge Density, $n_{f,\mathrm{exf}}$: Extrafibrillar Water Content, $\Delta\pi$: Swelling/Osmotic Pressure, e: Void Ratio. Regression models used, LR: Linear Regression, SVR: Support Vector Machine (*kernel* functions: *linear* (Li), *poly* (Po), *rbf* (RBF), and *sigmoid* (Sig)), and XGBoostR: ExtremeGradient Boosting. ATZ: Anterior Transition Zone

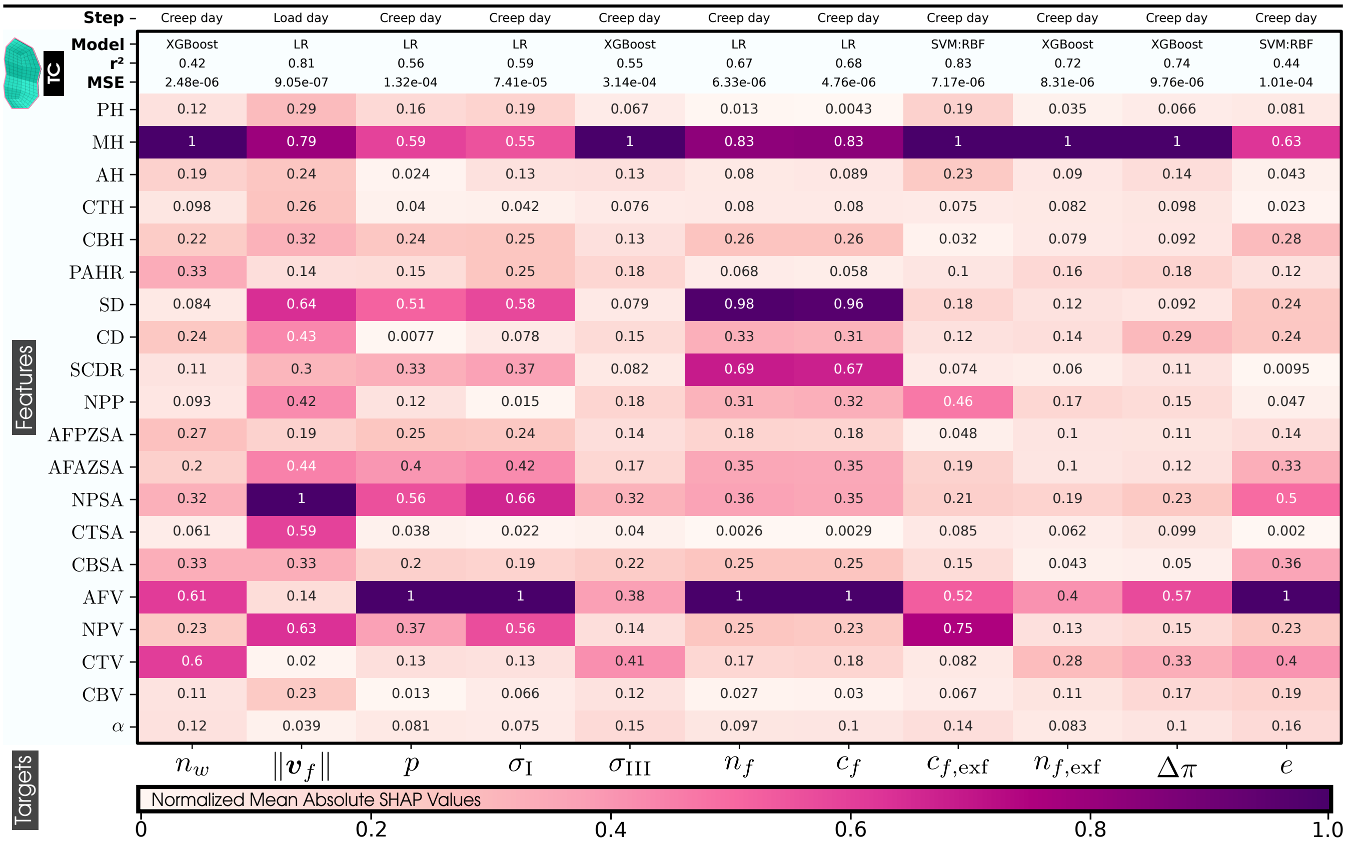

Figure S4. Normalized Mean Absolute SHAP Values on the morphological features (top 5) by their impact on each mechanical response (targets) of our 169 IVD PP models. 20 features are PH: Posterior Height, MH: Middle Height, AH: Anterior Height, CTH: Cartilage Endplate Top Height, CBH: Cartilage Endplate Bottom Height, PAHR: Posterior and Anterior Height Ratio, SD: Sagittal Distance, CD: Coronal Distance, SCDR Sagittal and Coronal Distance Ratio, NPP: Nucleus Pulposus Perimeter, AFPZSA, and AFAZSA: Sagittal Area of both the Posterior Zone and Anterior Zone of Annulus Fibrosus, NPSA: Nucleus Pulposus Sagittal Area, CTSA: Cartilage Endplate Top Sagittal Area, CBSA: Cartilage Endplate Bottom Sagittal Area, AFV, NPV, CTV, and CBV: Volumes of Annulus Fibrosus, Nucleus Pulposus, and both Cartilage Endplates and α: Wedge Angle. 11 targets are n_w: Fluid Volume Ratio, $\|\boldsymbol{v}_f\|$: Maginitude of Pore Fluid Effective Velocity, p: Hydrostatic fluid pressure, σ_{I}: Principal Stress I, σ_{III}: Principal Stress III, n_f: Water Content, c_f: Fixed Charge Density, $c_{f,\mathrm{exf}}$: Extrafibrillar Fixed Charge Density, $n_{f,\mathrm{exf}}$: Extrafibrillar Water Content, $\Delta\pi$: Swelling/Osmotic Pressure, e: Void Ratio. Regression models used, LR: Linear Regression, SVR: Support Vector Machine (*kernel* functions: *linear* (Li), *poly* (Po), *rbf* (RBF), and *sigmoid* (Sig)), and XGBoostR: ExtremeGradient Boosting. CT: Cartilage Endplate Top.

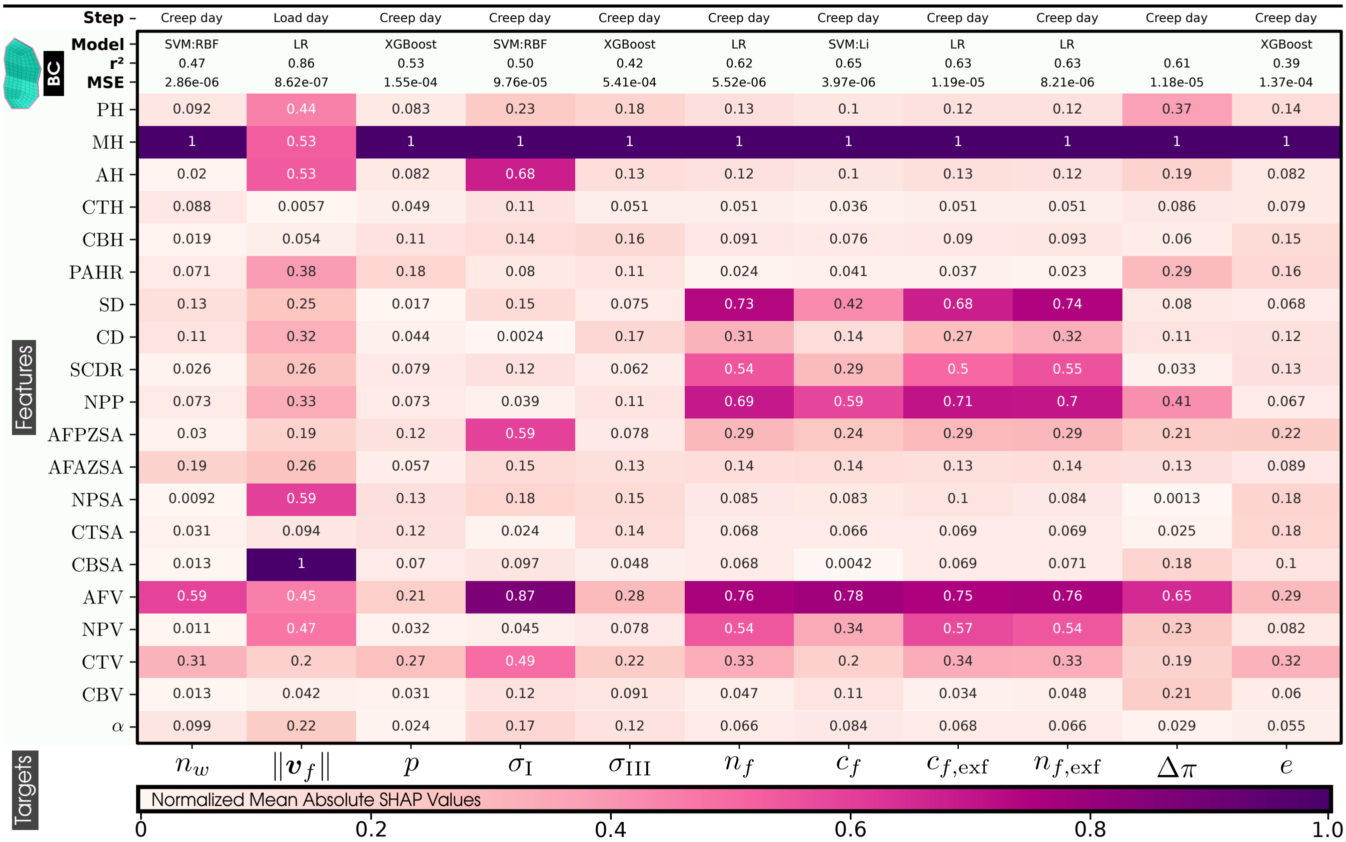

Figure S5. Normalized Mean Absolute SHAP Values on the morphological features (top 5) by their impact on each mechanical response (targets) of our 169 IVD PP models. 20 features are PH: Posterior Height, MH: Middle Height, AH: Anterior Height, CTH: Cartilage Endplate Top Height, CBH: Cartilage Endplate Bottom Height, PAHR: Posterior and Anterior Height Ratio, SD: Sagittal Distance, CD: Coronal Distance, SCDR Sagittal and Coronal Distance Ratio, NPP: Nucleus Pulposus Perimeter, AFPZSA, and AFAZSA: Sagittal Area of both the Posterior Zone and Anterior Zone of Annulus Fibrosus, NPSA: Nucleus Pulposus Sagittal Area, CTSA: Cartilage Endplate Top Sagittal Area, CBSA: Cartilage Endplate Bottom Sagittal Area, AFV, NPV, CTV, and CBV: Volumes of Annulus Fibrosus, Nucleus Pulposus, and both Cartilage Endplates and α: Wedge Angle. 11 targets are n_w: Fluid Volume Ratio, $\|\boldsymbol{v}_f\|$: Maginitude of Pore Fluid Effective Velocity, p: Hydrostatic fluid pressure, σ_{I}: Principal Stress I, σ_{III}: Principal Stress III, n_f: Water Content, c_f: Fixed Charge Density, $c_{f,\mathrm{exf}}$: Extrafibrillar Fixed Charge Density, $n_{f,\mathrm{exf}}$: Extrafibrillar Water Content, $\Delta\pi$: Swelling/Osmotic Pressure, e: Void Ratio. Regression models used, LR: Linear Regression, SVR: Support Vector Machine (*kernel* functions: *linear* (Li), *poly* (Po), *rbf* (RBF), and *sigmoid* (Sig)), and XGBoostR: ExtremeGradient Boosting. CB: Cartilage Endplate Bottom.

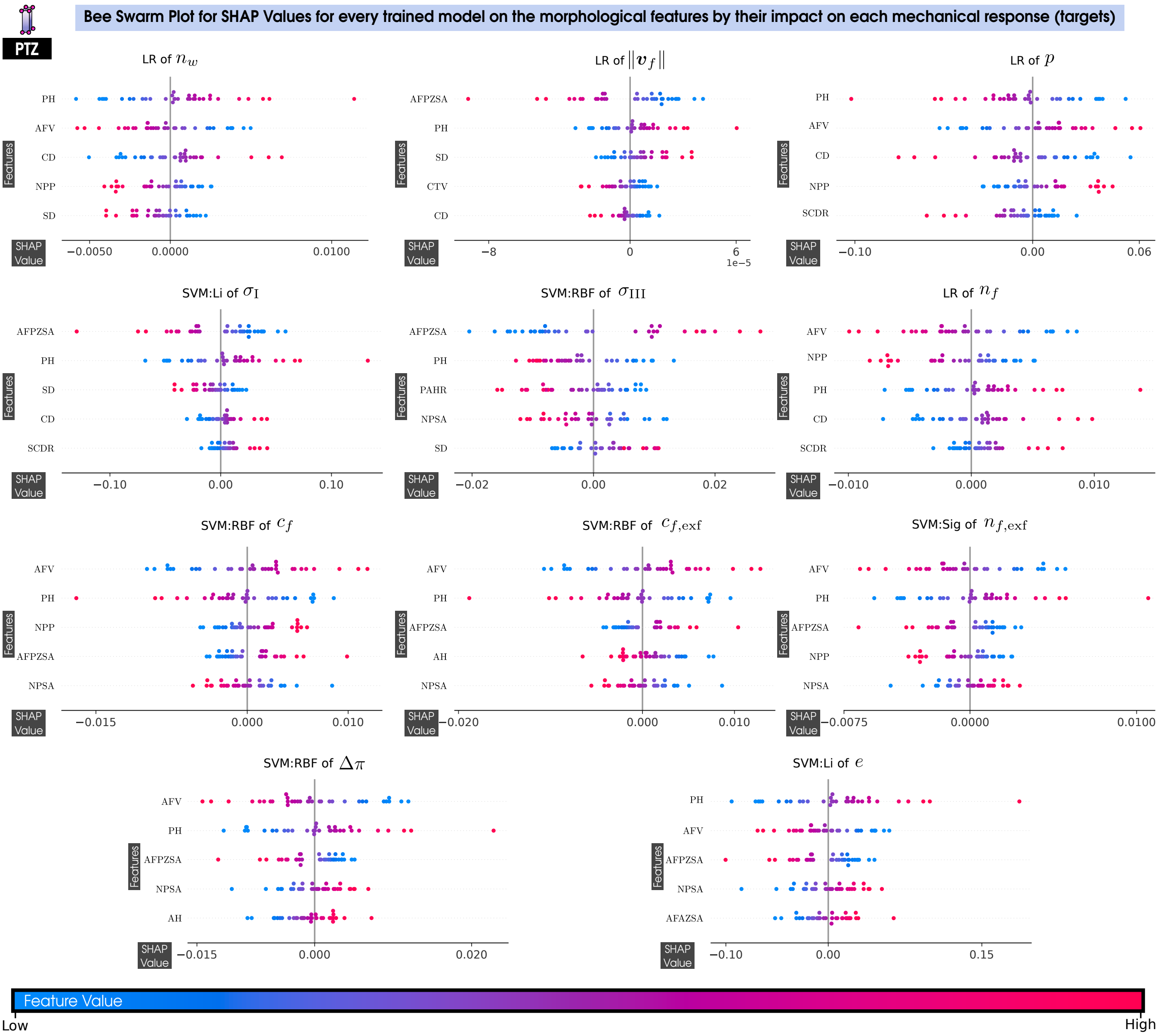

Figure S6. Bee Swarm Plot for SHAP Values for every trained model on the morphological features (top 5) by their impact on each mechanical response (targets) in the Posterior Transition Zone (PTZ). 20 features are PH: Posterior Height, MH: Middle Height, AH: Anterior Height, CTH: Cartilage Endplate Top Height, CBH: Cartilage Endplate Bottom Height, PAHR: Posterior and Anterior Height Ratio, SD: Sagittal Distance, CD: Coronal Distance, SCDR Sagittal and Coronal Distance Ratio, NPP: Nucleus Pulposus Perimeter, AFPZSA, and AFAZSA: Sagittal Area of both the Posterior Zone and Anterior Zone of Annulus Fibrosus, NPSA: Nucleus Pulposus Sagittal Area, CTSA: Cartilage Endplate Top Sagittal Area, CBSA: Cartilage Endplate Bottom Sagittal Area, AFV, NPV, CTV, and CBV: Volumes of Annulus Fibrosus, Nucleus Pulposus, and both Cartilage Endplates and α: Wedge Angle. 11 targets are n_w: Fluid Volume Ratio, $\|\boldsymbol{v}_f\|$: Maginitude of Pore Fluid Effective Velocity, p: Hydrostatic fluid pressure, σ_{I}: Principal Stress I, σ_{III}: Principal Stress III, n_f: Water Content, c_f: Fixed Charge Density, $c_{f,\mathrm{exf}}$: Extrafibrillar Fixed Charge Density, $n_{f,\mathrm{exf}}$: Extrafibrillar Water Content, $\Delta\pi$: Swelling/Osmotic Pressure, e: Void Ratio. Regression models used, LR: Linear Regression, SVR: Support Vector Machine (*kernel* functions: *linear* (Li), *poly* (Po), *rbf* (RBF), and *sigmoid* (Sig)), and XGBoostR: ExtremeGradient Boosting.

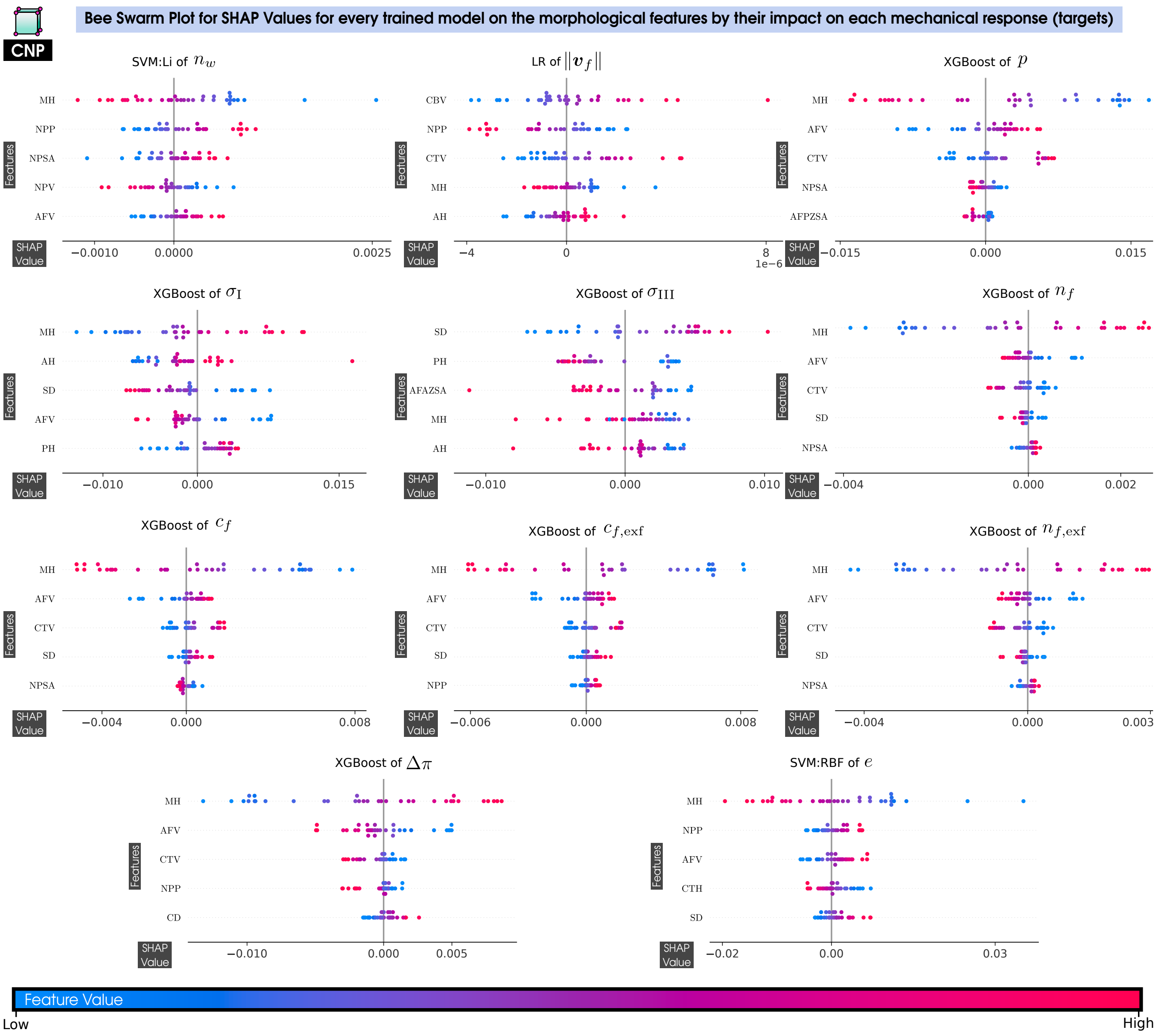

Figure S7. Bee Swarm Plot for SHAP Values for every trained model on the morphological features (top 5) by their impact on each mechanical response (targets) in the Center of the Nucleus Pulposus (CNP). 20 features are PH: Posterior Height, MH: Middle Height, AH: Anterior Height, CTH: Cartilage Endplate Top Height, CBH: Cartilage Endplate Bottom Height, PAHR: Posterior and Anterior Height Ratio, SD: Sagittal Distance, CD: Coronal Distance, SCDR Sagittal and Coronal Distance Ratio, NPP: Nucleus Pulposus Perimeter, AFPZSA, and AFAZSA: Sagittal Area of both the Posterior Zone and Anterior Zone of Annulus Fibrosus, NPSA: Nucleus Pulposus Sagittal Area, CTSA: Cartilage Endplate Top Sagittal Area, CBSA: Cartilage Endplate Bottom Sagittal Area, AFV, NPV, CTV, and CBV: Volumes of Annulus Fibrosus, Nucleus Pulposus, and both Cartilage Endplates and α: Wedge Angle. 11 targets are n_w: Fluid Volume Ratio, $\|\boldsymbol{v}_f\|$: Maginitude of Pore Fluid Effective Velocity, p: Hydrostatic fluid pressure, σ_{I}: Principal Stress I, σ_{III}: Principal Stress III, n_f: Water Content, c_f: Fixed Charge Density, $c_{f,\mathrm{exf}}$: Extrafibrillar Fixed Charge Density, $n_{f,\mathrm{exf}}$: Extrafibrillar Water Content, $\Delta\pi$: Swelling/Osmotic Pressure, e: Void Ratio. Regression models used, LR: Linear Regression, SVR: Support Vector Machine (*kernel* functions: *linear* (Li), *poly* (Po), *rbf* (RBF), and *sigmoid* (Sig)), and XGBoostR: ExtremeGradient Boosting.

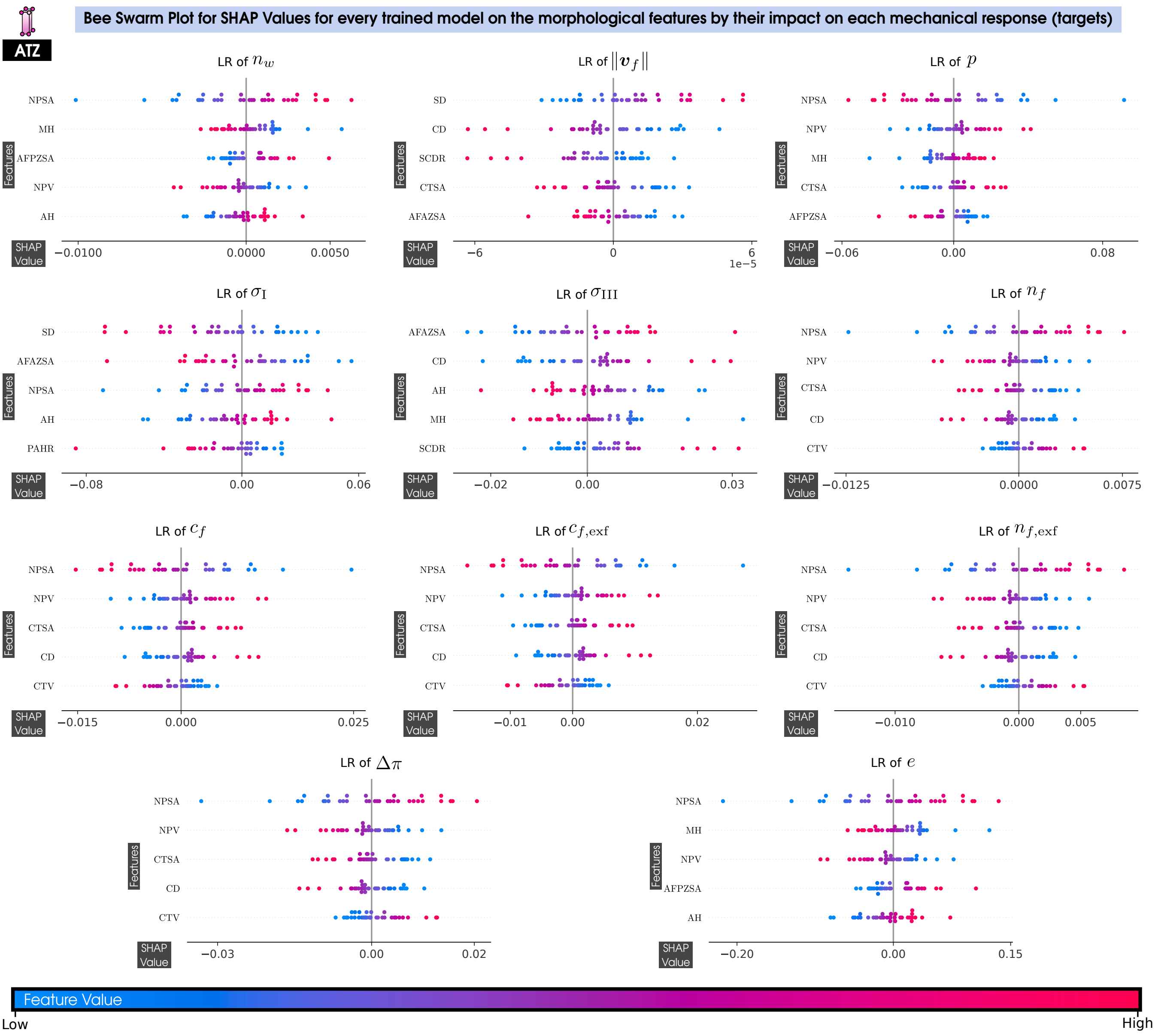

Figure S8. Bee Swarm Plot for SHAP Values for every trained model on the morphological features (top 5) by their impact on each mechanical response (targets) in the Anterior Transition Zone (ATZ). 20 features are PH: Posterior Height, MH: Middle Height, AH: Anterior Height, CTH: Cartilage Endplate Top Height, CBH: Cartilage Endplate Bottom Height, PAHR: Posterior and Anterior Height Ratio, SD: Sagittal Distance, CD: Coronal Distance, SCDR Sagittal and Coronal Distance Ratio, NPP: Nucleus Pulposus Perimeter, AFPZSA, and AFAZSA: Sagittal Area of both the Posterior Zone and Anterior Zone of Annulus Fibrosus, NPSA: Nucleus Pulposus Sagittal Area, CTSA: Cartilage Endplate Top Sagittal Area, CBSA: Cartilage Endplate Bottom Sagittal Area, AFV, NPV, CTV, and CBV: Volumes of Annulus Fibrosus, Nucleus Pulposus, and both Cartilage Endplates and α: Wedge Angle. 11 targets are n_w: Fluid Volume Ratio, $\|v_f\|$: Maginitude of Pore Fluid Effective Velocity, p: Hydrostatic fluid pressure, σ_{I}: Principal Stress I, σ_{III}: Principal Stress III, n_f: Water Content, c_f: Fixed Charge Density, $c_{f,\mathrm{exf}}$: Extrafibrillar Fixed Charge Density, $n_{f,\mathrm{exf}}$: Extrafibrillar Water Content, $\Delta\pi$: Swelling/Osmotic Pressure, e: Void Ratio. Regression models used, LR: Linear Regression, SVR: Support Vector Machine (*kernel* functions: *linear* (Li), *poly* (Po), *rbf* (RBF), and *sigmoid* (Sig)), and XGBoostR: ExtremeGradient Boosting.

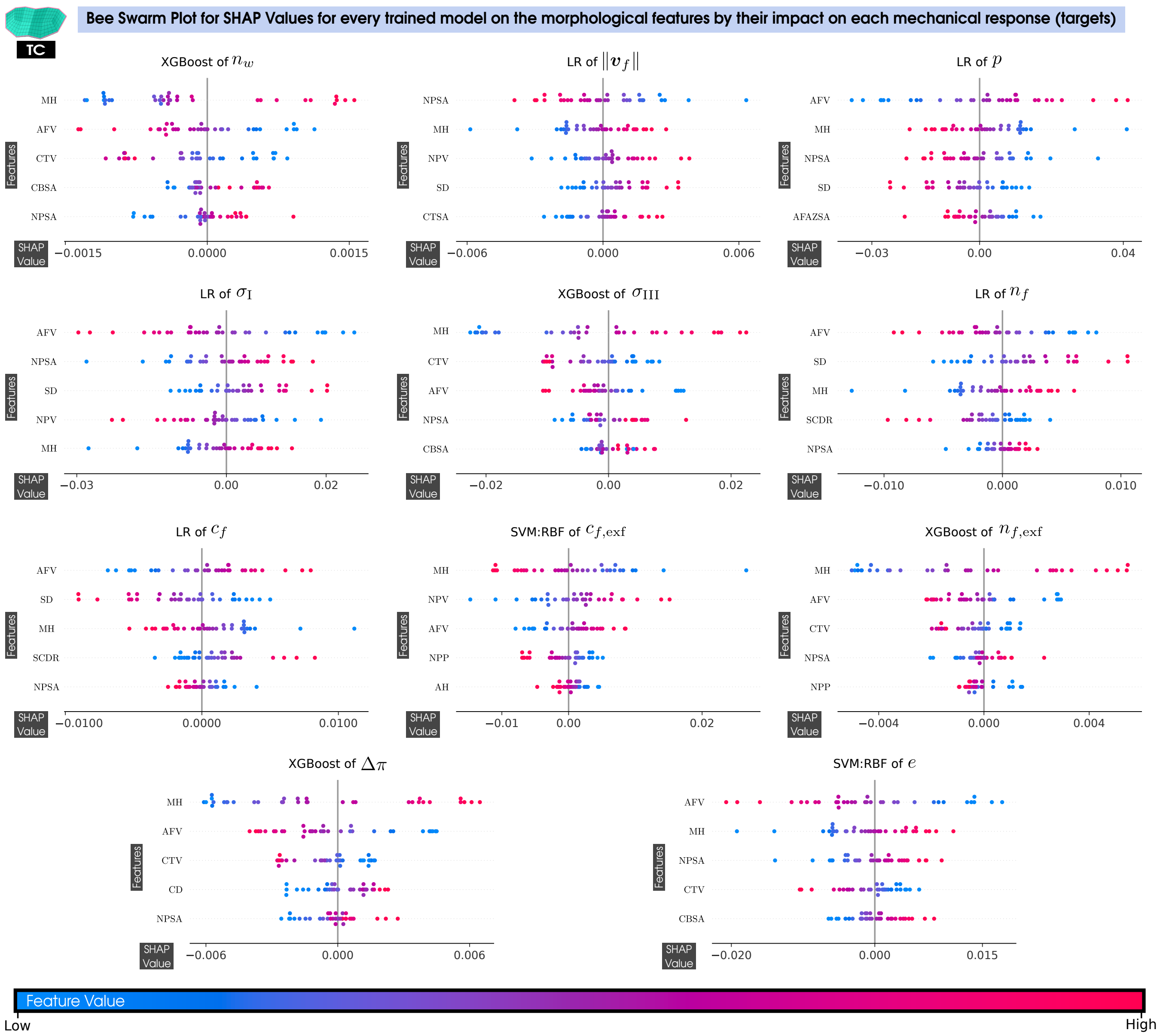

Figure S9. Bee Swarm Plot for SHAP Values for every trained model on the morphological features (top 5) by their impact on each mechanical response (targets) in the Cartilage Endplate Top (CT). 20 features are PH: Posterior Height, MH: Middle Height, AH: Anterior Height, CTH: Cartilage Endplate Top Height, CBH: Cartilage Endplate Bottom Height, PAHR: Posterior and Anterior Height Ratio, SD: Sagittal Distance, CD: Coronal Distance, SCDR Sagittal and Coronal Distance Ratio, NPP: Nucleus Pulposus Perimeter, AFPZSA, and AFAZSA: Sagittal Area of both the Posterior Zone and Anterior Zone of Annulus Fibrosus, NPSA: Nucleus Pulposus Sagittal Area, CTSA: Cartilage Endplate Top Sagittal Area, CBSA: Cartilage Endplate Bottom Sagittal Area, AFV, NPV, CTV, and CBV: Volumes of Annulus Fibrosus, Nucleus Pulposus, and both Cartilage Endplates and α: Wedge Angle. 11 targets are n_w: Fluid Volume Ratio, $\|\boldsymbol{v}_f\|$: Maginitude of Pore Fluid Effective Velocity, p: Hydrostatic fluid pressure, σ_{I}: Principal Stress I, σ_{III}: Principal Stress III, n_f: Water Content, c_f: Fixed Charge Density, $c_{f,\mathrm{exf}}$: Extrafibrillar Fixed Charge Density, $n_{f,\mathrm{exf}}$: Extrafibrillar Water Content, $\Delta\pi$: Swelling/Osmotic Pressure, e: Void Ratio. Regression models used, LR: Linear Regression, SVR: Support Vector Machine (*kernel* functions: *linear* (Li), *poly* (Po), *rbf* (RBF), and *sigmoid* (Sig)), and XGBoostR: ExtremeGradient Boosting.

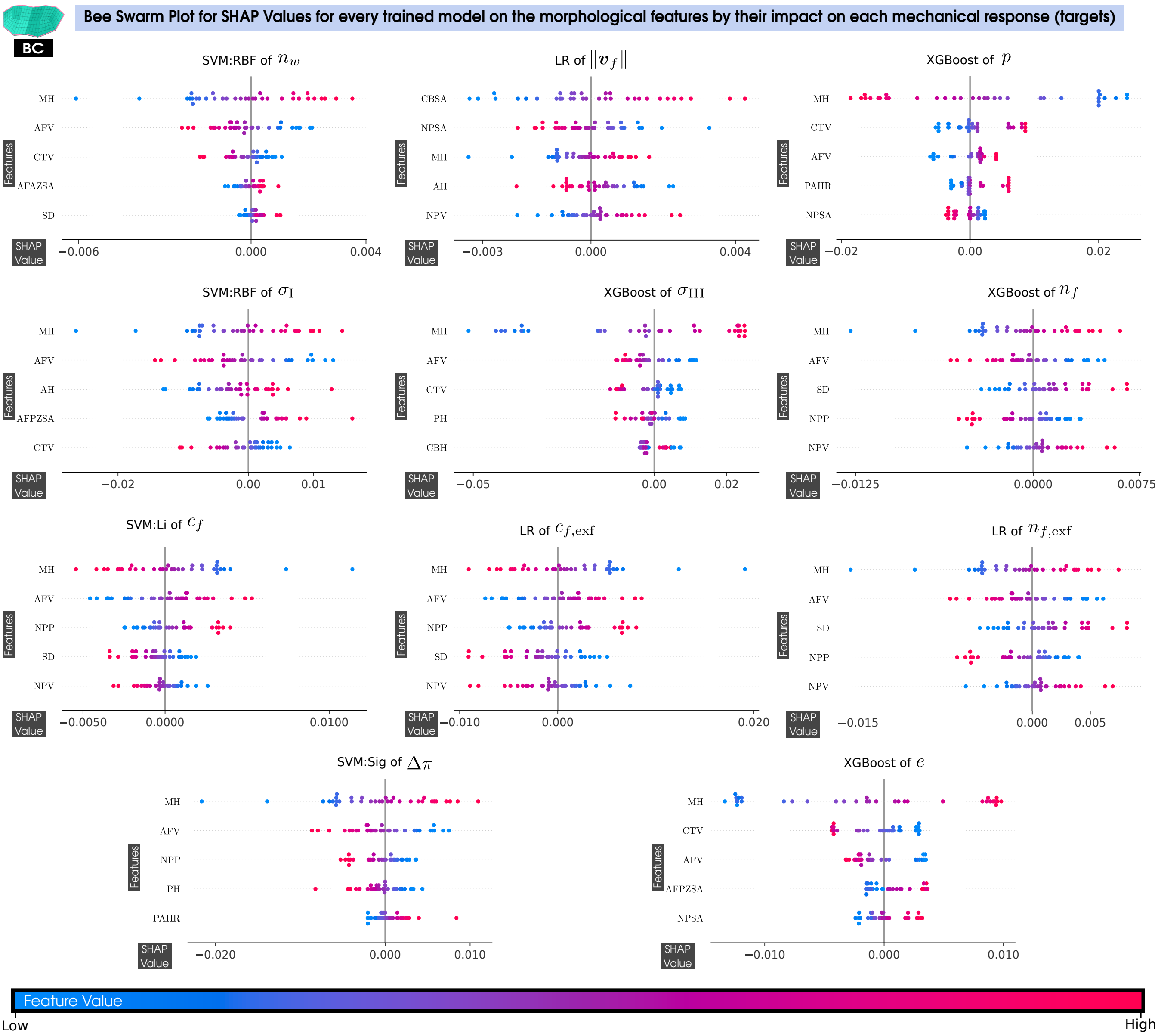

Figure S10. Bee Swarm Plot for SHAP Values for every trained model on the morphological features (top 5) by their impact on each mechanical response (targets) in the Cartilage Endplate Bottom (CB). 20 features are PH: Posterior Height, MH: Middle Height, AH: Anterior Height, CTH: Cartilage Endplate Top Height, CBH: Cartilage Endplate Bottom Height, PAHR: Posterior and Anterior Height Ratio, SD: Sagittal Distance, CD: Coronal Distance, SCDR Sagittal and Coronal Distance Ratio, NPP: Nucleus Pulposus Perimeter, AFPZSA, and AFAZSA: Sagittal Area of both the Posterior Zone and Anterior Zone of Annulus Fibrosus, NPSA: Nucleus Pulposus Sagittal Area, CTSA: Cartilage Endplate Top Sagittal Area, CBSA: Cartilage Endplate Bottom Sagittal Area, AFV, NPV, CTV, and CBV: Volumes of Annulus Fibrosus, Nucleus Pulposus, and both Cartilage Endplates and α: Wedge Angle. 11 targets are n_w: Fluid Volume Ratio, $\|\boldsymbol{v}_f\|$: Maginitude of Pore Fluid Effective Velocity, p: Hydrostatic fluid pressure, σ_{I}: Principal Stress I, σ_{III}: Principal Stress III, n_f: Water Content, c_f: Fixed Charge Density, $c_{f,\text{exf}}$: Extrafibrillar Fixed Charge Density, $n_{f,\text{exf}}$: Extrafibrillar Water Content, $\Delta\pi$: Swelling/Osmotic Pressure, e: Void Ratio. Regression models used, LR: Linear Regression, SVR: Support Vector Machine (*kernel* functions: *linear* (Li), *poly* (Po), *rbf* (RBF), and *sigmoid* (Sig)), and XGBoostR: ExtremeGradient Boosting.

REFERENCES

Argoubi, M. and Shirazi-Adl, A. (1996). Poroelastic creep response analysis of a lumbar motion segment in compression. *Journal of Biomechanics* 29, 1331–1339. doi:10.1016/0021-9290(96)00035-8

Charlebois, M., McKee, M. D., and Buschmann, M. D. (2004). Nonlinear tensile properties of bovine articular cartilage and their variation with age and depth. *Journal of Biomechanical Engineering* 126, 129–137. doi:10.1115/1.1688771

Farndale, R., Buttle, D., and Barrett, A. (1986). Improved quantitation and discrimination of sulphated glycosaminoglycans by use of dimethylmethylene blue. *Biochimica et Biophysica Acta (BBA) - General Subjects* 883, 173–177. doi:10.1016/0304-4165(86)90306-5

Galbusera, F., Schmidt, H., Noailly, J., Malandrino, A., Lacroix, D., Wilke, H.-J., et al. (2011). Comparison of four methods to simulate swelling in poroelastic finite element models of intervertebral discs. *Journal of the Mechanical Behavior of Biomedical Materials* 4, 1234–1241. doi:10.1016/j.jmbbm.2011.04.008

Huszar, G., Maiocco, J., and Naftolin, F. (1980). Monitoring of collagen and collagen fragments in chromatography of protein mixtures. *Analytical Biochemistry* 105, 424–429. doi:10.1016/0003-2697(80) 90481-9

Huyghe, J. and Janssen, J. (1997). Quadriphasic mechanics of swelling incompressible porous media. *International Journal of Engineering Science* 35, 793–802. doi:10.1016/s0020-7225(96)00119-x

Huyghe, J. M., Houben, G. B., Drost, M. R., and van Donkelaar, C. C. (2003). An ionised/non-ionised dual porosity model of intervertebral disc tissue. *Biomechanics and Modeling in Mechanobiology* 2, 3–19. doi:10.1007/s10237-002-0023-y

Malandrino, A., Jackson, A. R., Huyghe, J. M., and Noailly, J. (2015). Poroelastic modeling of the intervertebral disc: A path toward integrated studies of tissue biophysics and organ degeneration. *MRS Bull.* 40, 324–332

Malandrino, A., Noailly, J., and Lacroix, D. (2011). The effect of sustained compression on oxygen metabolic transport in the intervertebral disc decreases with degenerative changes. *PLoS Computational Biology* 7, e1002112. doi:10.1371/journal.pcbi.1002112

Mow, V., Gibbs, M., Lai, W., Zhu, W., and Athanasiou, K. (1989). Biphasic indentation of articular cartilage—ii. a numerical algorithm and an experimental study. *Journal of Biomechanics* 22, 853–861. doi:10.1016/0021-9290(89)90069-9

Mow, V. C., Kuei, S. C., Lai, W. M., and Armstrong, C. G. (1980). Biphasic creep and stress relaxation of articular cartilage in compression: Theory and experiments. *Journal of Biomechanical Engineering* 102, 73–84. doi:10.1115/1.3138202

Narmoneva, D., Wang, J., and Setton, L. (1999). Nonuniform swelling-induced residual strains in articular cartilage. *Journal of Biomechanics* 32, 401–408. doi:10.1016/s0021-9290(98)00184-5

Ruiz, C., Foata, B., González Ballester, M. A., Karppinen, J., and Noailly, J. (2018). Theoretical explorations generate new hypotheses about the role of the cartilage endplate in early intervertebral disk degeneration. *Frontiers in Physiology* 9. doi:10.3389/fphys.2018.01210

Ruiz, C., Malandrino, A., van Rijsbergen, M., Lacroix, D., Ito, K., and Noailly, J. (2016). Simulating the sensitivity of cell nutritive environment to composition changes within the intervertebral disc. *Journal of the Mechanics and Physics of Solids* 90, 108–123. doi:10.1016/j.jmps.2016.02.003

Ruiz, C., Noailly, J., and Lacroix, D. (2013). Material property discontinuities in intervertebral disc porohyperelastic finite element models generate numerical instabilities due to volumetric strain variations. *Journal of the Mechanical Behavior of Biomedical Materials* 26, 1–10. doi:https://doi.org/10.1016/j.jmbbm.2013.05.012

Schroeder, Y., Elliott, D., Wilson, W., Baaijens, F., and Huyghe, J. (2008). Experimental and model determination of human intervertebral disc osmoviscoelasticity. *Journal of Orthopaedic Research* 26, 1141–1146. doi:10.1002/jor.20632

Schroeder, Y., Sivan, S., Wilson, W., Merkher, Y., Huyghe, J. M., Maroudas, A., et al. (2007). Are disc pressure, stress, and osmolarity affected by intra- and extrafibrillar fluid exchange? *Journal of Orthopaedic Research* 25, 1317–1324. doi:10.1002/jor.20443

Sivan, S., Merkher, Y., Wachtel, E., Ehrlich, S., and Maroudas, A. (2006). Correlation of swelling pressure and intrafibrillar water in young and aged human intervertebral discs. *Journal of Orthopaedic Research* 24, 1292–1298. doi:10.1002/jor.20144

Urban, J., Maroudas, A., Bayliss, M., and Dillon, J. (1979). Swelling pressures of proteoglycans at the concentrations found in cartilaginous tissues. *Biorheology* 16, 447–464. doi:10.3233/bir-1979-16609

Urban, J. P. G. and Maroudas, A. (1981). Swelling of the intervertebral discin vitro. *Connective Tissue Research* 9, 1–10. doi:10.3109/03008208109160234

Wilson, W., Huyghe, J., and van Donkelaar, C. (2006a). A composition-based cartilage model for the assessment of compositional changes during cartilage damage and adaptation. *Osteoarthritis and Cartilage* 14, 554–560. doi:10.1016/j.joca.2005.12.006

Wilson, W., Huyghe, J. M., and van Donkelaar, C. C. (2006b). Depth-dependent compressive equilibrium properties of articular cartilage explained by its composition. *Biomechanics and Modeling in Mechanobiology* 6, 43–53. doi:10.1007/s10237-006-0044-z

Wilson, W., van Donkelaar, C., van Rietbergen, B., and Huiskes, R. (2005a). A fibril-reinforced poroviscoelastic swelling model for articular cartilage. *Journal of Biomechanics* 38, 1195–1204. doi:10.1016/j.jbiomech.2004.07.003

Wilson, W., van Donkelaar, C. C., and Huyghe, J. M. (2005b). A comparison between mechano-electrochemical and biphasic swelling theories for soft hydrated tissues. *Journal of Biomechanical Engineering* 127, 158–165. doi:10.1115/1.1835361
